# Reduction in nutritional quality and growing area suitability of common bean under climate change induced drought stress in Africa

**DOI:** 10.1038/s41598-018-33952-4

**Published:** 2018-11-01

**Authors:** Marijke Hummel, Brendan F. Hallahan, Galina Brychkova, Julian Ramirez-Villegas, Veronica Guwela, Bartholomew Chataika, Edna Curley, Peter C. McKeown, Liam Morrison, Elise F. Talsma, Steve Beebe, Andy Jarvis, Rowland Chirwa, Charles Spillane

**Affiliations:** 1 Plant & AgriBiosciences Research Centre (PABC), Ryan Institute, National University of Ireland Galway, University Road, Galway, H91 REW4 Ireland; 2 International Center for Tropical Agriculture (CIAT), Km. 17 Recta Cali-Palmira A. A., 6713 Cali, Colombia; 3 CGIAR Research Program on Climate Change, Agriculture and Food Security (CCAFS), Cali, Colombia; 4 Pan African Bean Research Alliance (PABRA), International Center for Tropical Agriculture (CIAT), P.O. Box 158, Lilongwe, Malawi; 5 Division of Human Nutrition and Health, Wageningen University, P.O. Box 17 6700 AA, Wageningen, The Netherlands

## Abstract

Climate change impacts on food security will involve negative impacts on crop yields, and potentially on the nutritional quality of staple crops. Common bean is the most important grain legume staple crop for human diets and nutrition worldwide. We demonstrate by crop modeling that the majority of current common bean growing areas in southeastern Africa will become unsuitable for bean cultivation by the year 2050. We further demonstrate reductions in yields of available common bean varieties in a field trial that is a climate analogue site for future predicted drought conditions. Little is known regarding the impact of climate change induced abiotic stresses on the nutritional quality of common beans. Our analysis of nutritional and antinutritional compounds reveals that iron levels in common bean grains are reduced under future climate-scenario relevant drought stress conditions. In contrast, the levels of protein, zinc, lead and phytic acid increase in the beans under such drought stress conditions. This indicates that under climate-change induced drought scenarios, future bean servings by 2050 will likely have lower nutritional quality, posing challenges for ongoing climate-proofing of bean production for yields, nutritional quality, human health, and food security.

## Introduction

Dietary deficiencies of micronutrients such as iron and zinc constitute major public health problems globally, particularly amongst women and children in sub-Saharan Africa^1^. While micronutrient supplementation and food fortification are important for improving delivery of micronutrients, staple food crop biofortification through breeding provides an additional route for increasing the supply of key micronutrients (iron, zinc, vitamin A) from staple crops to the diets of poorer communities in developing countries^2–5^. The level of micronutrients (e.g. iron, zinc) in staple crops and foods is one of the key determinants of the extent of uptake of dietary micronutrients^6,7^. However, the presence and levels of anti-nutritionals, in particular phytic acid and polyphenols, can inhibit bioavailability and hence the level of uptake of such micronutrients^8–15^. The consideration of anti-nutritionals in biofortification breeding programs is important to ensure that efforts to increase the levels of micronutrients (e.g. iron) in crops are not compromised by inadvertent increases in levels of anti-nutritionals (such as phytic acid and/or polyphenols) that could arise from breeding efforts^9,11,13,15,16^ or from environmental stresses.

Previous studies have shown a negative impact of predicted mid-century elevated CO_2_ levels on iron and zinc levels of C3 grain and legume crop plants^17^, which is anticipated to aggravate the extent of iron deficiency in human diets globally^18^. In addition to increased CO_2_ levels, reduced and erratic rainfall will lead to increases in the incidence and frequency of drought in some regions, which in turn will lead to reductions in crop yields^19^. For common bean, it is not known whether drought-associated reductions in crop yields will also lead to changes in the nutritional quality of beans under future climate change induced drought scenarios^20^.

In this study, we have used Ecocrop to model the impact of climate change induced changes in heat and precipitation by 2050, on the suitability for cultivation of common bean across a range of countries in southeastern Africa. In addition, we have combined the climate impact modeling with experimental field trials of common bean, under the extent of drought anticipated due to future climate change (by 2050), to determine the impact on both yield and the nutritional quality of common bean under climate-induced drought stress. Our results are important for efforts to climate proof cultivation of the staple crop common bean, so that varieties can be developed, which under drought stress maintain good yields and contain high levels of the dietary micronutrients iron and zinc, while containing low levels of anti-nutritional factors such as lead and phytic acid.

## Results

The majority of current common bean growing areas in southeastern Africa will become unsuitable for bean cultivation by the year 2050 due to changes in temperature and precipitation.

To determine climate change impacts on common bean (*Phaseolus vulgaris*) suitability for cultivation in southeastern Africa, the EcoCrop model was used to produce spatially-explicit simulations of potential climatic suitability for five countries in south-eastern Africa. For each spatial unit (i.e. grid cell), EcoCrop performs separate calculations for temperature-limited (heat and cold) and precipitation-limited (waterlogging and drought) suitability, and then calculates an overall suitability for the crop^21,22^.

**Figure 1:**
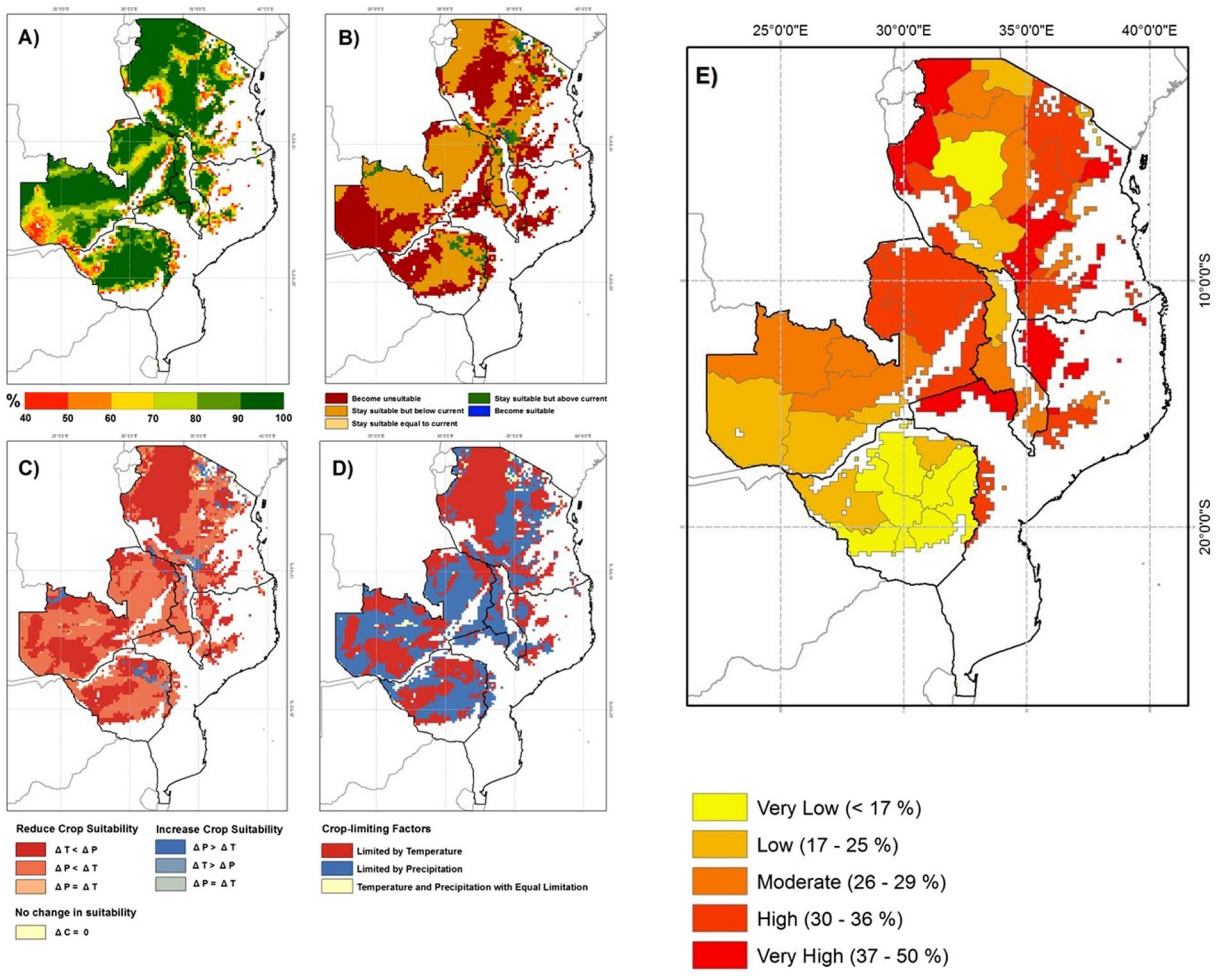
Historical and future (2050) common bean suitability simulations for south-east Africa and current percentage of children underweight; (**A**) Suitability of currently cultivated common bean for historical climate; (**B**) Projected impact of climate change by 2050 s; (**C**) Driving factor of change in future climatic suitability; (**D**) factor most limiting to bean cultivation suitability by 2050; (**E**) Percentage of children, under the age of 5, who are underweight (data from CIESIN), for the period 1990–2002. Red (reduced suitability), blue (increased suitability), and beige (no change in suitability) colours are used in (**C**) to separate directions of change. In red areas, shades of red are used to differentiate areas where suitability reductions are due to temperature changes (ΔT < ΔP), from those where suitability reductions are due to precipitation changes (ΔP < ΔT) or where temperature- and precipitation- suitability reductions are equal (ΔP = ΔT). In blue areas, shades of blue are used to differentiate areas where suitability increases are primarily driven by precipitation (ΔP > ΔT), temperature (ΔT > ΔP), or equally driven by both (ΔP = ΔT).

Figure 1 shows the spatial distribution of historical climatic suitability for common bean cultivation (Fig. 1A), as well as the spatial distribution of projected climate change impacts on bean cultivation by 2050 (Fig. 1B). The unshaded areas within the countries analysed (Malawi, Mozambique, Tanzania, Zambia and Zimbabwe) are considered unsuitable for bean cultivation (Fig. 1A). Currently, suitable areas for bean cultivation extend across most of Zambia, Zimbabwe, Tanzania, western Malawi, and northern Mozambique (Fig. 1A). Our simulated historical climatic suitability for common bean cultivation agrees well with the observed distribution of bean cultivation in Africa^23^. Our EcoCrop modelling of future climate impacts on bean cultivation suitability indicates that a significant proportion of the currently suitable areas will become unsuitable for common bean cultivation by 2050 (red areas in Fig. 1B), particularly in southern Zambia, eastern Zimbabwe, and central Tanzania. In these areas, unless appropriate climate adaptation actions (e.g. climate smart agriculture (CSA) breeding and agronomy options such as new varieties or irrigation) are put in place, it will no longer be possible to grow common beans.

### Common bean cultivation suitability differs across locations within each country dependent on changes in temperature or precipitation

Our results further indicate that a reduction in the temperature-related suitability resulting from increased heat stress by 2050 is predicted as the main cause (ΔT < ΔP) for the overall reduction in climatic suitability of bean growing areas in north western Tanzania, southern Zambia, and western Zimbabwe (dark red shading in Fig. 1C). Conversely, reductions in precipitation-related suitability by 2050 were found to be the major cause (ΔP < ΔT) of climatic suitability change reductions in western Malawi, northern Zambia, eastern Tanzania, and southern Zimbabwe (orange shading in Fig. 1C). Figure 1D demonstrates where future bean cultivation suitability by 2050 will be predominantly limited by temperature (red), precipitation (blue) or both temperature and precipitation (yellow).

### Reductions in yields of common bean varieties at a climate analogue field site that is representative of predicted drought conditions by 2050

To determine the impacts of future drought scenarios by 2050 on the yields of common bean, we conducted a field trial in western Malawi of 20 common bean varieties over two growing seasons. The bean varieties used consisted of varieties commonly cultivated by smallholder farmers and also bean lines developed by the CIAT and PABRA bean breeding programs under consideration for entry into the national varietal registration and official release process. They comprise a range of different market classes (i.e. quality types) including black, red, kidney, mottled red, brown, light brown and white beans, both smaller and larger sized seed varieties. These varieties were chosen to reflect the reality of (a) what bean varieties are actually available to smallholder farmers in Malawi and (b) what bean varieties are in the regulatory pipeline in Malawi that may become accessible to smallholders within the next decade, taking account of the time-lag expected for national varietal registration, certification processes and bulking up of seed by suppliers. The suitability of the field trial site area in western Malawi for common bean cultivation is projected to fall below current suitability levels by 2050, primarily due to decreased precipitation (Fig. 1B,C).

All twenty bean varieties were field trialed under both rainfed conditions and drought-stress conditions that are representative of conditions predicted by EcoCrop modeling to occur by 2050 (Fig. S1). This corresponded to four field trial seasons in total, i.e. two trials under rainfed conditions and two under drought-stress conditions. To ensure seasons could be reliably and reproducibly grouped according to “rainfed” and “drought-stress” conditions, *k*-means cluster analysis was performed on recorded weather data for the field trial site. The identified clusters were analysed using discriminant analysis for the goodness of fit of the model, and t-statistics were applied to verify the predicted *vs*. found mean of the clusters (Supplementary Results File). In addition, to further verify the climate change relevance of our drought-stress growing seasons, Simulated Weather Data for Crop Modelling and Risk Assessment (MarkSim) software V.2 was employed (http://www.ccafs-climate.org/pattern_scaling/). These analyses confirmed that our drought-stress field trial conditions can be considered as a climate analogue site for bean growing seasonal weather conditions in the year 2095 in Malawi, under RCP 8.5. Average temperatures during our drought field trials were 22 °C with extremes of 35 °C, closely resembling the predicted average temperatures for the bean growing season (March, April and May) of 24 °C and extremes of 32 °C in the year 2095. Likewise, the average rainfall during the bean grain filling period in 2015 was 54 mm, similar to the projected average rainfall of 58 mm (Fig. S2).

To determine whether the reduction in yield of the common bean varieties under drought-stress conditions at the climate analogue trial site was more influenced by genotype (variety) or weather conditions (temperature, precipitation), an F-test was performed. This revealed that the variation in yield observed at the trial site is influenced more by weather conditions than genotype (Supplementary Results File). To determine whether there are any statistically significant differences between the varieties under drought stress conditions, a one-way ANOVA was performed, which showed no significant yield difference between the bean genotypes (Supplementary Results File). Similarly, no significant yield differences were identified between the bean genotypes under rainfed conditions (Supplementary Results File). Overall, for all bean varieties that were field trialed at the Malawi climate analogue site, the common bean grain yield decreased by an average of 43% under drought-stress conditions, which is significantly lower (two-tailed independent samples t-test, *P* < 0.001) when compared with rainfed conditions (Fig. S3).

### While iron levels in bean grains decrease, under climate-scenario relevant drought stress conditions, zinc, lead, protein and phytic acid levels increase

Many of the countries analysed in this study have moderate to high child underweight rates (Fig. 1E)^24^. Micronutrient deficiencies are major contributors to the global problem of maternal and child malnutrition, which causes underweight, stunting and wasting conditions in afflicted children^1,25^. To determine the changes in levels of dietary micronutrients between rainfed and drought-stress conditions of each common bean variety, we used inductively coupled plasma-mass spectrometry (ICP-MS) to measure the relative concentrations of twenty-two elements (B, Na, Mg, P, S, K, Ca, Ti, Cr, Mn, Fe, Co, Ni, Cu, Zn, As, Se, Rb, Sr, Mo, Cd and Pb), which included the important dietary micronutrients iron and zinc, and also antinutritional compounds such as lead (Table S1; Fig. S4). For each common bean variety under rainfed and drought conditions, we also determined the protein levels and the levels of phytic acid, a major antinutritional limiting micronutrient uptake from human diets^26^ (Table S1; Fig. S4).

As seen for yield, the variations in iron, zinc, lead, protein and phytic acid under drought-stress conditions at the field site are more influenced by weather conditions than genotype (variety) (Supplementary Results File). To further investigate the lack of variation among genotypes for iron, zinc, lead, protein and phytic acid, a one-way ANOVA was performed. When we compared across genotypes under rainfed conditions we found no significant difference between genotypes for iron, zinc, lead, protein and phytic acid content. Similarly, when we compared across genotypes under drought conditions we found no significant difference between genotypes for iron, lead, protein and phytic acid content. Under drought-stress conditions, one variety shows significantly higher (*P* < 0.05) zinc levels than the other nineteen varieties, but at the 5% significance level such a result would be expected by chance (Supplementary Results File). We also compared our results for concentrations of Zn and Fe for the twenty varieties tested under rainfed or drought conditions with those recorded for the 1000 accessions in the CIAT cultivated common bean core collection (Fig. S5) and find them to be representative of the range of Fe levels, and representative (or slightly enriched) for Zn. The responses of Fe and Zn in our tested varieties are therefore likely to be similar to those of other common bean genotypes.

**Figure 2:**
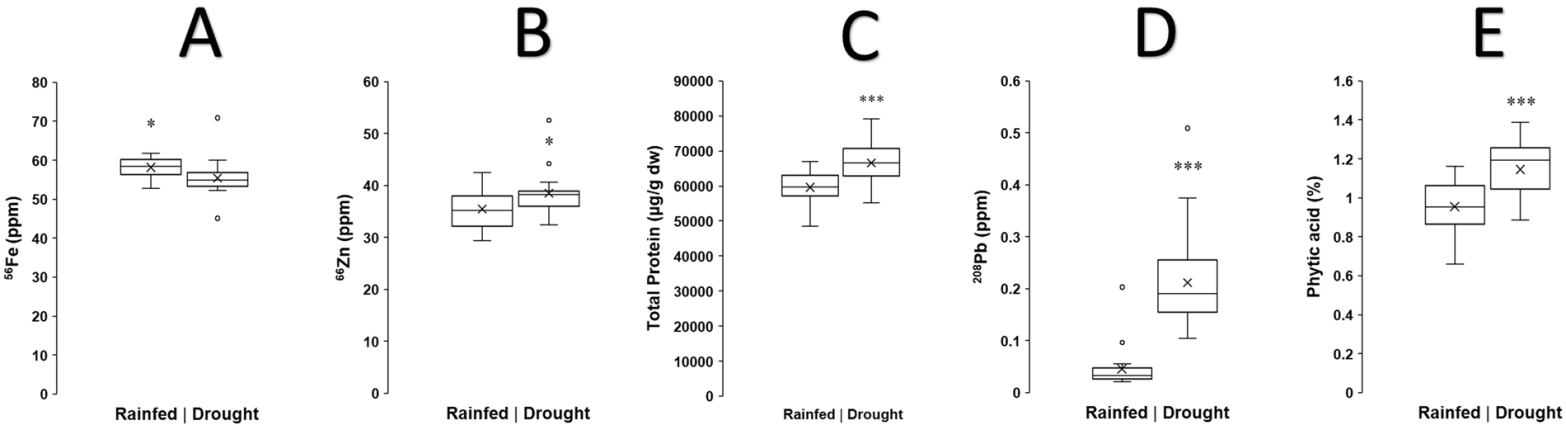
Box and whisker plot showing average grain iron (**A**), zinc (**B**), protein (**C**), lead (**D**), and phytic acid (**E**) levels of 20 common bean varieties grown under rainfed and drought-stress conditions. ‘X’ Indicates mean value. ^*^P < 0.05 ^***^P < 0.001.

Overall, across all of the twenty bean varieties analysed, the levels of iron are significantly reduced under drought stress (two-tailed independent samples t-test, *P* < 0.05), from an average concentration of 59 ppm in beans from rainfed plants to 54 ppm from drought-stressed plants (Fig. 2A). Conversely, the average concentration of zinc significantly increases under drought stress (two-tailed independent samples t-test, *P* < 0.05), from 35 ppm in beans from rainfed plants to 39 ppm from drought-stressed plants, (Fig. 2B). We identify a significant increase (two-tailed independent samples t-test, *P* < 0.001) in lead levels in the bean grains under drought-stress conditions, from an average concentration of 0.05 ppm to 0.22 ppm, representing a fourfold increase (Fig. 2C). This exceeds the maximum level permissible (0.1 ppm) for pulses as defined by the Codex Alimentarius^22^. The average total protein concentration also significantly increases under drought stress (two-tailed independent samples t-test, *P* < 0.001) from 326 mg/g of dry weight in beans from rainfed plants to 371 mg/g of dry weight in beans from drought-stressed plants (Fig. 2D). Similarly, there was a significant increase (two-tailed independent samples t-test, *P* < 0.001) in phytic acid levels under drought stress, from an average level of 0.96% under rainfed conditions to 1.16% under drought stress (Fig. 2E).

### Changes in precipitation and temperature correlate with changes in yield of common bean, and also with iron, lead and protein levels, but not zinc and phytic acid levels

To further investigate the link between weather conditions during the grain filling period and common bean growth, multiple linear regression analysis was performed. As the differences in rainfall between the rainfed seasons and the drought-stress seasons were also accompanied by differences in air temperature (Fig. S1), the impact of temperature was included as well as rainfall. Over 50% of the variation in yield and iron could be explained by changes in temperature and rainfall between flowering date and harvest. Approx. 40% of the variation in lead could be explained by changes in temperature that occurred between flowering date and harvest, while rainfall that occurred during this time window did not improve the model. Approx. 30% of the variation in protein levels could be explained by changes in temperature and rainfall between flowering date and harvest. Finally, weather conditions during the grain filling period only explained a small part of the variation in zinc (4.0%) or phytic acid (17%), suggesting that other factors not included in the model influence zinc and phytic acid levels in the grain (Supplementary Results File).

### Under climate-change induced drought scenarios, future bean servings will have lower nutritional quality

**Figure 3:**
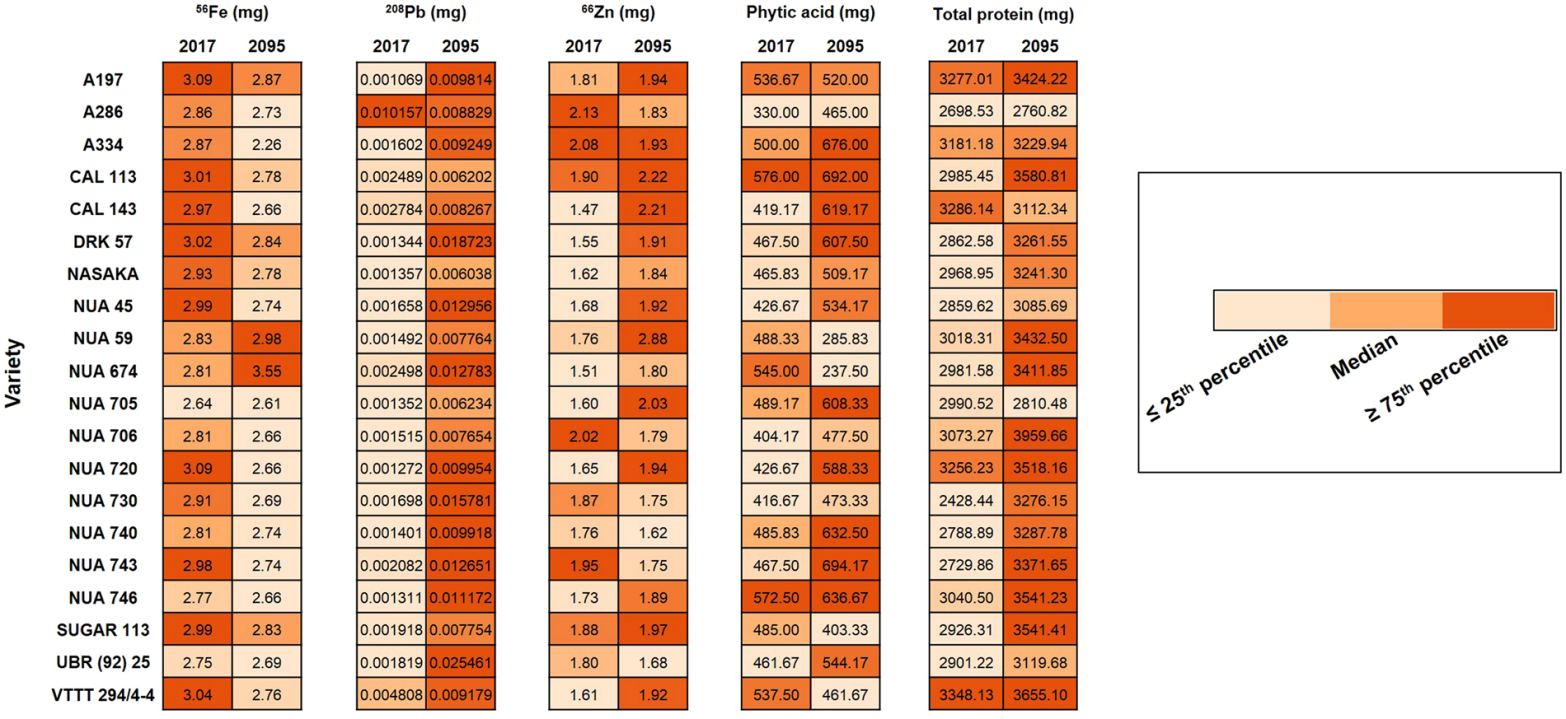
Heat map of nutritional quantity from one serving of beans harvested from present-day (2017) and predicted future (2095) conditions. Concentration of nutrients (^56^Fe, ^66^Zn, total protein) and anti-nutritionals (^208^Pb, phytic acid) in a 50 g serving of 20 common bean varieties under rainfed and drought conditions was calculated. Median, lower and upper quartile values were calculated for ^56^Fe, ^66^Zn, protein, ^208^Pb and phytic acid separately.

While yield is typically measured in kg/hectare, the nutritional yield can be considered as the quantity of supply of nutritionally-important compounds per unit area^27^. To determine the supply of nutritional and anti-nutritional compounds on a per meal basis, we calculated the quantity of each dietary compound that each common bean variety would deliver per serving (50 g) under present day (rainfed) and future climate (drought-stress) scenarios (Fig. 3). This highlights that while future bean servings under climate change may become more zinc-rich, they will contain less iron and more undesirable anti-nutritionals (lead and phytic acid). Our results indicate that some varieties (NUA 59, NUA 674) may display promise for drought-proofing dietary supplies from beans under predicted future climate-change scenarios.

## Discussion

Climate change represents a threat to food security, particularly resulting from ongoing and anticipated negative impacts on agricultural productivity (yields/hectare)^28–32^. While there have been a range of studies which indicate negative impacts on yields of major staple crops^33–35^, there have been fewer studies that have investigated the impact of climate change stresses (e.g. rising CO_2_, heat, drought) on staple crop grain quality parameters^17,36–40^.

Common beans are the most important grain legume supporting food security and human nutrition globally, responsible for almost 15% of daily calories and 36% of daily protein in some countries in Africa and the Americas^41^. In sub-Saharan Africa, common beans are an important staple crop for smallholder farmers and a key nutritional component in diets of poor rural communities. Smallholder farmers in sub-Saharan Africa plant a wide range of bean varieties and landraces that they access from multiple sources, including via purchasing from formal (e.g. government distributors, commercial seed companies, agro-dealers, NGO/UN) or informal (e.g. local markets, own seed stocks, neighbor) sources^42–44^. Such smallholder farming communities are extremely vulnerable to negative impacts of climate change on their livelihoods and nutritional status, including through reductions in yields and/or nutritional quality of staple crops they both consume and trade^45,46^.

Climate change can change weather patterns, resulting in altered temperature and rainfall effects in different regions, which can have concomitant impacts on the suitability of crops for continued cultivation in climate-change impacted regions^47^. In particular, elevated temperatures (heat) and reduced rainfall (drought) can reduce crop yields^28,48–50^. Our EcoCrop climatic suitability analyses for common bean in South Eastern Africa to 2050 indicates that predicted increases in temperature and reductions in rainfall (precipitation) will result in common bean becoming unsuitable for cultivation across the vast majority of current bean growing regions (Fig. 1D). Only in specific localised zones in northern Zimbabwe, southern and northern Tanzania, and northern Malawi, are increases in climatic suitability for bean cultivation projected. Relocation of bean cultivation to different areas beyond the current range of cultivation may be possible, but in this case careful consideration should be given to choosing varieties suited for the new area, including any change in photoperiod that occurs with changing latitude. Overall, our findings are consistent with those of previous studies where different models to EcoCrop have been used^51–53^. Future climate conditions will be associated with elevated atmospheric CO_2_ concentrations, which may either exacerbate or alleviate the effects of increased temperature and reduced precipitation on common bean growing regions. While the EcoCrop model cannot process atmospheric CO_2_ effects, it is noteworthy that C3 plants (of which common bean is one) have been shown experimentally to respond to drought by reducing photosynthesis, an effect which is not removed upon doubling CO_2_ treatment^54^. We conclude that, in the absence of implementation of significant adaptation strategies to maintain yields of common bean in southeastern Africa, it can be expected that yields of common bean will dramatically decline across the region in the period to 2050. The adaptation strategies that will be necessary to implement at scale may be incremental (e.g. breeding new bean varieties or using agronomic practices such as irrigation) or transformational (e.g. involving changing to a different protein or high-value crop species, or finding an alternative livelihood that is more climate-resilient)^55^.

In addition to yield reductions that will negatively impact on livelihoods, there is potential for climate stresses to also impact on the nutritional quality of crops. Such climate effects may frustrate biofortification efforts to breed new biofortified varieties of staple crops that have elevated levels of essential micronutrients^6^. Screening of over 1000 genotypes of common bean germplasm from the CIAT^56^ genebank revealed an average Fe concentration of 55 ppm, within a concentration range of 34 and 89 ppm. The average Zn concentration was 35 ppm, within a concentration range of 21 and 54ppm. Notably, all 20 genotypes investigated in our study have similar Fe and Zn concentrations. The average Fe concentration among genotypes used in our study (i.e. 59ppm) is statistically similar to the average observed across the primary genepool (i.e. 55 ppm). Likewise the average Zn concentration among the genotypes in our study (i.e. 35 ppm) is identical to the average across the genepool (i.e.35 ppm) (Fig. 2A,B, Fig. S5, Supplementary Results File).

While there has been a previous attempt to determine the effect of water-limiting conditions on Fe and Zn levels in common bean, using a limited number of genotypes under irrigated conditions, further studies are required to determine impacts on nutritional availability under drought in key bean growing regions^41–44^. In our study we have modeled the negative impacts of climate change on common bean production in southeastern Africa, which has revealed that reduced precipitation by 2050 will be the main limiting factor for bean cultivation. Furthermore, we conducted a multi-year field trial at a climate analogue site which experiences weather conditions similar to that predicted for Malawi in the year 2095. Our results are the first to demonstrate that the level of a key nutrient (i.e. iron) in common beans under climate change induced drought stress will significantly decline.

Studies conducted to date on iron bioavailability from high-Fe biofortified beans using Caco-2 cell models are congruent with findings from poultry models^12,57^, which in turn are consistent with human feeding trials which have shown positive nutritional impacts from consumption of high-Fe biofortified beans^9,26,58–60^. However, antinutritional compounds in staple crop plants can negatively affect the uptake (bioavailability) of nutritional compounds (e.g. iron, zinc)^61^. Such antinutritional compounds include phytic acid and polyphenols, which have been shown to negatively affect the bioavailability and uptake of iron and zinc from common beans, using *in vitro* (Caco-2 cell) and animal (poultry) models^9,11–13,15,16,26,57^. In addition, the composition of targeted diets or meal plans can affect the extent of iron uptake from high-Fe biofortified beans. For instance, some foods commonly consumed with beans (e.g. rice) can inhibit Fe bioavailability while others (e.g potato) can increase Fe bioavailability when eaten with beans^9^.

In this study we have focused on the effect of phytic acid because of its major influence on iron bioavailability, especially in the case of consuming beans in a composite meal^26^. However, we recognise that polyphenols are an additional class of anti-nutritionals that need to be considered in high-Fe bean biofortification efforts. For instance, studies in black beans have shown that total polyphenols inhibit iron uptake in Caco-2 cell assays^15^. The overall inhibitory effect of polyphenols is combinatorial, whereby some polyphenols (catechin, 3,4-dihydroxybenzoic acid, kaempferol, and kaempferol 3-glucoside) promote iron uptake while others (myricetin, myricetin 3-glucoside, quercetin, and quercetin 3-glucoside) inhibit iron uptake^15^. Because of differential potency effects between polyphenols that inhibit or promote iron uptake, it is considered that the majority of the inhibitory polyphenol compounds would need to be removed from biofortified beans in order to substantially reduce the inhibitory effect on iron uptake^16^.

There are also a number of heavy metals (e.g. lead, cadmium, arsenic), which can enter the human body via diet, that can act as toxic anti-nutritionals (depending on concentration)^62^. In addition to drought-induced reductions in the levels of iron, our results demonstrate that drought-stressed common bean varieties also display increases in the levels of the antinutritional compounds phytic acid and lead. While we did not measure the levels of polyphenols, polyphenol levels can increase in plants in response to drought-stress^63,64^, and have been shown to negatively affect iron and zinc bioavailability and uptake from dietary common bean^11,15^.

The underlying physiological basis for the increases of antinutritional compounds such as phytic acid and lead under drought stress are unclear. In tropical soils, such as laterites, it has been shown that sorption values for Pb^2+^ are greater than those of other bivalent cations^65^. The development of deeper rooting systems in arid soils could potentially lead to greater contact with Pb^2+^ cations adsorbed within the soil. Alternatively, the observed increase could be a secondary effect of greater investment in active cation uptake when the soluble fraction of nutrient cations is insufficient to meet plant needs, reminiscent of the increased uptake of Al^3+^ cations that can be observed in calcicole plants under acidic conditions. However, we cannot exclude other impacts of drought and heat on root physiology^66^, and direct analysis of root growth in common bean under different climatic conditions will be required to distinguish these possibilities.

The increases in phytic acid are of particular concern as it is considered the main anti-nutritional compound in legumes. The increased phytic acid accumulation likely relates to its function in limiting oxidative stress in legumes under dryer conditions^67,68^. Indeed, phytic acid is known to accumulate in legume seeds (e.g. chickpeas) in response to drought stress^69,70^. It should be noted however that phytic acid in field peas has been found to be reduced under elevated CO_2_ levels^17^. If a similar response occurs in common beans then the increase in phytic acid levels we observe could be counteracted. Growth trials combining changes in climate and CO_2_ simultaneously will be needed to assess interactions between possibly competing effects.

Our study indicates that ongoing efforts to develop biofortified bean varieties will need to not only develop heat- and drought-tolerant beans, but will also need to ensure that such varieties also maintain elevated iron and zinc levels, and low levels of antinutritional compounds (e.g. phytic acid, lead and specific inhibitory polyphenols) under drought or other environmental stresses. To avoid unintended consequences, our results highlight that it is critically important that biofortified crop varieties (including under abiotic stresses) do not accumulate anti-nutritionals (e.g. phytic acid, lead, arsenic, polyphenols)^71^. Overall, our results demonstrate that there will be a reduction in the nutritional quality of a typical bean serving, if the common bean varieties have been cultivated under the levels of drought stress predicted for southeastern Africa to 2050 and beyond.

## Conclusions

Both incremental and transformational climate change adaptation^32,52,55,72^ strategies are needed for common bean cropping systems of smallholder farmers in south-eastern Africa, whereby such farmers can have greater access to improved varieties and agronomic practices that allows their cropping systems to be more resilient to increased heat and drought conditions, while maintaining or improving nutritional composition of bean grains. Recent plant breeding progress to develop drought- and heat-adapted bean varieties indicates that genetics-based adaptation should be possible^73^, which can be a component of an overall portfolio of climate smart agriculture technologies and practices to ensure resilient of common bean cultivation to climate change impacts^74^. Where farmers have access to (and widely adopt) such improved bean varieties^44^, it may be possible to maintain yields in areas where cultivation suitability will be negatively impacted by climate change^72^. As the breeding, testing and dissemination of new bean varieties can take a decade or more, our results highlight the need for accelerated development and seed-system dissemination of heat- and drought-tolerant common bean varieties that can maintain yields while also improving nutritional quality (e.g. via biofortification breeding) under future climate change scenarios.

## Methods

### EcoCrop Modelling of drought and heat impacts on common bean in East Africa

For a more detailed description of the EcoCrop model the reader is referred to Ramirez-Villegas *et al*.^22^, and Hijmans *et al*.^21^. For previous assessments climate change impacts using EcoCrop for various crops (including common beans) in Africa the reader is referred to Rippke *et al*.^72^ and Jarvis *et al*.^75^.

EcoCrop suitability simulations for common bean were performed for a historical period (1960–1990, chosen to be a representative baseline) and then for the 2050 period (2040–2069) under the Representative Concentrations Pathway 6.0 (RCP 6.0)^76^. RCP 6.0 was chosen since it is representative of a business as usual scenario. Climate data used for historical suitability simulations was derived from WorldClim^77^, which is a global high resolution database of monthly climatological means for mean, maximum and minimum temperatures and total precipitation. For each future period, simulations were performed using 19 Global Climate Model (GCM) projections, statistically downscaled and bias-corrected^78^. For both historical and future simulations, we assume that the common bean crop is not viable when the overall suitability is below 43%^72^.

EcoCrop is a relatively simple crop suitability model and as such is subject to various limitations, most notably, it does not include estimates of extreme climate impacts, soil fertility, pest and diseases, all of which can be critical to bean growth^79,80^. It also does not provide information on crop productivity. Whilst the exclusion of these limitations likely means we underestimate climate change-related challenges for bean production, we argue that they are useful to identify key adaptation priorities for the region.

### Plant material

The common bean (*Phaseolus vulgaris*) varieties used in this experiment included released varieties in Malawi, a landrace and also a range of unreleased advanced lines from the CIAT’s bean biofortification breeding program. This consisted of eight BC1F4 Andean Nutrition (NUA) bean lines (NUA 743, NUA 720, NUA 674, NUA 730, NUA 746, NUA 705, NUA 706 and NUA 740) and nine released varieties (A268, A197, CAL 143, A344, NUA 45, SUGAR 131, CAL 113, VTTT 294/4–4, NUA 59, UBR (92)25 and DRK 57) and one landrace (Nasaka).

### Field trials

All twenty bean varieties were field trialed under both rainfed conditions and drought-stress conditions that are representative of conditions predicted by Ecocrop modeling to occur by 2050. The drought and rainfed trials of all bean varieties were conducted between 2013–2015 at the Kandiyani site of the International Center for Tropical Agriculture (CIAT) Chitedze Research Station, Lilongwe, Malawi. The crop trial was laid out in a Randomized Complete Block Design (RCBD) with three plots/replicates. Two seasons per year the beans were harvested, in the rainy season, plants were grown under natural, rainfed conditions between December and March. The drought trial was conducted in the winter/off-season which lasts from late August until November.

For each plot, four ridges were prepared, measuring 5 m long and spaced at 0.60 m. The net plot comprised of the two middle ridges with the outer ridges acting as borders. One seed was planted per planting station spaced at 0.50 cm. No fertilizer was applied in the trials. Pests and diseases were controlled using Dimethoate (Aryzta LifeScience, South Africa) and Karate (Syngenta, Basel, Switzerland) according to product label recommendations. Agronomic data collected from each plot consisted of; days to 50% flowering; days to 95% physiological maturity; plant height at harvesting; grain weight; total plot yield. Yield was determined by weight of grains.

### Sampling of bean pods and grains for nutritional analysis and sample preparation

Bean samples from four harvests in the period 2013–2015 were analysed. Bean pods were sampled when mature, from every plot 10 plants were randomly selected and of each of these plants 10 pods were taken (on different sides of the plant and not touching the soil). All procedures were followed according to HarvestPlus protocols for crop sampling to ensure samples were representative and minimize any risk of sample contamination^81^.

After harvest, all samples were kept in a storage room in CIAT, Malawi at room temperature until transfer to the National University of Ireland Galway (NUI Galway). On arrival in NUI Galway, beans were removed from pods and stored at −20 °C in plastic sample bags until further processing. Bean samples were then freeze-dried and a subsample of 20 beans (i.e. grains) were milled to a fine powder using a coffee grinder (Delonghi, KG49). Samples were vacuum-packed until ICP-MS analysis in Ionomics Lab, University of Aberdeen, Aberdeen, Scotland.

### ICP-MS analysis of elemental composition of common bean grains

Briefly, bean powder of each sample was analysed in duplicate with inductively coupled plasma-mass spectrometry (ICP-MS) as follows: trace metal grade nitric acid was added to the samples. Samples were then spiked with an internal standard plus hydrogen peroxide and left overnight to pre-digest. Following exposure to 115 °C for 4 hours, digested samples were diluted with Milli-Q water. Aliquots were transferred to a 96-well plate for analysis. A detailed description of ICP-MS analysis is provided in the Supplementary Information.

### Protein extraction and measurement

Bean proteins were extracted in three separate stages^82,83^, namely aqueous extraction (albumins) followed by saline extraction (globulins), followed by cell-lysis extraction (other, membrane-bound proteins). Microcentrifuge tubes (2 ml) were prepared with ten 1 mm glass beads (Sigma-Aldrich) inside. For aqueous extraction bean powder was suspended in distilled water (50 mg in 400 µl; 1:8), shaken in a tissue lyser (QIAGEN) at maximum speed for 30 seconds, followed by incubation at 4 °C for 30 min (samples were shaken every 10 minutes). The sample was centrifuged at 14000 rpm for 15 min. The supernatant was transferred to new tubes. Next, the water-insoluble fraction was extracted from the sediment. The sample was treated as before, but 0.5 M NaCl solution was used instead of distilled water. The supernatant was transferred to new tubes. This should result in 75% of total protein extraction. To release all membrane-bound proteins from the sediment, the sample was treated as before but Lysis buffer (50 mM citric acid, pH 3.0, 1 M NaCl, 2% SDS, 0.5% Triton-X-100) was used instead of NaCl solution or distilled water. The supernatant was transferred to new tubes; this is expected to result in ≥91% of total protein extraction.

Samples were measured using the BCA protein assay (Thermo Fisher Scientific) on 96-well plates in triplicate. Readings were taken at 560 nm wavelength (Modulus Microplate Mulitmode Reader, Turner Biosystems). The three fractions were measured separately and total protein content is the sum of all three fractions.

### Phytic acid analysis of common bean grains

Samples for phytic acid analysis were prepared using modified protocols of Harland *et al*.^84^ and Ellis *et al*.^85^. We used the same milled samples as those for ICP-MS. In brief, 50 mg aliquots of powder were thoroughly mixed with 2 ml 2.4% HCl, incubated at room temperature (RT) for 1 hour followed by 3 min centrifugation at 13000 rpm (Thermo Scientific, Fresco17). 1.8 ml of supernatant was transferred into new 14 ml tubes and diluted with 8.2 ml Milli-Q water. To remove the inorganic phosphate, 10 ml diluted samples were applied to the 10 ml Poly-Prep Chromatography Columns (#7311550, Bio-Rad), pre-packed with 0.3 g of AG1-X4 resin, 100–200 mesh (AG 1-X4 Resin #1401341, Bio-Rad) and equilibrated with 0.7 M NaCl, followed by elution with 10 ml 0.1 M NaCl. Phytate was eluted from the columns with 0.7 M NaCl into 15 ml Falcon tubes. Each sample was analysed in triplicate to measure phytate with WADE reagent (0.03% FeCl3*6H2O and 0.3% sulfosalicylic acid in Milli-Q water, according to Latta *et al*.^86^).

### Data preparation and statistical analysis

All field trial data points, yield data and nutrient data were organized on Microsoft Excel (Microsoft, WA, USA) and all statistical analysis was performed on SPSS Statistics (version 21) (IBM, NY, USA). A detailed output of all statistical tests employed in this experiment are outlined in Supplementary Results File.

## Electronic supplementary material

Supplementary Figures

Supplementary Information

Supplementary Dataset
