# Supplementary material for: Reduction in nutritional quality and growing area suitability of common bean under climate change induced drought stress in Africa
Source: Sci Rep. 2018 Nov 1;8:16187. doi: 10.1038/s41598-018-33952-4 (PMC6212502; doi:10.1038/s41598-018-33952-4)
Supplement: Supplementary file 1 — Supplementary Figures [file 41598_2018_33952_MOESM1_ESM.pdf]

Supplementary Figures

Reduction in nutritional quality and growing area suitability of common bean under climate change induced drought stress in Africa

Marijke Hummel[1], Brendan F. Hallahan[1], Galina Brychkova[1], Julian Ramirez-Villegas[2], Veronica Guwela[1], Bartholomew Chataika[3], Edna Curley[1], Peter C. McKeown[1], Liam Morrison[1], Elise Talsma[2], Steve Beebe[2], Andy Jarvis[2], Rowland Chirwa[3], and Charles Spillane[1,*]

Supplementary Figure Legends

Supplementary Figure 1 | Rainfall and temperature data during crop growing period across rainfed (Season 1 and 3) and drought (Season 2 and 4) conditions. Source: Chitedze weather station.

Supplementary Figure 2 | Present-day drought season weather conditions mimics future bean growing season weather conditions. Using MarkSim standalone V.2 software two weather scenarios were simulated in Malawi for the year 2095. Under RCP 8.5 the months of March, April and May (bean growing season) will experience an average rainfall and temperature similar to that recorded in our field trials. (a) Ensemble of 7 of the most highly cited (>400 citations) General Circulation Models (GCMs). (b) Ensemble of all 17 GCMs.

Supplementary Figure 3 | Box and whisker plot showing average grain yield of 20 common bean varieties grown under rainfed and drought-stress conditions. 'X' indicates mean value.
*** $P < 0.001$

Supplementary Figure 4 | Percentage grain (A) Boron, (B) Sodium, (C) Magnesium, (D) Phosphorus, (E) Sulphur, (F) Potassium, (G) Calcium, (H) Titanium, (I) Chromium, (J) Manganese, (K) Copper, (L) Arsenic, (M) Rubidium, (N) Strontium, (O) Molybdenum, (P) Cadmium, (Q) Cobalt, (R) Nickel, (S) ^{57}Iron, (T) lead, (U) ^{56}Iron, (V) zinc, (W) phytic acid and protein (X) concentration change of each common bean variety under drought conditions relative to rainfed conditions.

Supplementary Figure 5 | Scatterplot comparing combinations of Fe and Zn concentrations in the CIAT core collection of 1000 cultivated common bean accessions (black circles) with equivalent values for the twenty common bean varieties tested in this study under rainfed (green circles) or drought conditions (red triangles).

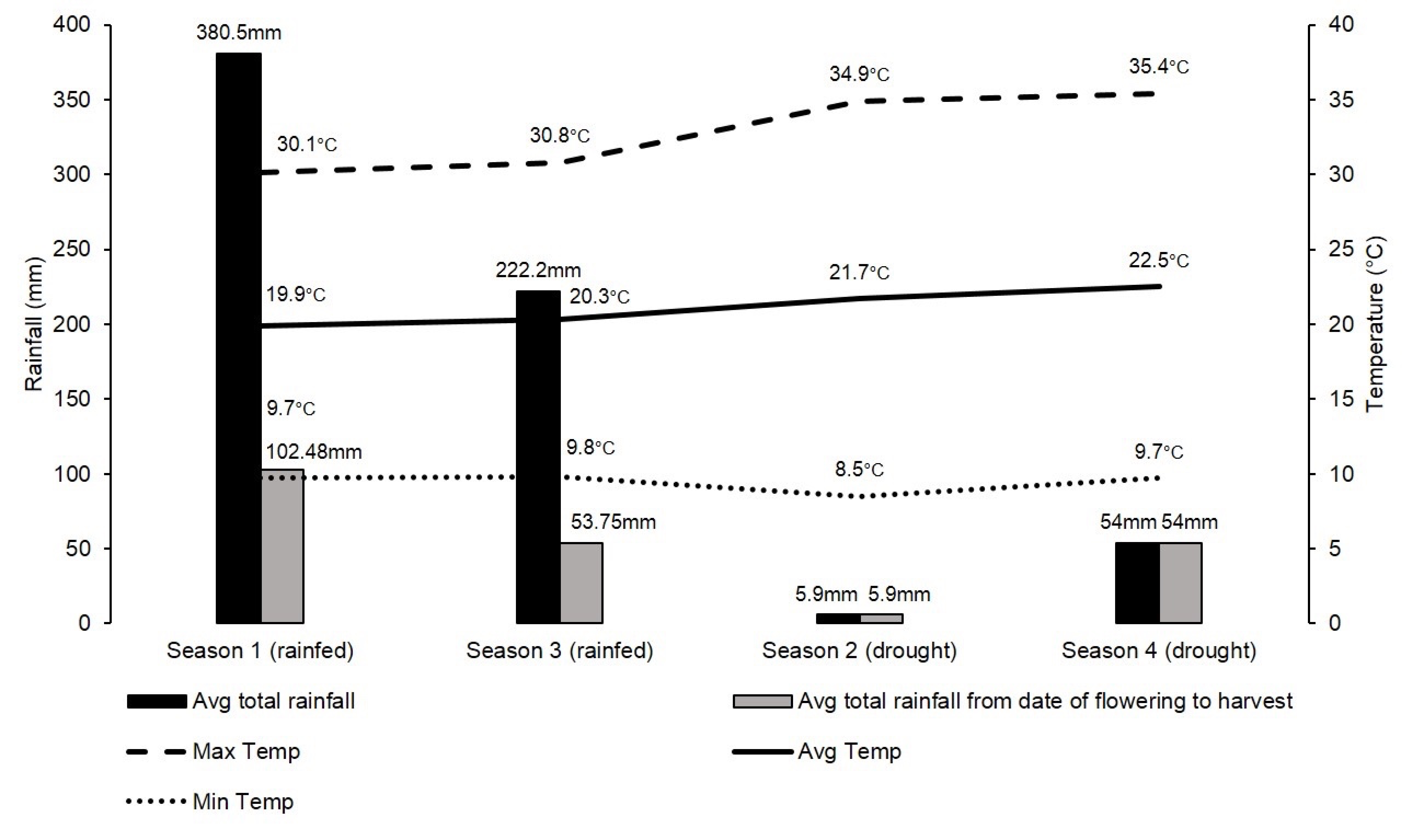

Rainfall (mm)
Temperature (°C)
400
350
300
250
200
150
100
50
0
40
35
30
25
20
15
10
5
0
380.5mm
222.2mm
102.48mm
53.75mm
5.9mm
5.9mm
54mm
54mm
30.1°C
30.8°C
34.9°C
35.4°C
19.9°C
20.3°C
21.7°C
22.5°C
9.7°C
9.8°C
8.5°C
9.7°C
Season 1 (rainfed)
Season 3 (rainfed)
Season 2 (drought)
Season 4 (drought)
Avg total rainfall
Avg total rainfall from date of flowering to harvest
Max Temp
Avg Temp
Min Temp

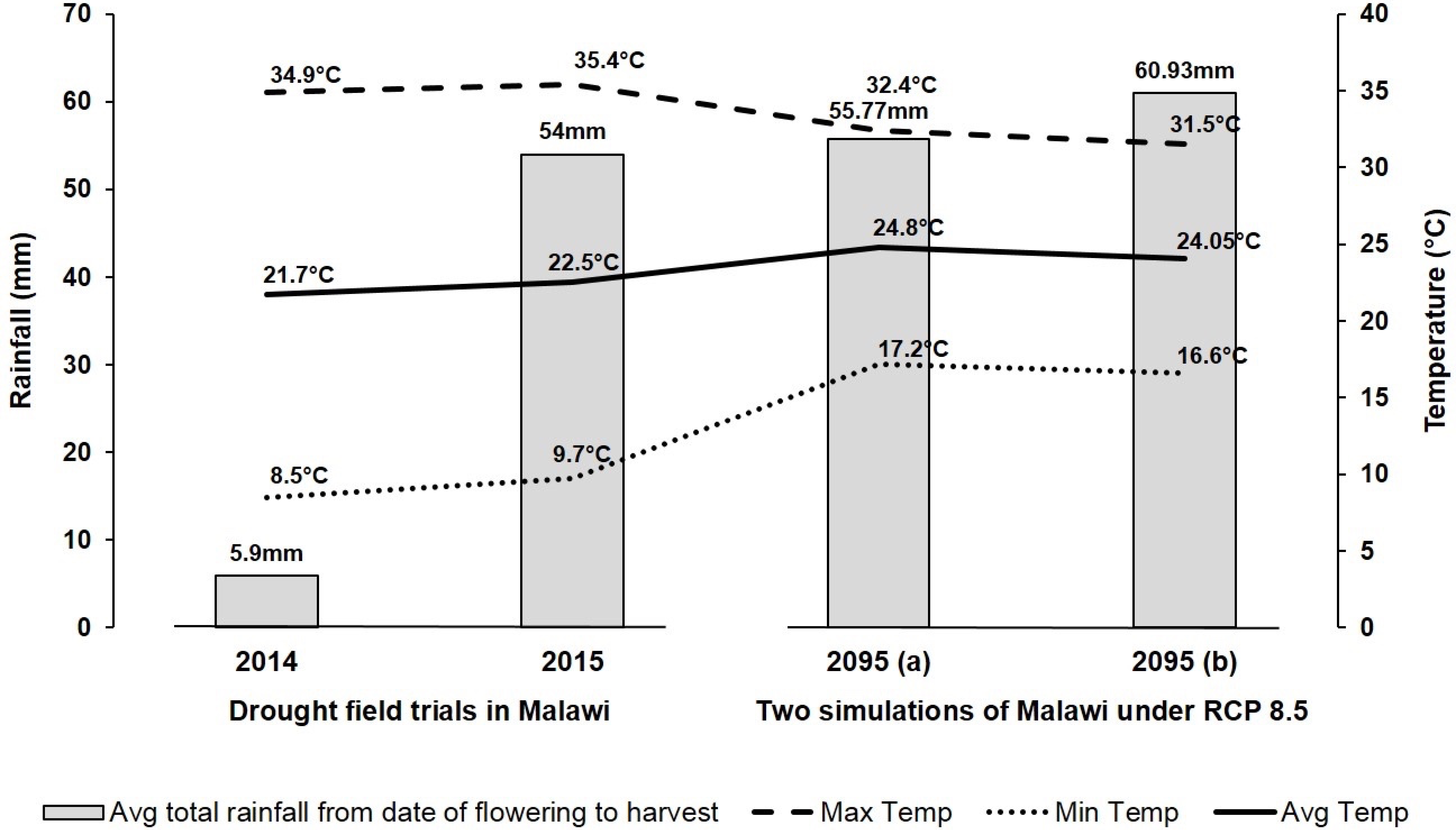

Rainfall (mm)
Temperature (°C)
70
60
50
40
30
20
10
0
40
35
30
25
20
15
10
5
0
34.9°C
35.4°C
32.4°C
31.5°C
60.93mm
55.77mm
54mm
5.9mm
24.8°C
24.05°C
21.7°C
22.5°C
17.2°C
16.6°C
9.7°C
8.5°C
2014
2015
2095 (a)
2095 (b)
Drought field trials in Malawi
Two simulations of Malawi under RCP 8.5
Avg total rainfall from date of flowering to harvest
Max Temp
Min Temp
Avg Temp

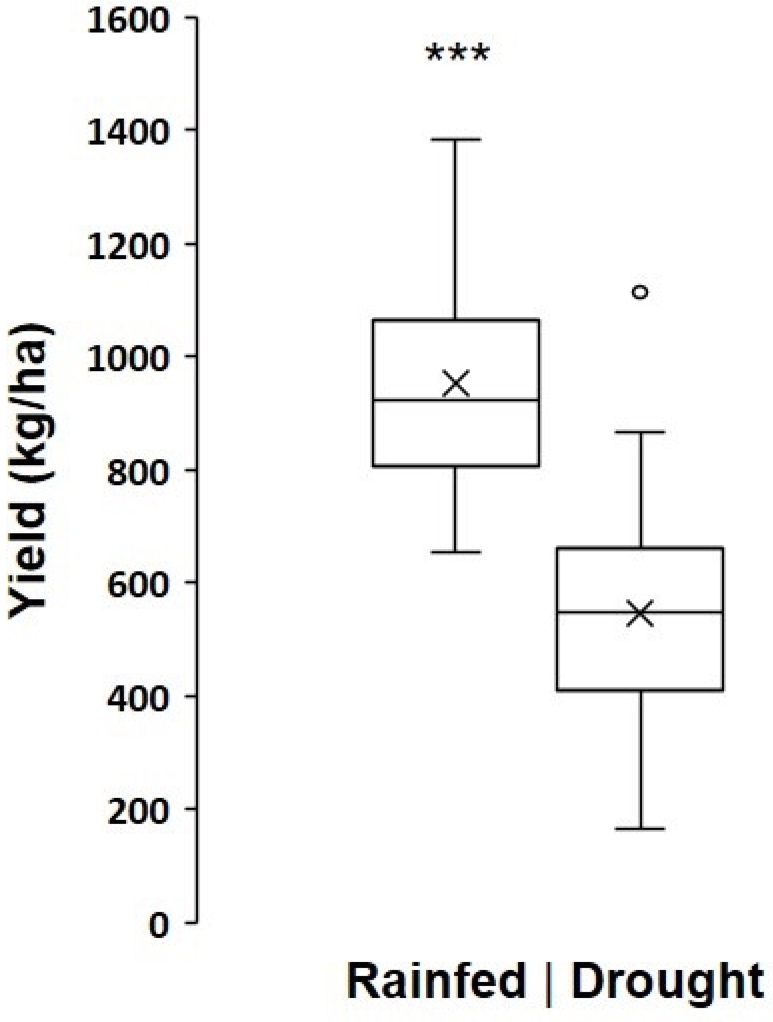

1600
1400
1200
1000
800
600
400
200
0
Yield (kg/ha)
***
Rainfed | Drought

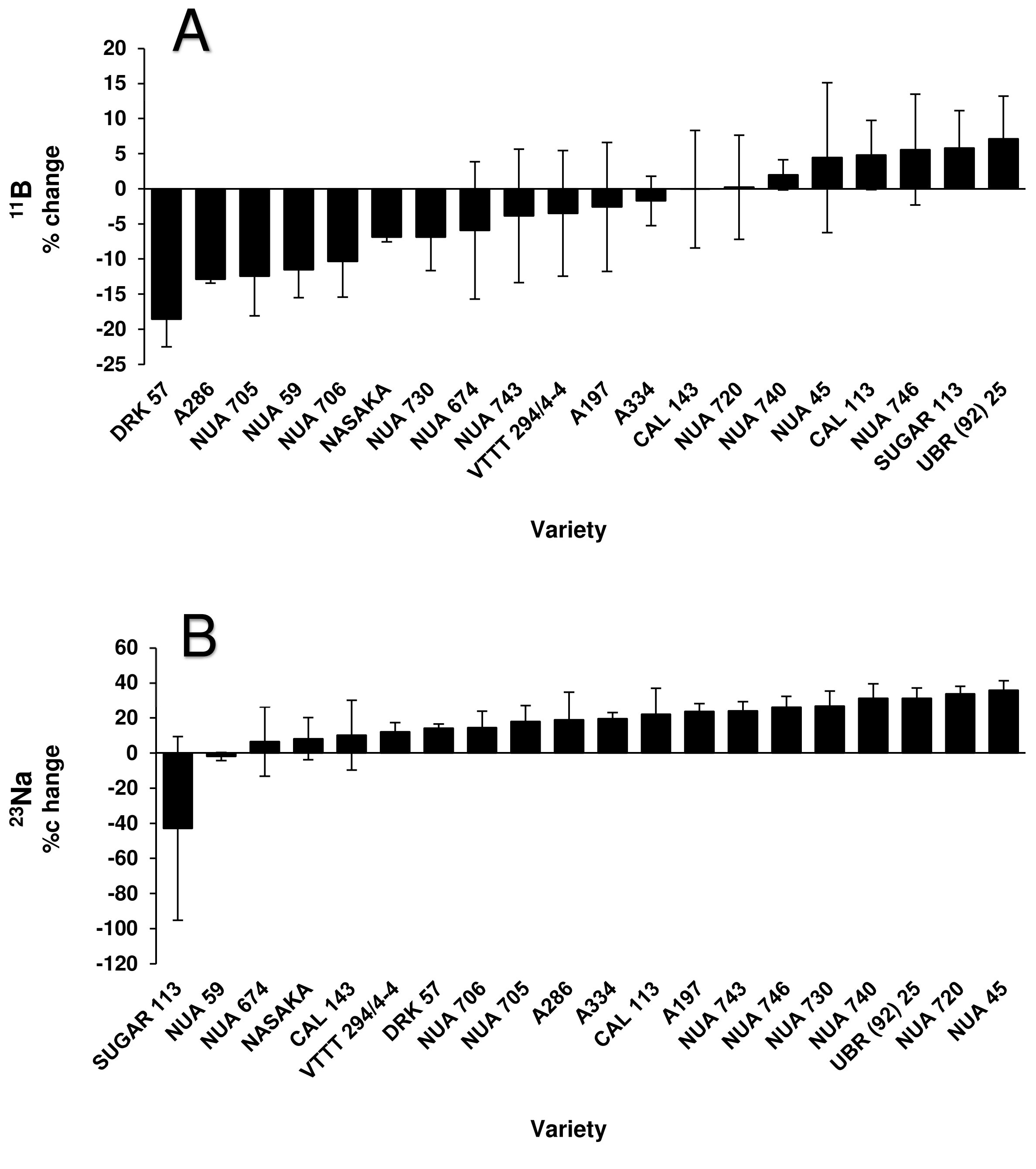

A
20
15
10
5
0
-5
-10
-15
-20
-25
^{11}B
% change
DRK 57
A286
NUA 705
NUA 59
NUA 706
NASAKA
NUA 730
NUA 674
NUA 743
VTTT 294/4-4
A197
A334
CAL 143
NUA 720
NUA 740
NUA 45
CAL 113
NUA 746
SUGAR 113
UBR (92) 25
Variety
B
60
40
20
0
-20
-40
-60
-80
-100
-120
^{23}Na
%c hange
SUGAR 113
NUA 59
NUA 674
NASAKA
CAL 143
VTTT 294/4-4
DRK 57
NUA 706
NUA 705
A286
A334
CAL 113
A197
NUA 743
NUA 746
NUA 730
NUA 740
UBR (92) 25
NUA 720
NUA 45
Variety

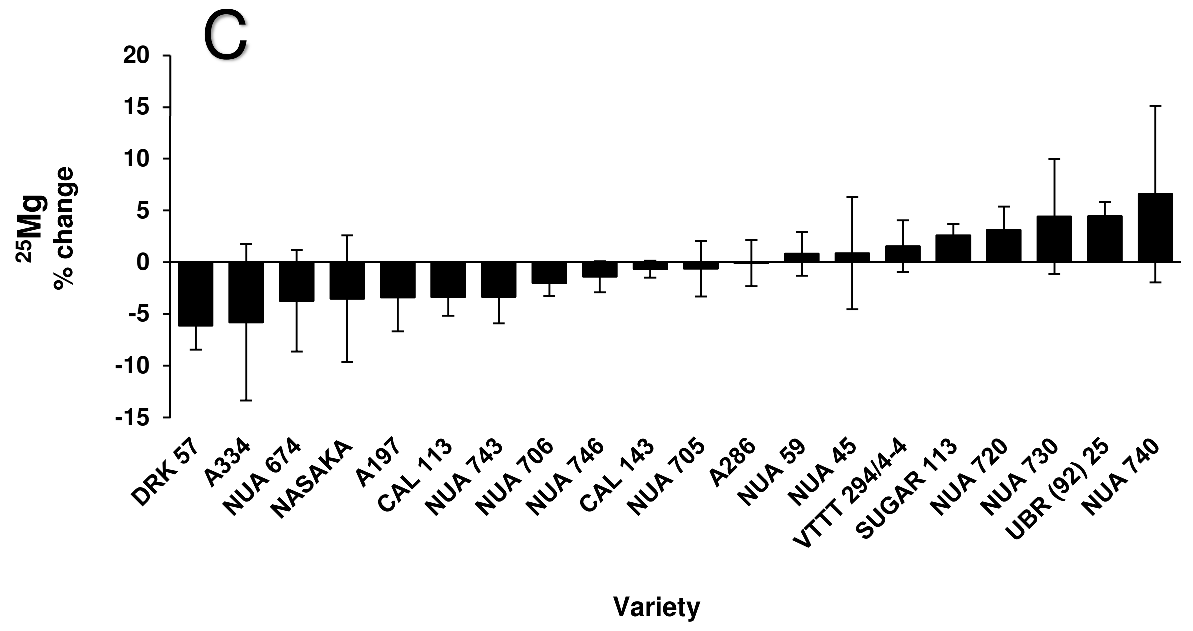

C
25Mg
% change
20
15
10
5
0
-5
-10
-15
DRK 57
A334
NUA 674
NASAKA
A197
CAL 113
NUA 743
NUA 706
NUA 746
CAL 143
NUA 705
A286
NUA 59
NUA 45
VTTT 294/4-4
SUGAR 113
NUA 720
NUA 730
UBR (92) 25
NUA 740
Variety

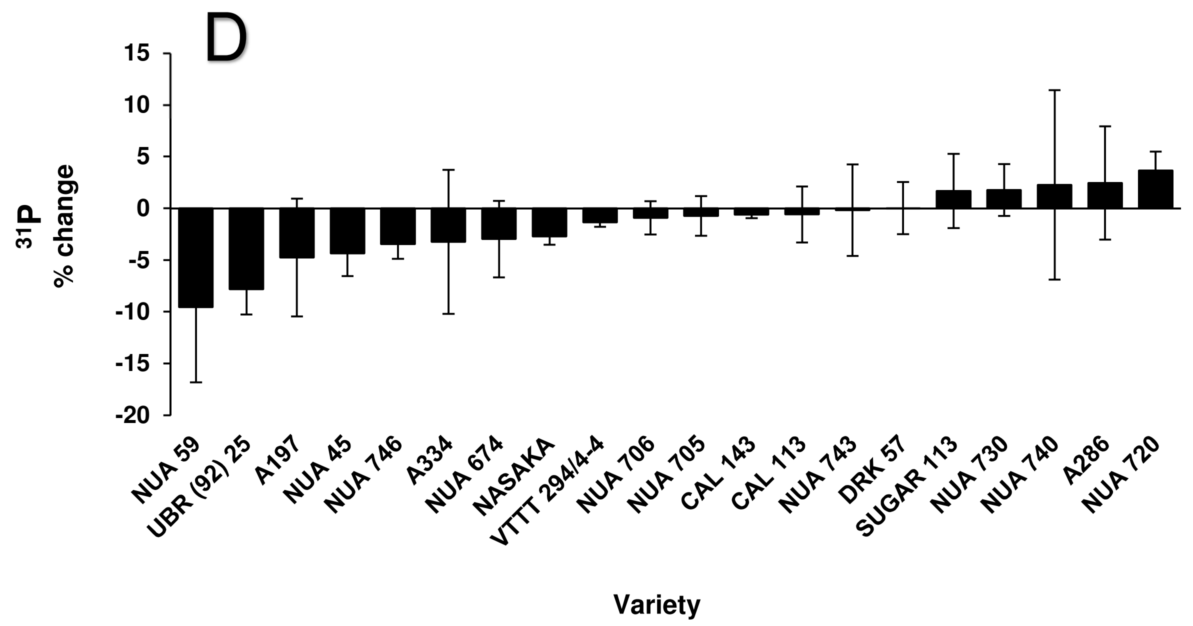

D
31P
% change
15
10
5
0
-5
-10
-15
-20
NUA 59
UBR (92) 25
A197
NUA 45
NUA 746
A334
NUA 674
NASAKA
VTTT 294/4-4
NUA 706
NUA 705
CAL 143
CAL 113
NUA 743
DRK 57
SUGAR 113
NUA 730
NUA 740
A286
NUA 720
Variety

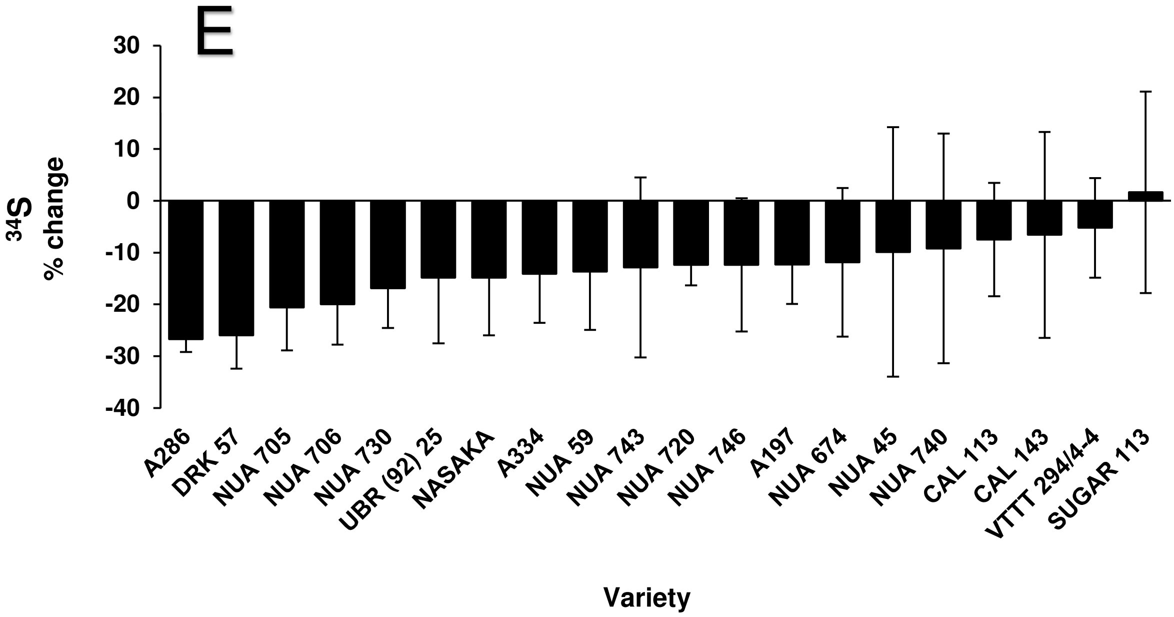
E
30
20
10
0
-10
-20
-30
-40
^{34}S
% change
A286
DRK 57
NUA 705
NUA 706
NUA 730
UBR (92) 25
NASAKA
A334
NUA 59
NUA 743
NUA 720
NUA 746
A197
NUA 674
NUA 45
NUA 740
CAL 113
CAL 143
VTTT 294/4-4
SUGAR 113
Variety

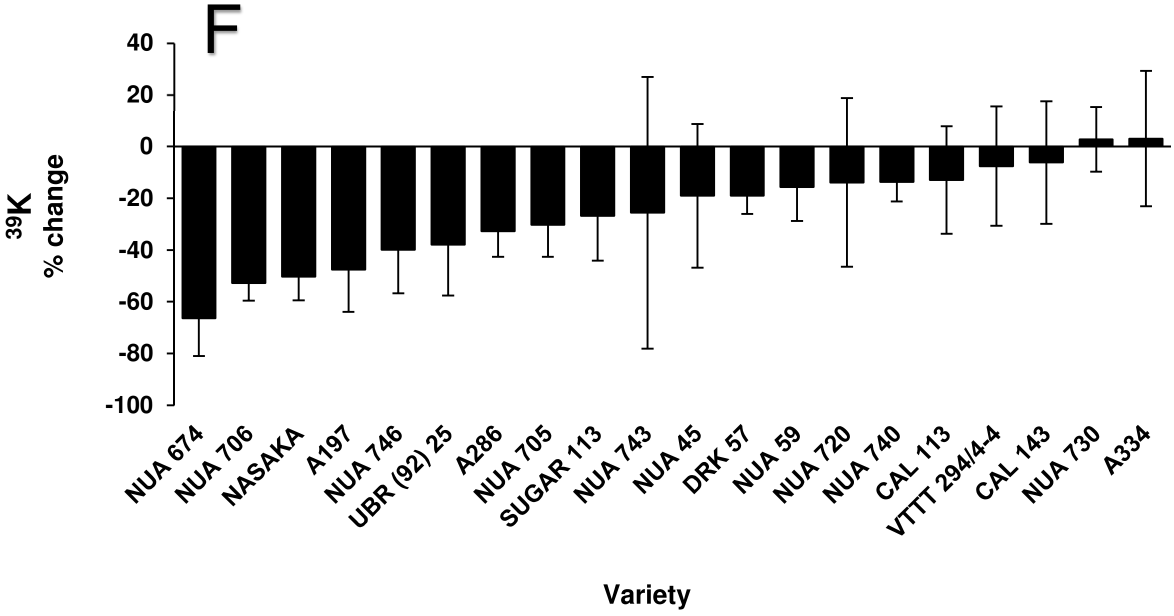
F
40
20
0
-20
-40
-60
-80
-100
^{39}K
% change
NUA 674
NUA 706
NASAKA
A197
NUA 746
UBR (92) 25
A286
NUA 705
SUGAR 113
NUA 743
NUA 45
DRK 57
NUA 59
NUA 720
NUA 740
CAL 113
VTTT 294/4-4
CAL 143
NUA 730
A334
Variety

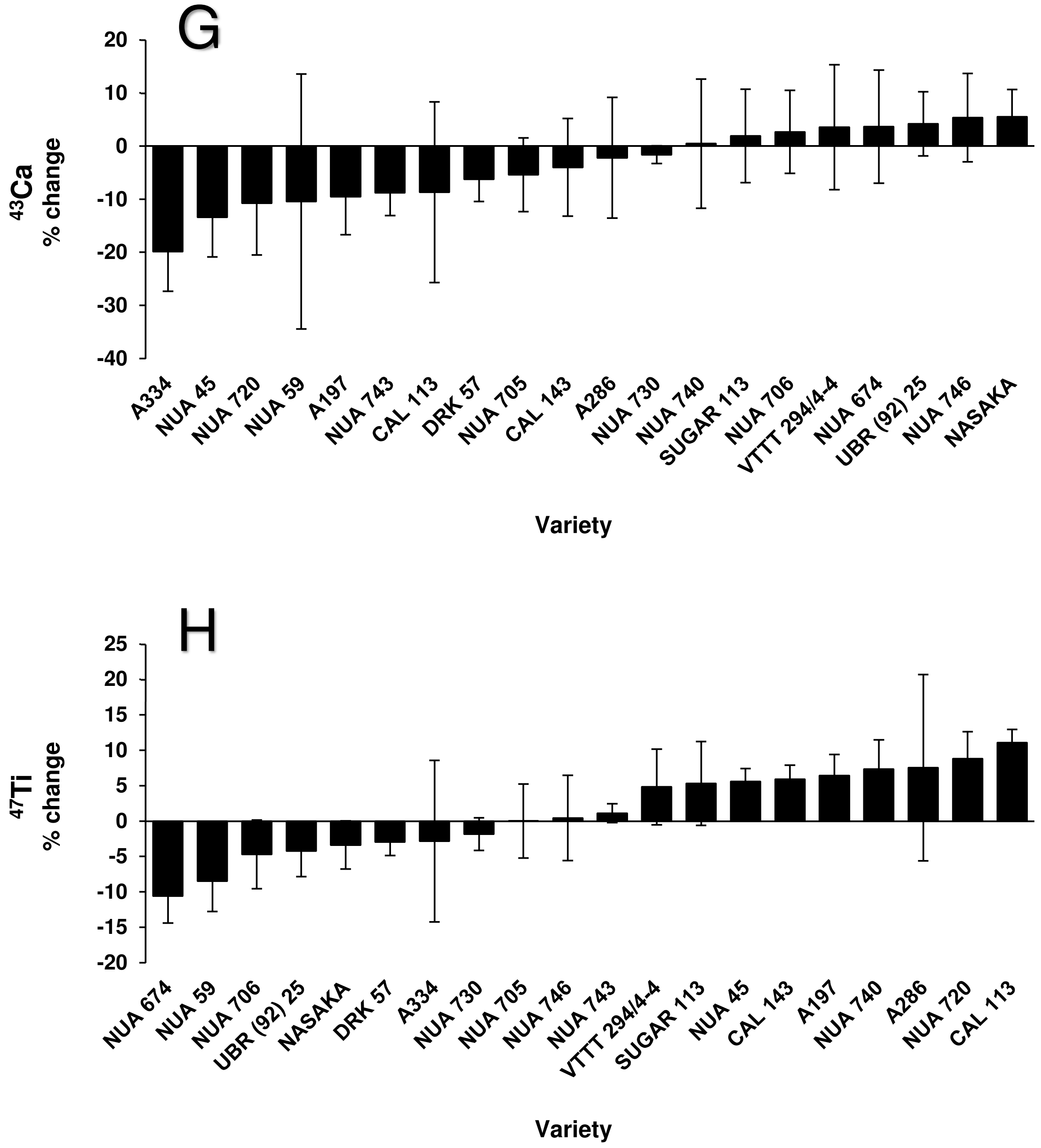
G
43Ca
% change
20
10
0
-10
-20
-30
-40
A334
NUA 45
NUA 720
NUA 59
A197
NUA 743
CAL 113
DRK 57
NUA 705
CAL 143
A286
NUA 730
NUA 740
SUGAR 113
NUA 706
VTTT 294/4-4
NUA 674
UBR (92) 25
NUA 746
NASAKA
Variety
H
47Ti
% change
25
20
15
10
5
0
-5
-10
-15
-20
NUA 674
NUA 59
NUA 706
UBR (92) 25
NASAKA
DRK 57
A334
NUA 730
NUA 705
NUA 746
NUA 743
VTTT 294/4-4
SUGAR 113
NUA 45
CAL 143
A197
NUA 740
A286
NUA 720
CAL 113
Variety

I

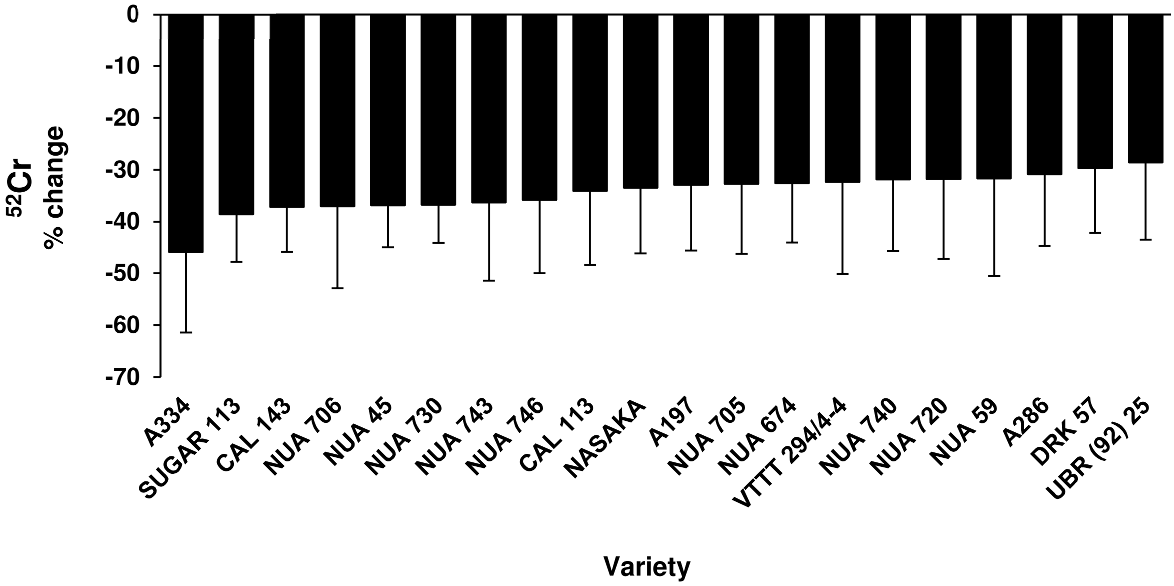
0
-10
-20
-30
-40
-50
-60
-70
^{52}Cr
% change
A334
SUGAR 113
CAL 143
NUA 706
NUA 45
NUA 730
NUA 743
NUA 746
CAL 113
NASAKA
A197
NUA 705
NUA 674
VTTT 294/4-4
NUA 740
NUA 720
NUA 59
A286
DRK 57
UBR (92) 25
Variety

J

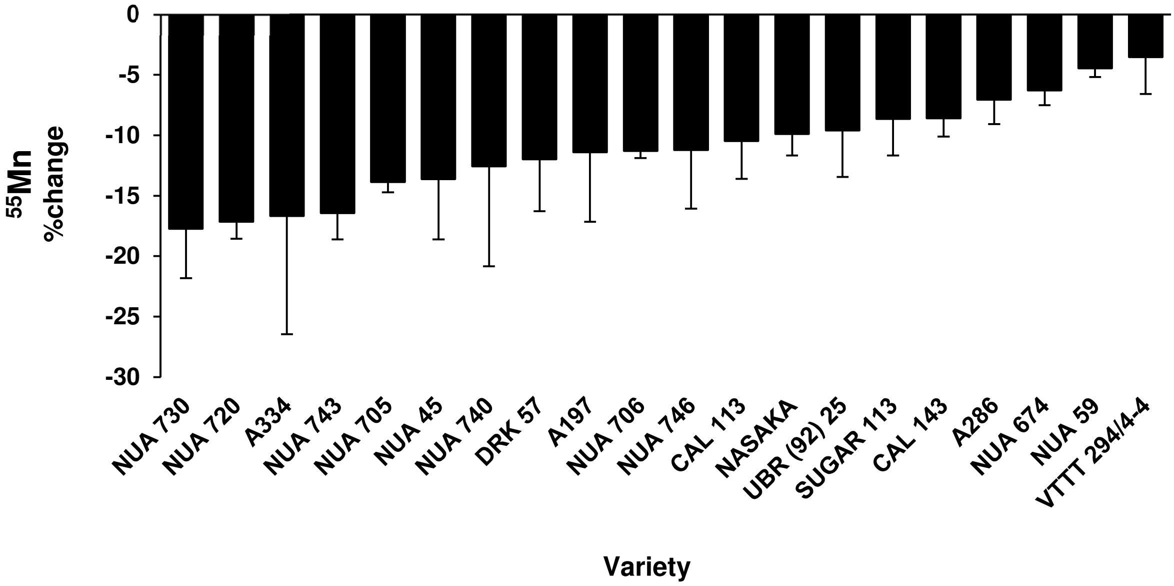
0
-5
-10
-15
-20
-25
-30
^{55}Mn
%change
NUA 730
NUA 720
A334
NUA 743
NUA 705
NUA 45
NUA 740
DRK 57
A197
NUA 706
NUA 746
CAL 113
NASAKA
UBR (92) 25
SUGAR 113
CAL 143
A286
NUA 674
NUA 59
VTTT 294/4-4
Variety

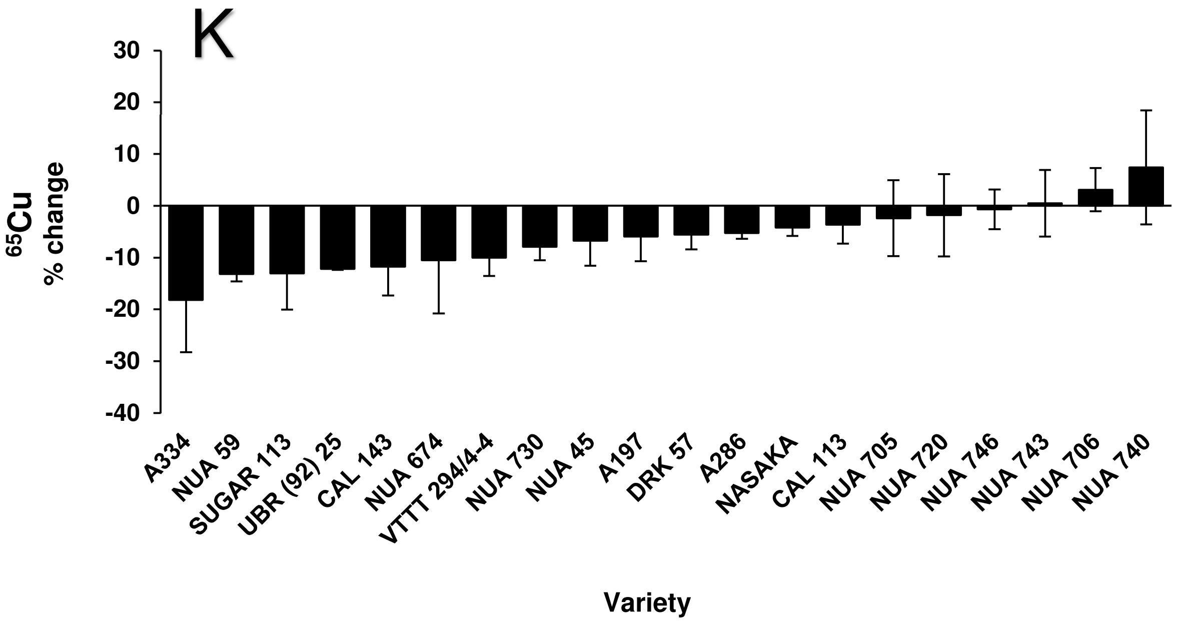
K
30
20
10
0
-10
-20
-30
-40
^{65}Cu
% change
A334
NUA 59
SUGAR 113
UBR (92) 25
CAL 143
NUA 674
VTTT 294/4-4
NUA 730
NUA 45
A197
DRK 57
A286
NASAKA
CAL 113
NUA 705
NUA 720
NUA 746
NUA 743
NUA 706
NUA 740
Variety

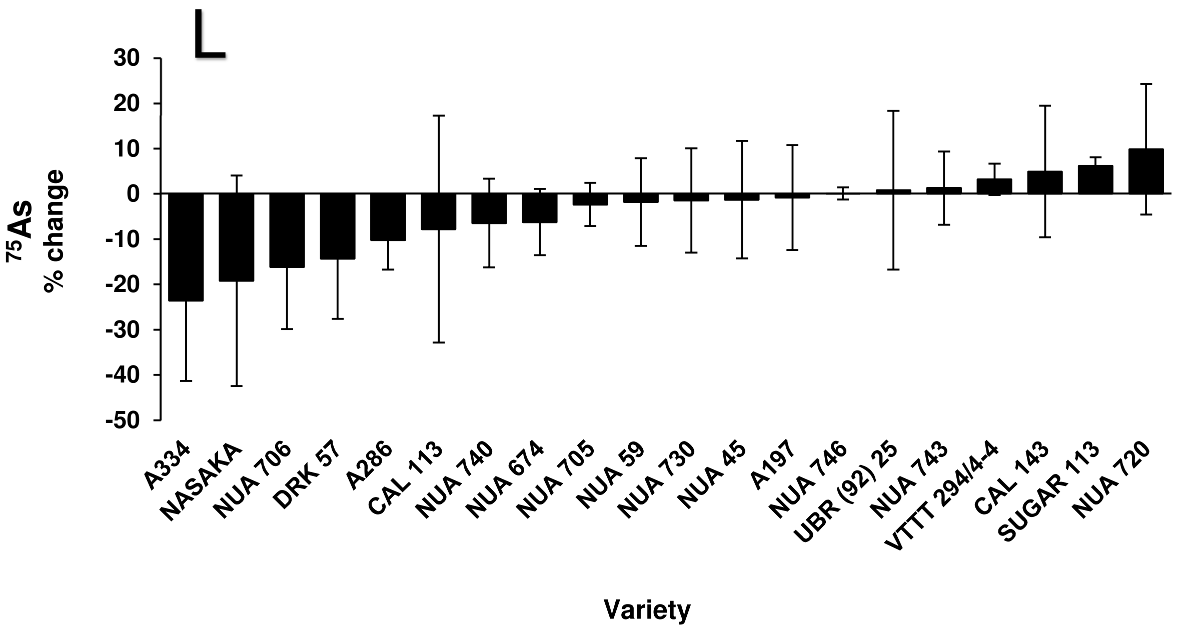
L
30
20
10
0
-10
-20
-30
-40
-50
^{75}As
% change
A334
NASAKA
NUA 706
DRK 57
A286
CAL 113
NUA 740
NUA 674
NUA 705
NUA 59
NUA 730
NUA 45
A197
NUA 746
UBR (92) 25
NUA 743
VTTT 294/4-4
CAL 143
SUGAR 113
NUA 720
Variety

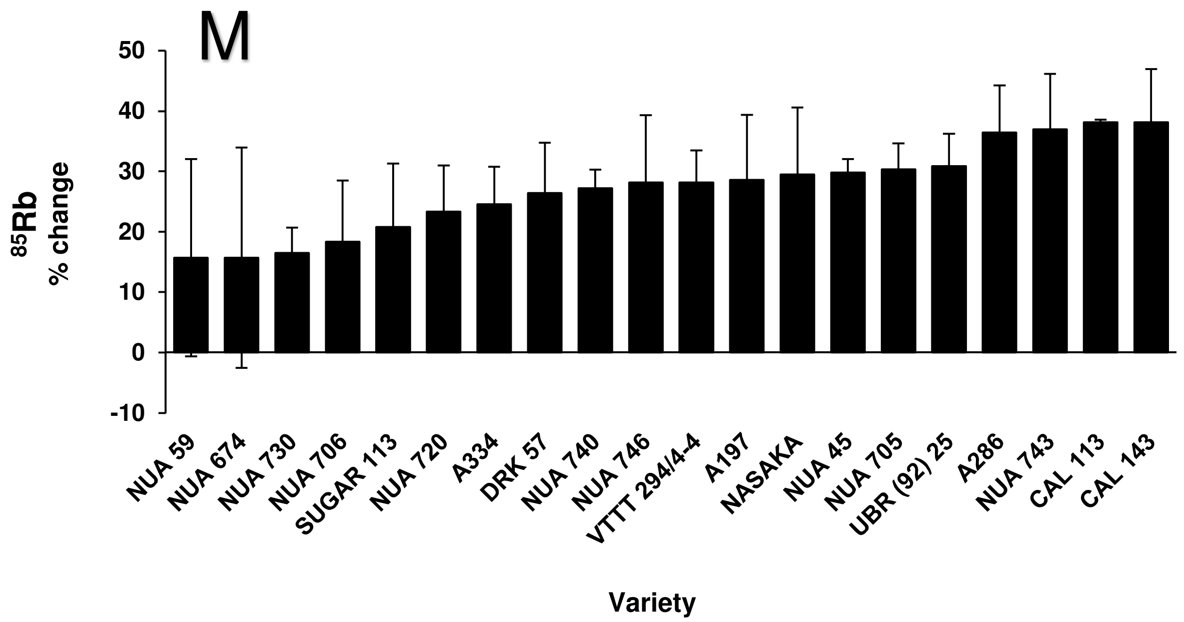
M
85Rb
% change
50
40
30
20
10
0
-10
NUA 59
NUA 674
NUA 730
NUA 706
SUGAR 113
NUA 720
A334
DRK 57
NUA 740
NUA 746
VTTT 294/4-4
A197
NASAKA
NUA 45
NUA 705
UBR (92) 25
A286
NUA 743
CAL 113
CAL 143
Variety

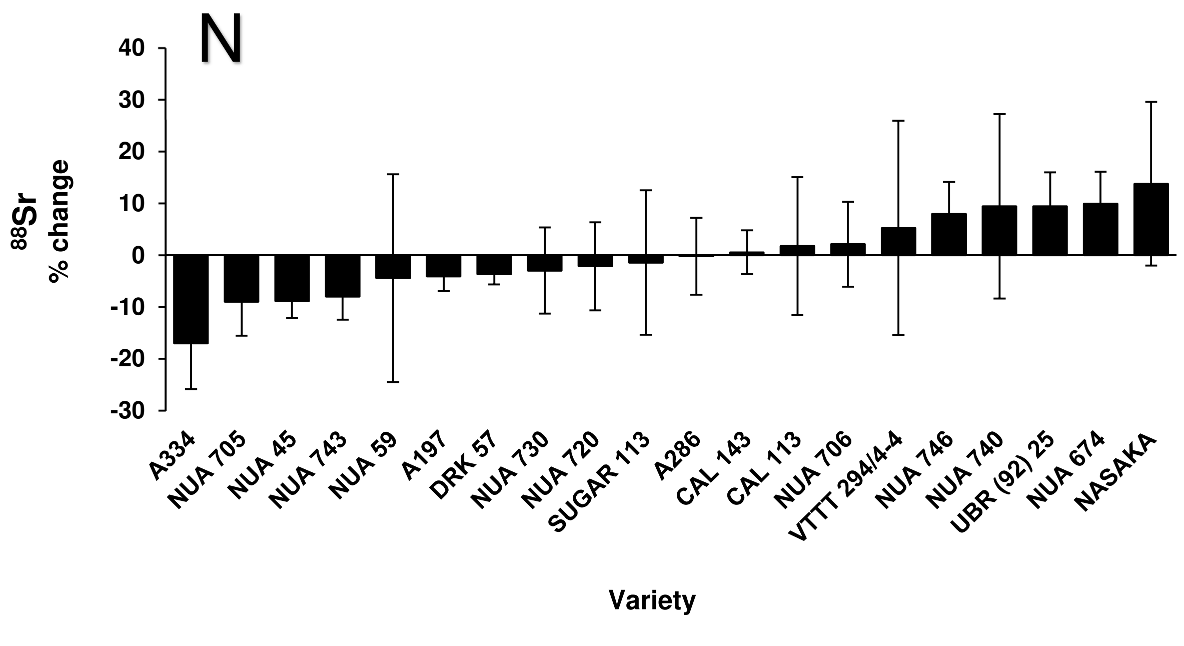
N
88Sr
% change
40
30
20
10
0
-10
-20
-30
A334
NUA 705
NUA 45
NUA 743
NUA 59
A197
DRK 57
NUA 730
NUA 720
SUGAR 113
A286
CAL 143
CAL 113
NUA 706
VTTT 294/4-4
NUA 746
NUA 740
UBR (92) 25
NUA 674
NASAKA
Variety

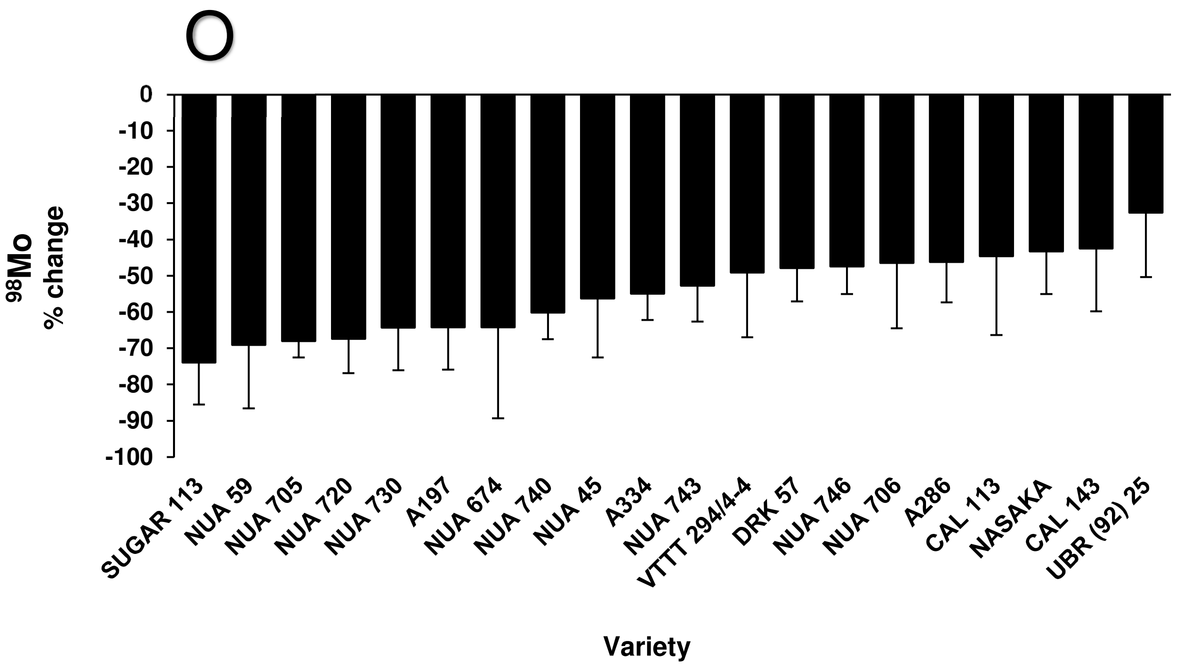
O
0
-10
-20
-30
-40
-50
-60
-70
-80
-90
-100
^{98}Mo
% change
SUGAR 113
NUA 59
NUA 705
NUA 720
NUA 730
A197
NUA 674
NUA 740
NUA 45
A334
NUA 743
VTTT 294/4-4
DRK 57
NUA 746
NUA 706
A286
CAL 113
NASAKA
CAL 143
UBR (92) 25
Variety

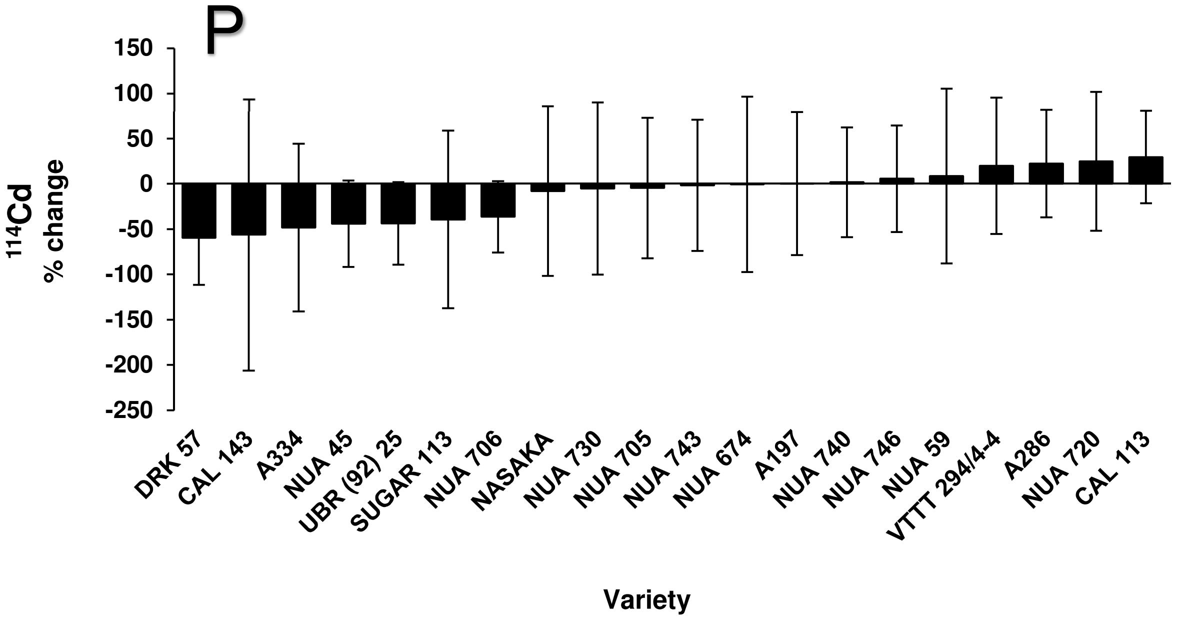
P
150
100
50
0
-50
-100
-150
-200
-250
^{114}Cd
% change
DRK 57
CAL 143
A334
NUA 45
UBR (92) 25
SUGAR 113
NUA 706
NASAKA
NUA 730
NUA 705
NUA 743
NUA 674
A197
NUA 740
NUA 746
NUA 59
VTTT 294/4-4
A286
NUA 720
CAL 113
Variety

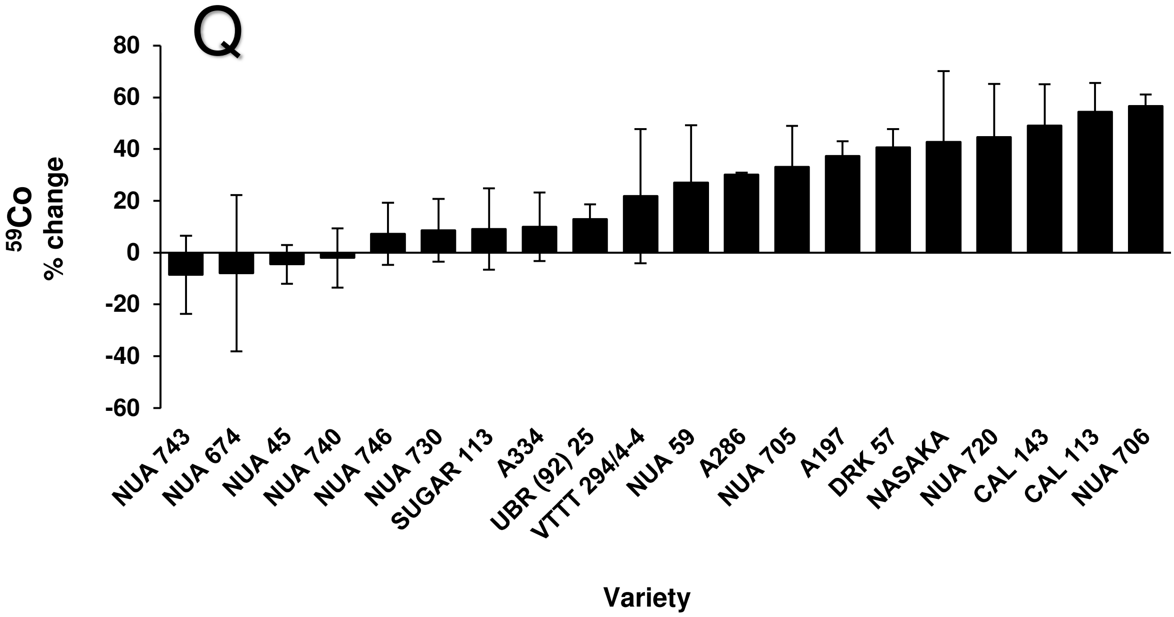
Q
^{59}Co
% change
80
60
40
20
0
-20
-40
-60
NUA 743
NUA 674
NUA 45
NUA 740
NUA 746
NUA 730
SUGAR 113
A334
UBR (92) 25
VTTT 294/4-4
NUA 59
A286
NUA 705
A197
DRK 57
NASAKA
NUA 720
CAL 143
CAL 113
NUA 706
Variety

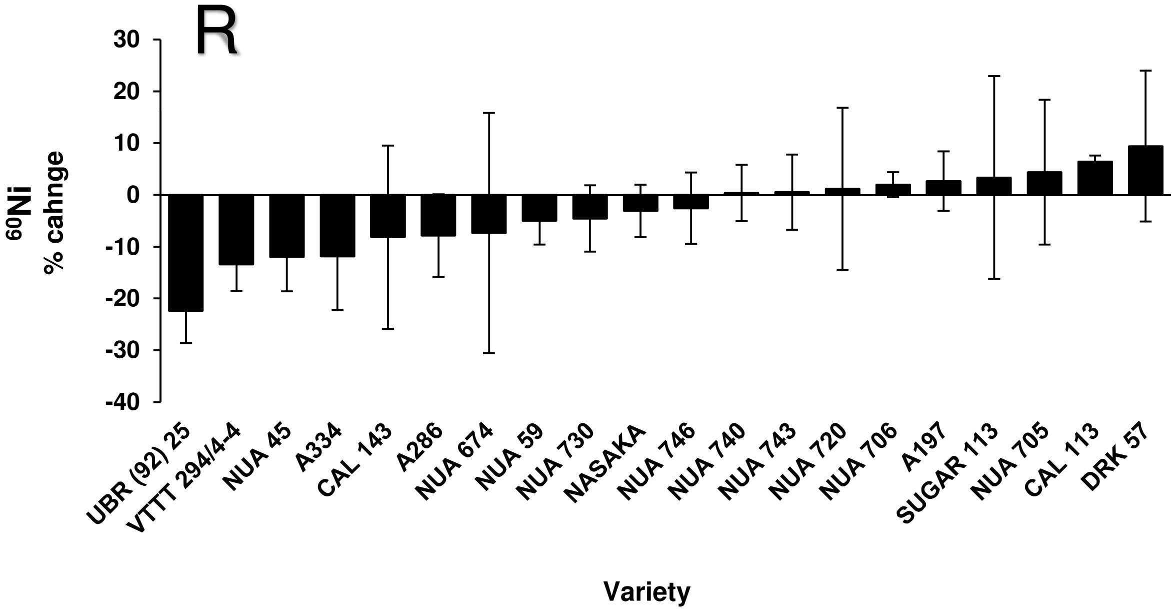
R
^{60}Ni
% cahnge
30
20
10
0
-10
-20
-30
-40
UBR (92) 25
VTTT 294/4-4
NUA 45
A334
CAL 143
A286
NUA 674
NUA 59
NUA 730
NASAKA
NUA 746
NUA 740
NUA 743
NUA 720
NUA 706
A197
SUGAR 113
NUA 705
CAL 113
DRK 57
Variety

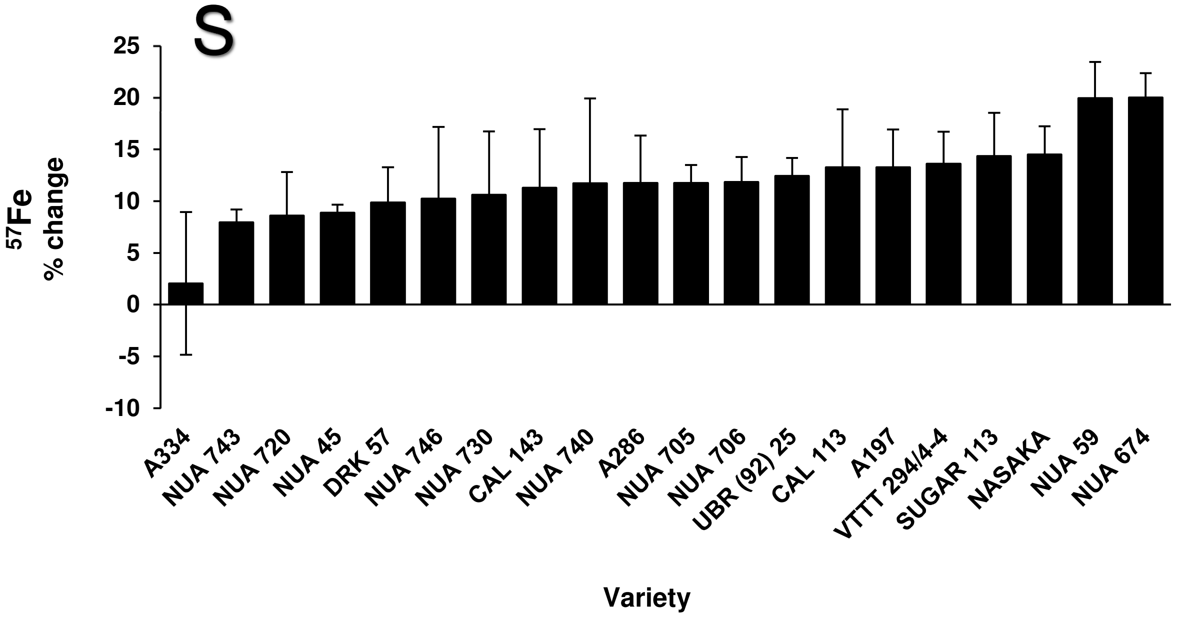
S
25
20
15
10
5
0
-5
-10
57Fe
% change
A334
NUA 743
NUA 720
NUA 45
DRK 57
NUA 746
NUA 730
CAL 143
NUA 740
A286
NUA 705
NUA 706
UBR (92) 25
CAL 113
A197
VTTT 294/4-4
SUGAR 113
NASAKA
NUA 59
NUA 674
Variety

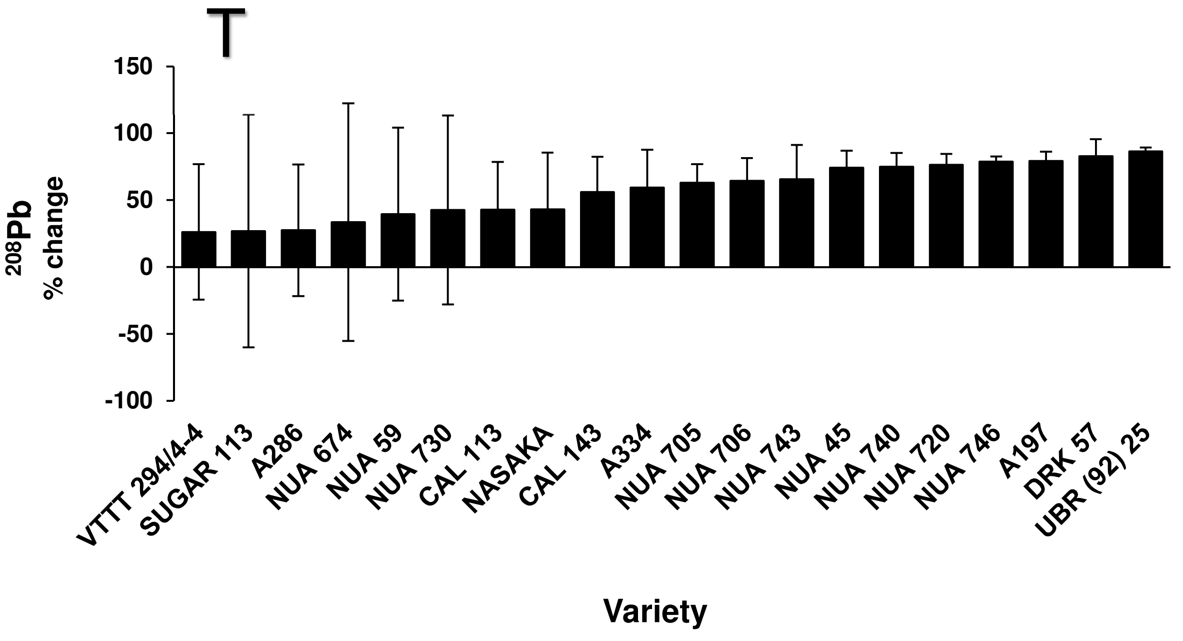
T
150
100
50
0
-50
-100
208Pb
% change
VTTT 294/4-4
SUGAR 113
A286
NUA 674
NUA 59
NUA 730
CAL 113
NASAKA
CAL 143
A334
NUA 705
NUA 706
NUA 743
NUA 45
NUA 740
NUA 720
NUA 746
A197
DRK 57
UBR (92) 25
Variety

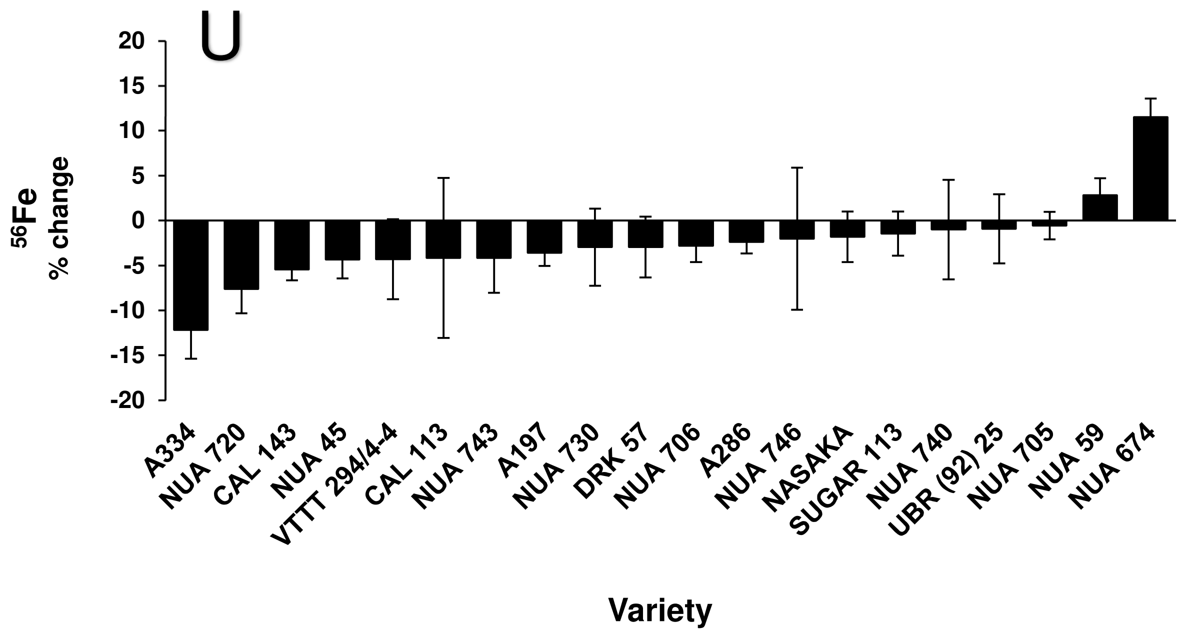
U
20
15
10
5
0
-5
-10
-15
-20
56Fe
% change
A334
NUA 720
CAL 143
NUA 45
VTTT 294/4-4
CAL 113
NUA 743
A197
NUA 730
DRK 57
NUA 706
A286
NUA 746
NASAKA
SUGAR 113
NUA 740
UBR (92) 25
NUA 705
NUA 59
NUA 674
Variety

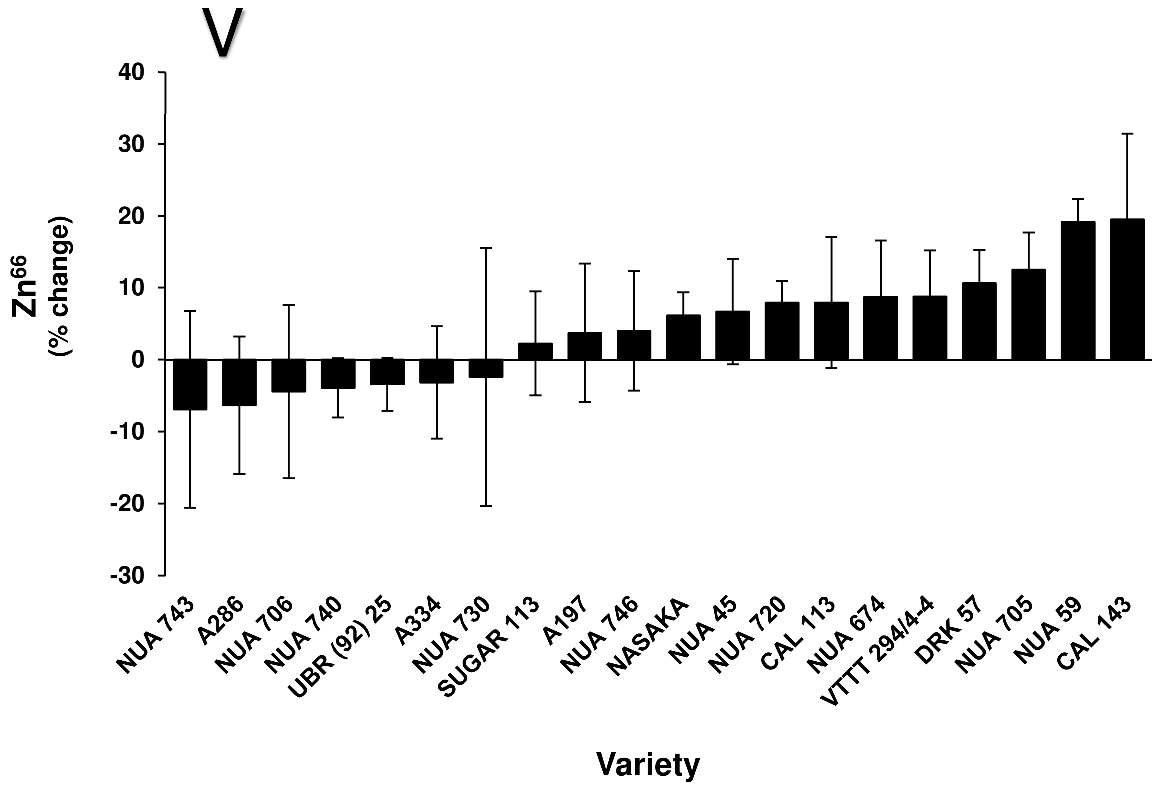
V
40
30
20
10
0
-10
-20
-30
Zn66
(% change)
NUA 743
A286
NUA 706
NUA 740
UBR (92) 25
A334
NUA 730
SUGAR 113
A197
NUA 746
NASAKA
NUA 45
NUA 720
CAL 113
NUA 674
VTTT 294/4-4
DRK 57
NUA 705
NUA 59
CAL 143
Variety

W

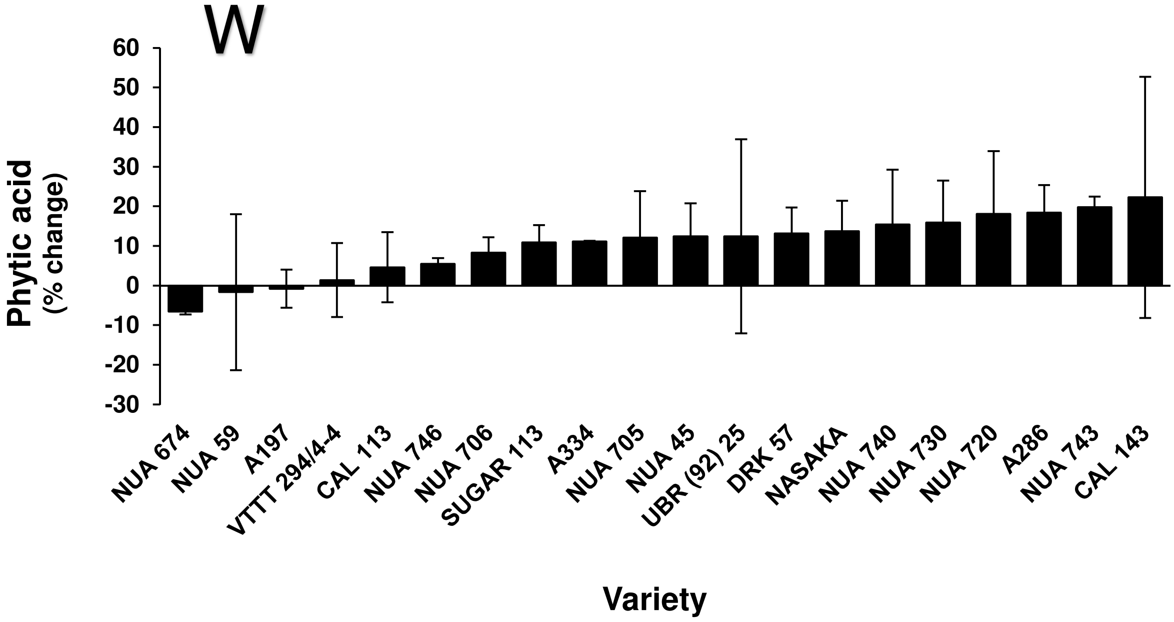

Phytic acid
(% change)
60
50
40
30
20
10
0
-10
-20
-30
NUA 674
NUA 59
A197
VTTT 294/4-4
CAL 113
NUA 746
NUA 706
SUGAR 113
A334
NUA 705
NUA 45
UBR (92) 25
DRK 57
NASAKA
NUA 740
NUA 730
NUA 720
A286
NUA 743
CAL 143
Variety

X

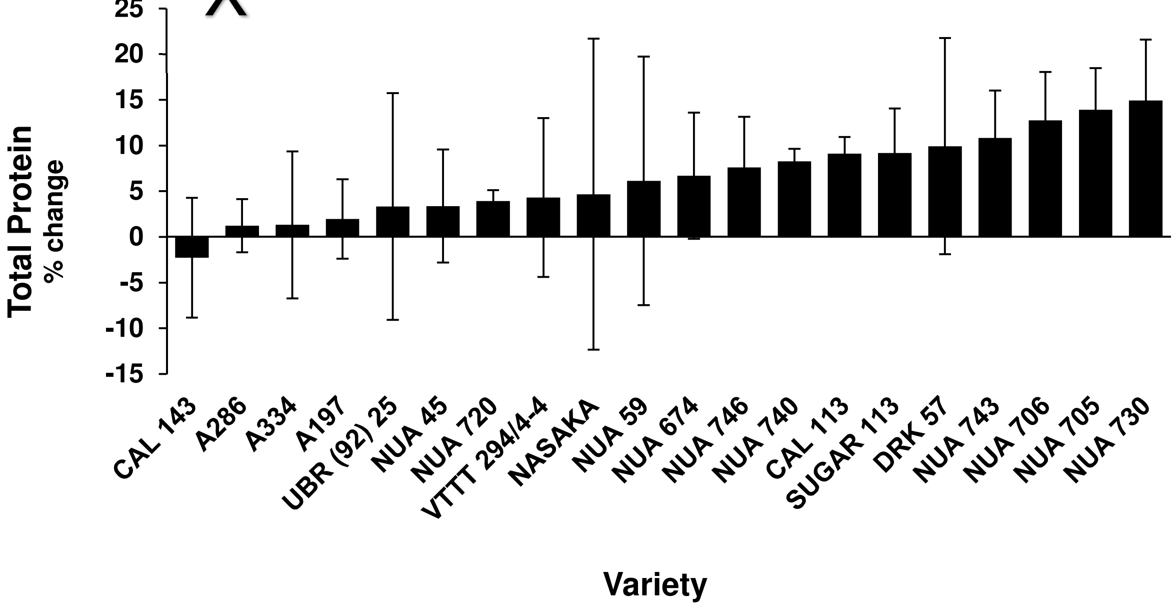

Total Protein
% change
25
20
15
10
5
0
-5
-10
-15
CAL 143
A286
A334
A197
UBR (92) 25
NUA 45
NUA 720
VTTT 294/4-4
NASAKA
NUA 59
NUA 674
NUA 746
NUA 740
CAL 113
SUGAR 113
DRK 57
NUA 743
NUA 706
NUA 705
NUA 730
Variety

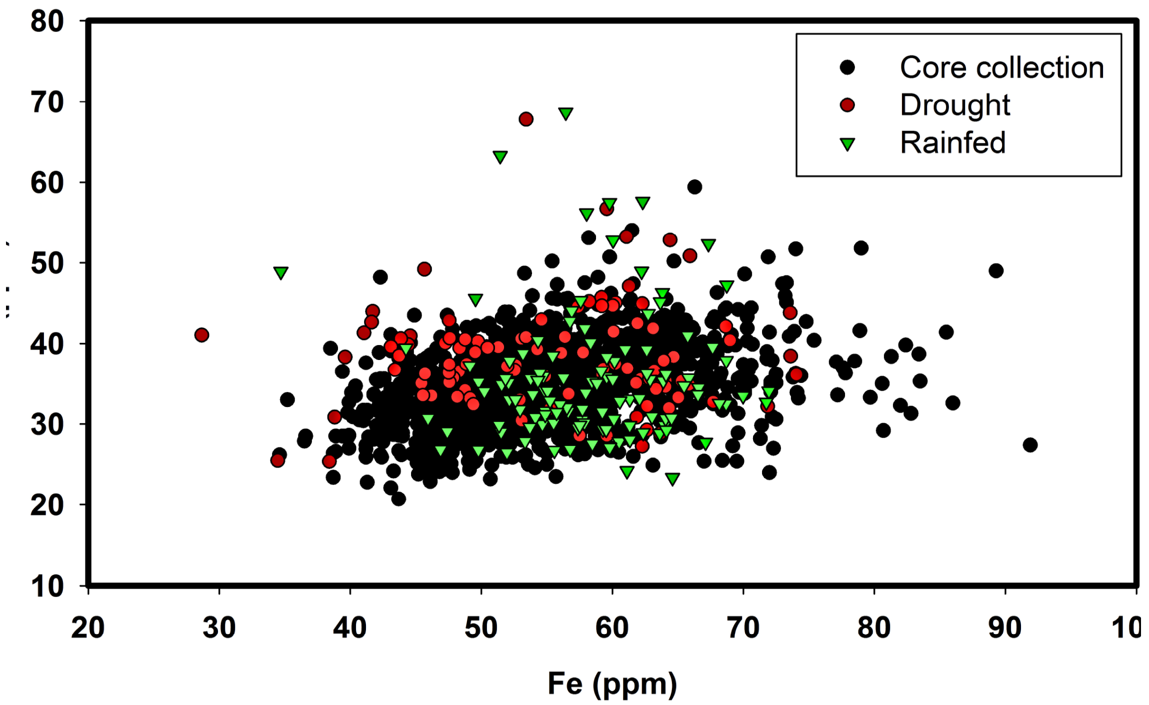
80
70
60
50
40
30
20
10
20
30
40
50
60
70
80
90
10
Fe (ppm)
Core collection
Drought
Rainfed
