# Supplementary material for: Reduction in nutritional quality and growing area suitability of common bean under climate change induced drought stress in Africa
Source: Sci Rep. 2018 Nov 1;8:16187. doi: 10.1038/s41598-018-33952-4 (PMC6212502; doi:10.1038/s41598-018-33952-4)
Supplement: Supplementary file 2 — Supplementary Information [file 41598_2018_33952_MOESM2_ESM.pdf]

Supplementary Data & Information

Reduction in nutritional quality and growing area suitability of common bean under climate change induced drought stress in Africa

Marijke Hummel[1], Brendan F. Hallahan[1], Galina Brychkova[1], Julian Ramirez-Villegas[2], Veronica Guwela[1], Bartholomew Chataika[3], Edna Curley[1], Peter C. McKeown[1], Liam Morrison[1], Elise Talsma[2], Steve Beebe[2], Andy Jarvis[2], Rowland Chirwa[3], and Charles Spillane[1,*]

Supplementary Information | Detailed ICP-MS methodology

Raw data file | Yield and micronutrient levels.

Supplementary Results File | SPSS statistical output of all analyses

Supplementary Information

Methods and Materials

ICP-MS analysis of elemental composition of common bean grains

Bean powder (ground/milled) in Eppendorf tubes were transferred into Pyrex test tubes (16 x 100 mm). After weighing the appropriate number of samples (these masses were used to calculate the rest of the sample masses, trace metal grade nitric acid (J. T. Baker Instra-Analyzed; Avantor Performance Materials; Scientific & Chemical Supplies Ltd, Aberdeen, UK) spiked with indium internal standard was added to the tubes (1.20 mL per tube); hydrogen peroxide (1.20 mL per tube) (Primar-Trace analysis grade, 30%; Fisher Scientific, Loughborough, UK) was also added. The beans seed samples were left overnight to pre-digest. They were then digested in dry block heaters (DigiPREP MS, SCP Science; QMX Laboratories, Essex, UK) at 115˚C for 4 hours. The digested samples were diluted to 11.5 mL with 18.2 MΩcm Milli-Q Direct water (Merck Millipore, Watford, UK) and aliquots transferred to 96-well deep well plates using adjustable multichannel pipette (Rainin; Anachem Ltd, Luton, UK) for analysis. Elemental analysis was performed with an inductively coupled plasma-mass spectrometry (ICP-MS) (PerkinElmer NexION 300D equipped with Elemental Scientific Inc. autosampler and Apex HF sample introduction system; PerkinElmer LAS Ltd, Seer Green, UK and Elemental Scientific Inc., Omaha, NE, USA, respectively) in the standard mode for all the elements except for iron which was measured using the standard and dynamic reaction modes (with ammonia gas). Twenty-two elements (B, Na, Mg, P, S, K, Ca, Ti, Cr, Mn, Fe, Co, Ni, Cu, Zn, As, Se, Rb, Sr, Mo, Cd and Pb) were monitored. Liquid reference material composed of pooled samples of the digested seed materials was prepared before the beginning of sample runs and was used throughout the whole seed samples runs. It was run after every ninth sample in all ICP-MS sample sets to correct for variation between and within ICP-MS analysis runs. Also included was reference material IPE sample 192 (String bean; Wageningen Evaluation Programs for Analytical Laboratories). The calibration standards (with indium internal standard and blanks) were prepared from single element standards (Inorganic Ventures; Essex Scientific Laboratory Supplies Ltd, Essex, UK) solutions. Sample concentrations were calculated using external calibration method within the instrument software. Further data processing was

performed using Microsoft Excel spreadsheet. Protocol is outlined in Danku et al. (2013)[1].

References

1 Danku, J. M., Lahner, B., Yakubova, E. & Salt, D. E. Large-scale plant ionomics. *Plant Mineral Nutrients: Methods and Protocols*, 255-276 (2013).

SPSS Statistical Methods & Results

The following sections present the output from SPSS (version 21) for every statistical test performed on the data as described in the main text. 1) *k*-means clustering statistical analysis was carried out on weather data to determine if seasons could be justly grouped according to "rainfed" and "drought-stress" conditions. 2) An F-test was used to determine if the variation seen in yield, iron, zinc, lead and phytic acid across varieties could be attributed to genotype or the weather conditions during the field trials. 3) A one-way ANOVA was carried out to further validate the similarity in yield, iron, zinc, lead and phytic acid among varieties under both rainfed and drought-stress conditions. 4) A two-tailed independent samples t-test was carried out to analyse changes in yield and nutritional quality between rainfed and drought-stress conditions, when all bean varieties are taken as a single mixture. 5) A multiple linear regression statistical analysis was carried out to examine, in detail, which weather variable(s) can explain the changes in yield and nutritional quality.

1) k-means clustering statistical analysis

```
QUICK CLUSTER Rainfromfloweringdatetoharvest
MinTempfromfloweringdatetoharvest
    MaxTempfromfloweringdatetoharvest AvgTempfromfloweringdatetoharvest
  /MISSING=LISTWISE
  /CRITERIA=CLUSTER(2) MXITER(10) CONVERGE(0)
  /METHOD=KMEANS(NOUPDATE)
  /PRINT INITIAL.
```

Quick Cluster

Notes

Output Created		10-NOV-2017 17:18:51
Comments		
Input	Data	C:\Users\0115647s\Desktop\brendan halaran\Brendah data 2.sav
	Active Dataset	DataSet2
	Filter	<none>
	Weight	<none>
	Split File	<none>
	N of Rows in Working Data File	240
Missing Value Handling	Definition of Missing	User-defined missing values are treated as missing.

	Cases Used	Statistics are based on cases with no missing values for any clustering variable used.
Syntax		QUICK CLUSTER Rainfromfloweringdatetoharvest MinTempfromfloweringdatetoharvest MaxTempfromfloweringdatetoharvest AvgTempfromfloweringdatetoharvest /MISSING=LISTWISE /CRITERIA=CLUSTER(2) MXITER(10) CONVERGE(0) /METHOD=KMEANS(NOUPDATE) /PRINT INITIAL.
Resources	Processor Time	00:00:00.02
	Elapsed Time	00:00:00.02
	Workspace Required	656 bytes

Initial Cluster Centers

	Cluster	
	1	2
Rain from flowering date to harvest	106.40	5.90
Min Temp from flowering date to harvest	9.7	11.8
Max Temp from flowering date to harvest	30.1	34.9
Avg Temp from flowering date to harvest	21.57	22.89

Iteration History[a]

	Change in Cluster Centers	
Iteration	1	2
1	20.683	25.579
2	.000	.000

a. Convergence achieved due to no or small change in cluster centers. The maximum absolute coordinate change for any center is .000. The current iteration is 2. The minimum distance between initial centers is 100.645.

Final Cluster Centers

	Cluster	
	1	2
Rain from flowering date to harvest	85.72	31.46
Min Temp from flowering date to harvest	9.7	11.3
Max Temp from flowering date to harvest	30.1	34.4
Avg Temp from flowering date to harvest	21.59	23.49

Number of Cases in each Cluster

Cluster	1	99.000
	2	141.000
Valid		240.000
Missing		.000

```
TWOSTEP CLUSTER
  /CATEGORICAL VARIABLES=Rainfromfloweringdatetoharvest
MinTempfromfloweringdatetoharvest
    MaxTempfromfloweringdatetoharvest AvgTempfromfloweringdatetoharvest
Season
  /DISTANCE LIKELIHOOD
  /NUMCLUSTERS AUTO 2 BIC
  /HANDLENOISE 0
  /MEMALLOCATE 64
  /CRITERIA INITHRESHOLD(0) MXBRANCH(8) MXLEVEL(3)
  /VIEWMODEL DISPLAY=YES.
```

TwoStep Cluster

Notes

Output Created		10-NOV-2017 17:22:39
Comments		
Input	Data	C:\Users\0115647s\Desktop\brendan halaran\Brendah data 2.sav
	Active Dataset	DataSet2
	Filter	<none>
	Weight	<none>
	Split File	<none>
Missing Value Handling	Definition of Missing	User-defined missing values are treated as missing.
	Cases Used	Statistics are based on all cases with valid data for all variables in the analysis.

Syntax		TWOSTEP CLUSTER<br>/CATEGORICAL VARIABLES=Rainfromfloweringdatetoharvest MinTempfromfloweringdatetoharvest<br><br>MaxTempfromfloweringdatetoharvest AvgTempfromfloweringdatetoharvest Season<br>/DISTANCE LIKELIHOOD<br>/NUMCLUSTERS AUTO 2 BIC<br>/HANDLENOISE 0<br>/MEMALLOCATE 64<br>/CRITERIA INITHRESHOLD(0) MXBRANCH(8) MXLEVEL(3)<br>/VIEWMODEL DISPLAY=YES.
Resources	Processor Time	00:00:00.06
	Elapsed Time	00:00:00.06
Files Saved	Model	C:\Users\0115647s\AppData\Local\Temp\spss11888\tsctempm.24

null : null

Model Summary

**Algorithm**	TwoStep
**Inputs**	5
**Clusters**	2

Cluster Quality

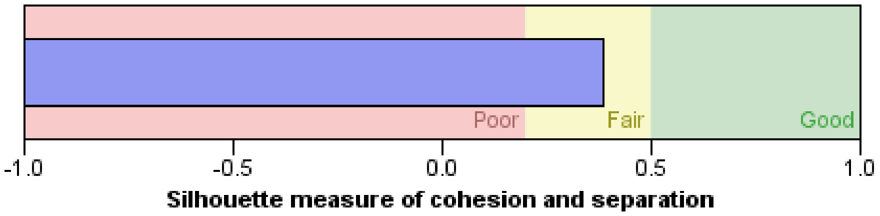

2) F-test statistical analysis

```
REGRESSION
  /DESCRIPTIVES MEAN STDDEV CORR SIG N
  /MISSING LISTWISE
  /STATISTICS COEFF OUTS CI(95) R ANOVA COLLIN TOL
  /CRITERIA=PIN(.05) POUT(.10)
  /NOORIGIN
  /DEPENDENT SQRT_Yield
  /METHOD=STEPWISE Variety Condition
  /SCATTERPLOT=(*ZRESID ,*ZPRED)
  /RESIDUALS HISTOGRAM(ZRESID) NORMPROB(ZRESID)
  /CASEWISE PLOT(ZRESID) OUTLIERS(3)
  /SAVE COOK ZRESID.
```

Regression

Notes

Output Created		27-NOV-2017 15:27:03
Comments		
Input	Data	C:\Users\Brendan\Documents\Micronutrients\Yield and micronutirents variety+condition+season.sav
	Active Dataset	DataSet1
	Filter	<none>
	Weight	<none>
	Split File	<none>
	N of Rows in Working Data File	240
Missing Value Handling	Definition of Missing	User-defined missing values are treated as missing.
	Cases Used	Statistics are based on cases with no missing values for any variable used.

Syntax		REGRESSION /DESCRIPTIVES MEAN STDDEV CORR SIG N /MISSING LISTWISE /STATISTICS COEFF OUTS CI(95) R ANOVA COLLIN TOL /CRITERIA=PIN(.05) POUT(.10) /NOORIGIN /DEPENDENT SQRT_Yield /METHOD=STEPWISE Variety Condition /SCATTERPLOT=(*ZRESID ,*ZPRED) /RESIDUALS HISTOGRAM(ZRESID) NORMPROB(ZRESID) /CASEWISE PLOT(ZRESID) OUTLIERS(3) /SAVE COOK ZRESID.
Resources	Processor Time	00:00:02.92
	Elapsed Time	00:00:03.95
	Memory Required	5632 bytes
	Additional Memory Required for Residual Plots	664 bytes
Variables Created or Modified	ZRE_7	Standardized Residual
	COO_7	Cook's Distance

Descriptive Statistics

	Mean	Std. Deviation	N
SQRT_Yield	23.809972	10.7317498	236
Variety	10.42	5.768	236
Condition	1.50	.501	236

Correlations

		SQRT_Yield	Variety	Condition
Pearson Correlation	SQRT_Yield	1.000	-.096	-.630
	Variety	-.096	1.000	-.002
	Condition	-.630	-.002	1.000
Sig. (1-tailed)	SQRT_Yield	.	.071	.000
	Variety	.071	.	.490

	Condition	.000	.490	.
N	SQRT_Yield	236	236	236
	Variety	236	236	236
	Condition	236	236	236

Variables Entered/Removed[a]

Model	Variables Entered	Variables Removed	Method
1	Condition	.	Stepwise (Criteria: Probability-of-F-to-enter <= .050, Probability-of-F-to-remove >= .100).

a. Dependent Variable: SQRT_Yield

```
REGRESSION
  /DESCRIPTIVES MEAN STDDEV CORR SIG N
  /MISSING LISTWISE
  /STATISTICS COEFF OUTS CI(95) R ANOVA COLLIN TOL
  /CRITERIA=PIN(.05) POUT(.10)
  /NOORIGIN
  /DEPENDENT Fe56_ppm
  /METHOD=STEPWISE Variety Condition
  /SCATTERPLOT=(*ZRESID ,*ZPRED)
  /RESIDUALS HISTOGRAM(ZRESID) NORMPROB(ZRESID)
  /CASEWISE PLOT(ZRESID) OUTLIERS(3)
  /SAVE COOK ZRESID.
```

Regression

Descriptive Statistics

	Mean	Std. Deviation	N
Fe56_ppm	56.524989	7.8494808	230
Variety	10.41	5.816	230
Condition	1.48	.501	230

Correlations

		Fe56_ppm	Variety	Condition
Pearson Correlation	Fe56_ppm	1.000	-.021	-.222
	Variety	-.021	1.000	-.016
	Condition	-.222	-.016	1.000
Sig. (1-tailed)	Fe56_ppm	.	.378	.000

	Variety	.378	.	.407
	Condition	.000	.407	.
N	Fe56_ppm	230	230	230
	Variety	230	230	230
	Condition	230	230	230

Variables Entered/Removed[a]

Model	Variables Entered	Variables Removed	Method
1	Condition	.	Stepwise (Criteria: Probability-of-F-to-enter <= .050, Probability-of-F-to-remove >= .100).

a. Dependent Variable: Fe56_ppm

```
REGRESSION
  /DESCRIPTIVES MEAN STDDEV CORR SIG N
  /MISSING LISTWISE
  /STATISTICS COEFF OUTS CI(95) R ANOVA COLLIN TOL
  /CRITERIA=PIN(.05) POUT(.10)
  /NOORIGIN
  /DEPENDENT Zn66_ppm
  /METHOD=STEPWISE Variety Condition
  /SCATTERPLOT=(*ZRESID ,*ZPRED)
  /RESIDUALS HISTOGRAM(ZRESID) NORMPROB(ZRESID)
  /CASEWISE PLOT(ZRESID) OUTLIERS(3)
  /SAVE COOK ZRESID.
```

Regression

Descriptive Statistics

	Mean	Std. Deviation	N
Zn66_ppm	36.928831	8.1625681	227
Variety	10.42	5.815	227
Condition	1.48	.501	227

Correlations

		Zn66_ppm	Variety	Condition
Pearson Correlation	Zn66_ppm	1.000	-.128	.203
	Variety	-.128	1.000	-.006
	Condition	.203	-.006	1.000

Sig. (1-tailed)	Zn66_ppm	.	.027	.001
	Variety	.027	.	.462
	Condition	.001	.462	.
N	Zn66_ppm	227	227	227
	Variety	227	227	227
	Condition	227	227	227

Variables Entered/Removed[a]

Model	Variables Entered	Variables Removed	Method
1	Condition	.	Stepwise (Criteria: Probability-of-F-to-enter <= .050, Probability-of-F-to-remove >= .100).

a. Dependent Variable: Zn66_ppm

```
REGRESSION
  /DESCRIPTIVES MEAN STDDEV CORR SIG N
  /MISSING LISTWISE
  /STATISTICS COEFF OUTS CI(95) R ANOVA COLLIN TOL
  /CRITERIA=PIN(.05) POUT(.10)
  /NOORIGIN
  /DEPENDENT LG10_Pb208
  /METHOD=STEPWISE Variety Condition
  /SCATTERPLOT=(*ZRESID ,*ZPRED)
  /RESIDUALS HISTOGRAM(ZRESID) NORMPROB(ZRESID)
  /CASEWISE PLOT(ZRESID) OUTLIERS(3)
  /SAVE COOK ZRESID.
```

Regression

Descriptive Statistics

	Mean	Std. Deviation	N
LG10_Pb208	-1.2540	.52050	230
Variety	10.41	5.816	230
Condition	1.48	.501	230

Correlations

		LG10_Pb208	Variety	Condition
Pearson Correlation	LG10_Pb208	1.000	.026	.525
	Variety	.026	1.000	-.016
	Condition	.525	-.016	1.000

Sig. (1-tailed)	LG10_Pb208	.	.349	.000
	Variety	.349	.	.407
	Condition	.000	.407	.
N	LG10_Pb208	230	230	230
	Variety	230	230	230
	Condition	230	230	230

Variables Entered/Removed[a]

Model	Variables Entered	Variables Removed	Method
1	Condition	.	Stepwise (Criteria: Probability-of-F-to-enter <= .050, Probability-of-F-to-remove >= .100).

a. Dependent Variable: LG10_Pb208

```
REGRESSION
  /DESCRIPTIVES MEAN STDDEV CORR SIG N
  /MISSING LISTWISE
  /STATISTICS COEFF OUTS CI(95) R ANOVA COLLIN TOL
  /CRITERIA=PIN(.05) POUT(.10)
  /NOORIGIN
  /DEPENDENT Phytic_acid_percent
  /METHOD=STEPWISE Variety Condition
  /SCATTERPLOT=(*ZRESID ,*ZPRED)
  /RESIDUALS HISTOGRAM(ZRESID) NORMPROB(ZRESID)
  /CASEWISE PLOT(ZRESID) OUTLIERS(3)
  /SAVE COOK ZRESID.
```

Regression

Descriptive Statistics

	Mean	Std. Deviation	N
Phytic_acid_percent	1.0544	.31563	229
Variety	10.44	5.818	229
Condition	1.48	.501	229

Correlations

		Phytic_acid_percent	Variety	Condition
Pearson Correlation	Phytic_acid_percent	1.000	.038	.310
	Variety	.038	1.000	-.020
	Condition	.310	-.020	1.000

Sig. (1-tailed)	Phytic_acid_percent	.	.285	.000
	Variety	.285	.	.384
	Condition	.000	.384	.
N	Phytic_acid_percent	229	229	229
	Variety	229	229	229
	Condition	229	229	229

Variables Entered/Removed[a]

Model	Variables Entered	Variables Removed	Method
1	Condition	.	Stepwise (Criteria: Probability-of-F-to-enter <= .050, Probability-of-F-to-remove >= .100).

a. Dependent Variable: Phytic_acid_percent

```
DATASET ACTIVATE DataSet1.

SAVE OUTFILE='C:\Users\Brendan\Documents\Micronutrients\Yield and
micronutirents '+
    'variety+condition+season.sav'
  /COMPRESSED.
REGRESSION
  /DESCRIPTIVES MEAN STDDEV CORR SIG N
  /MISSING LISTWISE
  /STATISTICS COEFF OUTS CI(95) R ANOVA
  /CRITERIA=PIN(.05) POUT(.10)
  /NOORIGIN
  /DEPENDENT Total_protein_mg_per_g_dw
  /METHOD=STEPWISE Variety Condition
  /SCATTERPLOT=(*ZRESID ,*ZPRED)
  /RESIDUALS HISTOGRAM(ZRESID) NORMPROB(ZRESID).
```

Regression

Notes

Output Created	05-JUN-2018 12:05:37
Comments	

Input	Data	C:\Users\Brendan\Documents\Micronutrients\Yield and micronutirents variety+condition+season.sav
	Active Dataset	DataSet1
	Filter	<none>
	Weight	<none>
	Split File	<none>
	N of Rows in Working Data File	240
Missing Value Handling	Definition of Missing	User-defined missing values are treated as missing.
	Cases Used	Statistics are based on cases with no missing values for any variable used.
Syntax		REGRESSION /DESCRIPTIVES MEAN STDDEV CORR SIG N /MISSING LISTWISE /STATISTICS COEFF OUTS CI(95) R ANOVA /CRITERIA=PIN(.05) POUT(.10) /NOORIGIN /DEPENDENT Total_protein_mg_per_g_dw /METHOD=STEPWISE Variety Condition /SCATTERPLOT=(*ZRESID ,*ZPRED) /RESIDUALS HISTOGRAM(ZRESID) NORMPROB(ZRESID).
Resources	Processor Time	00:00:01.55
	Elapsed Time	00:00:00.64
	Memory Required	6432 bytes
	Additional Memory Required for Residual Plots	664 bytes

Descriptive Statistics

	Mean	Std. Deviation	N
Total_protein_mg_per_g_dw	347.2791	76.44644	230

Variety	10.41	5.816	230
Condition	1.48	.501	230

Correlations

		Total_protein_mg_per_g_dw	Variety	Condition
Pearson Correlation	Total_protein_mg_per_g_dw	1.000	.021	.297
	Variety	.021	1.000	-.016
	Condition	.297	-.016	1.000
Sig. (1-tailed)	Total_protein_mg_per_g_dw	.	.378	.000
	Variety	.378	.	.407
	Condition	.000	.407	.
N	Total_protein_mg_per_g_dw	230	230	230
	Variety	230	230	230
	Condition	230	230	230

Variables Entered/Removed[a]

Model	Variables Entered	Variables Removed	Method
1	Condition	.	Stepwise (Criteria: Probability-of-F-to-enter <= .050, Probability-of-F-to-remove >= .100).

a. Dependent Variable: Total_protein_mg_per_g_dw

3) One-way ANOVA

```
USE ALL.
COMPUTE filter_$=(Condition = 1).
VARIABLE LABELS filter_$ 'Condition = 1 (FILTER)'.
VALUE LABELS filter_$ 0 'Not Selected' 1 'Selected'.
FORMATS filter_$ (f1.0).
FILTER BY filter_$.
EXECUTE.
ONEWAY SQRT_Yield BY Variety
  /STATISTICS HOMOGENEITY
  /MISSING ANALYSIS
  /POSTHOC=TUKEY BONFERRONI ALPHA(0.05).
```

Oneway

Notes

Output Created		23-NOV-2017 18:20:18
Comments		
Input	Data	C:\Users\Brendan\Documents\Micronutrients\Yield and micronutirents variety+condition+season.sav
	Active Dataset	DataSet1
	Filter	Condition = 1 (FILTER)
	Weight	<none>
	Split File	<none>
	N of Rows in Working Data File	120
Missing Value Handling	Definition of Missing	User-defined missing values are treated as missing.
	Cases Used	Statistics for each analysis are based on cases with no missing data for any variable in the analysis.
Syntax		ONEWAY SQRT_Yield BY Variety /STATISTICS HOMOGENEITY /MISSING ANALYSIS /POSTHOC=TUKEY BONFERRONI ALPHA(0.05).
Resources	Processor Time	00:00:00.16
	Elapsed Time	00:00:00.31

Test of Homogeneity of Variances

SQRT_Yield

Levene Statistic	df1	df2	Sig.
1.116	19	99	.348

ANOVA

SQRT_Yield

	Sum of Squares	df	Mean Square	F	Sig.
Between Groups	1108.398	19	58.337	1.255	.231
Within Groups	4600.100	99	46.466		
Total	5708.499	118			

```
USE ALL.
COMPUTE filter_$=(Condition = 2).
VARIABLE LABELS filter_$ 'Condition = 2 (FILTER)'.
VALUE LABELS filter_$ 0 'Not Selected' 1 'Selected'.
FORMATS filter_$ (f1.0).
FILTER BY filter_$.
EXECUTE.
ONEWAY SQRT_Yield BY Variety
  /STATISTICS HOMOGENEITY
  /MISSING ANALYSIS
  /POSTHOC=TUKEY BONFERRONI ALPHA(0.05).
```

Oneway

Test of Homogeneity of Variances

SQRT_Yield

Levene Statistic	df1	df2	Sig.
4.222	19	97	.000

ANOVA

SQRT_Yield

	Sum of Squares	df	Mean Square	F	Sig.
Between Groups	2093.999	19	110.210	1.255	.232
Within Groups	8520.053	97	87.836		
Total	10614.052	116			

```
USE ALL.
COMPUTE filter_$=(Condition = 1).
VARIABLE LABELS filter_$ 'Condition = 1 (FILTER)'.
VALUE LABELS filter_$ 0 'Not Selected' 1 'Selected'.
FORMATS filter_$ (f1.0).
FILTER BY filter_$.
EXECUTE.
ONEWAY Fe56_ppm BY Variety
  /STATISTICS HOMOGENEITY
  /MISSING ANALYSIS
  /POSTHOC=TUKEY BONFERRONI ALPHA(0.05).
```

Oneway

Notes

Output Created	23-NOV-2017 18:26:01

Comments		
Input	Data	C:\Users\Brendan\Documents\Micronutrients\Yield and micronutirents variety+condition+season.sav
	Active Dataset	DataSet1
	Filter	Condition = 1 (FILTER)
	Weight	<none>
	Split File	<none>
	N of Rows in Working Data File	120
Missing Value Handling	Definition of Missing	User-defined missing values are treated as missing.
	Cases Used	Statistics for each analysis are based on cases with no missing data for any variable in the analysis.
Syntax		ONEWAY Fe56_ppm BY Variety /STATISTICS HOMOGENEITY /MISSING ANALYSIS /POSTHOC=TUKEY BONFERRONI ALPHA(0.05).
Resources	Processor Time	00:00:00.11
	Elapsed Time	00:00:00.10

Test of Homogeneity of Variances

Fe56_ppm

Levene Statistic	df1	df2	Sig.
1.400	19	100	.144

ANOVA

Fe56_ppm

	Sum of Squares	df	Mean Square	F	Sig.
Between Groups	680.420	19	35.812	.926	.553
Within Groups	3865.692	100	38.657		
Total	4546.112	119			

```
USE ALL.
COMPUTE filter_$=(Condition = 2).
VARIABLE LABELS filter_$ 'Condition = 2 (FILTER)'.
```

```
VALUE LABELS filter_$ 0 'Not Selected' 1 'Selected'.
FORMATS filter_$ (f1.0).
FILTER BY filter_$.
EXECUTE.
ONEWAY Fe56_ppm BY Variety
  /STATISTICS HOMOGENEITY
  /MISSING ANALYSIS
  /POSTHOC=TUKEY BONFERRONI ALPHA(0.05).
```

Oneway

Notes

Output Created		23-NOV-2017 18:29:31
Comments		
Input	Data	C:\Users\Brendan\Documents\Micronutrients\Yield and micronutirents variety+condition+season.sav
	Active Dataset	DataSet1
	Filter	Condition = 2 (FILTER)
	Weight	<none>
	Split File	<none>
	N of Rows in Working Data File	120
Missing Value Handling	Definition of Missing	User-defined missing values are treated as missing.
	Cases Used	Statistics for each analysis are based on cases with no missing data for any variable in the analysis.
Syntax		ONEWAY Fe56_ppm BY Variety /STATISTICS HOMOGENEITY /MISSING ANALYSIS /POSTHOC=TUKEY BONFERRONI ALPHA(0.05).
Resources	Processor Time	00:00:00.09
	Elapsed Time	00:00:00.10

Test of Homogeneity of Variances

Fe56_ppm

Levene Statistic	df1	df2	Sig.
1.044	19	90	.421

ANOVA

Fe56_ppm

	Sum of Squares	df	Mean Square	F	Sig.
Between Groups	1627.978	19	85.683	1.065	.399
Within Groups	7237.926	90	80.421		
Total	8865.903	109			

```
USE ALL.
COMPUTE filter_$=(Condition = 1).
VARIABLE LABELS filter_$ 'Condition = 1 (FILTER)'.
VALUE LABELS filter_$ 0 'Not Selected' 1 'Selected'.
FORMATS filter_$ (f1.0).
FILTER BY filter_$.
EXECUTE.
ONEWAY Zn66_ppm BY Variety
  /STATISTICS HOMOGENEITY
  /MISSING ANALYSIS
  /POSTHOC=TUKEY BONFERRONI ALPHA(0.05).
```

Oneway

Notes

Output Created		23-NOV-2017 18:30:56
Comments		
Input	Data	C:\Users\Brendan\Documents\Micronutrients\Yield and micronutirents variety+condition+season.sav
	Active Dataset	DataSet1
	Filter	Condition = 1 (FILTER)
	Weight	<none>
	Split File	<none>
	N of Rows in Working Data File	120
Missing Value Handling	Definition of Missing	User-defined missing values are treated as missing.
	Cases Used	Statistics for each analysis are based on cases with no missing data for any variable in the analysis.

Syntax		ONEWAY Zn66_ppm BY Variety<br>/STATISTICS HOMOGENEITY<br>/MISSING ANALYSIS<br>/POSTHOC=TUKEY BONFERRONI ALPHA(0.05).
Resources	Processor Time	00:00:00.09
	Elapsed Time	00:00:00.11

Test of Homogeneity of Variances

Zn66_ppm

Levene Statistic	df1	df2	Sig.
1.496	19	99	.103

ANOVA

Zn66_ppm

	Sum of Squares	df	Mean Square	F	Sig.
Between Groups	1607.768	19	84.619	1.470	.114
Within Groups	5700.091	99	57.577		
Total	7307.859	118			

```
USE ALL.
COMPUTE filter_$=(Condition = 2).
VARIABLE LABELS filter_$ 'Condition = 2 (FILTER)'.
VALUE LABELS filter_$ 0 'Not Selected' 1 'Selected'.
FORMATS filter_$ (f1.0).
FILTER BY filter_$.
EXECUTE.
ONEWAY Zn66_ppm BY Variety
  /STATISTICS HOMOGENEITY
  /MISSING ANALYSIS
  /POSTHOC=TUKEY BONFERRONI ALPHA(0.05).
```

Oneway

Test of Homogeneity of Variances

Zn66_ppm

Levene Statistic	df1	df2	Sig.
4.179	19	88	.000

ANOVA

Zn66_ppm

	Sum of Squares	df	Mean Square	F	Sig.

Between Groups	2473.228	19	130.170	2.459	.002
Within Groups	4658.904	88	52.942		
Total	7132.132	107			

Post Hoc Tests

Multiple Comparisons

Dependent Variable: Zn66_ppm

	(I) Variety	(J) Variety	Mean Difference (I-J)	Std. Error	Sig.	95% Confidence Interval Lower Bound	Upper Bound
Tukey HSD	A197	A286	2.1717947	4.4059165	1.000	-13.928047	18.271637
		A334	.0795760	4.2008765	1.000	-15.271021	15.430173
		CAL113	-5.6127554	4.2008765	.998	-20.963353	9.737842
		CAL 143	-5.4637929	4.2008765	.999	-20.814390	9.886804
		DRK 57	.4596262	4.2008765	1.000	-14.890971	15.810223
		NASAKA	1.9688614	4.4059165	1.000	-14.130981	18.068704
		NUA 45	.3409424	4.2008765	1.000	-15.009655	15.691540
		NUA 59	-18.8178737*	4.6967228	.017	-35.980363	-1.655384
		NUA 674	2.8080257	5.1450020	1.000	-15.992539	21.608591
		NUA 705	-1.8747725	4.2008765	1.000	-17.225370	13.475825
		NUA 706	2.9747204	4.4059165	1.000	-13.125122	19.074563
		NUA 720	.0319731	4.2008765	1.000	-15.318624	15.382570
		NUA 730	3.6645309	4.4059165	1.000	-12.435311	19.764373
		NUA 740	6.3014432	4.2008765	.993	-9.049154	21.652040
		NUA 743	3.7737121	4.2008765	1.000	-11.576885	19.124309
		NUA 746	.9219538	4.2008765	1.000	-14.428643	16.272551
		SUGAR 113	-.6618439	4.6967228	1.000	-17.824333	16.500646
		UBT (92) 25	5.1238469	4.2008765	1.000	-10.226750	20.474444
		VTTT 294/4-4	.3075969	4.4059165	1.000	-15.792245	16.407439
	A286	A197	-2.1717947	4.4059165	1.000	-18.271637	13.928047
		A334	-2.0922187	4.4059165	1.000	-18.192061	14.007623
		CAL113	-7.7845502	4.4059165	.963	-23.884392	8.315292
		CAL 143	-7.6355877	4.4059165	.970	-23.735430	8.464255
		DRK 57	-1.7121685	4.4059165	1.000	-17.812011	14.387674
		NASAKA	-.2029333	4.6018297	1.000	-17.018670	16.612803
		NUA 45	-1.8308523	4.4059165	1.000	-17.930694	14.268990
		NUA 59	-20.9896685*	4.8809775	.006	-38.825451	-3.153886
		NUA 674	.6362310	5.3137352	1.000	-18.780909	20.053371
		NUA 705	-4.0465672	4.4059165	1.000	-20.146409	12.053275
		NUA 706	.8029256	4.6018297	1.000	-16.012811	17.618662

	NUA 720	-2.1398216	4.4059165	1.000	-18.239664	13.960021
	NUA 730	1.4927362	4.6018297	1.000	-15.323001	18.308473
	NUA 740	4.1296484	4.4059165	1.000	-11.970194	20.229491
	NUA 743	1.6019173	4.4059165	1.000	-14.497925	17.701760
	NUA 746	-1.2498410	4.4059165	1.000	-17.349683	14.850001
	SUGAR 113	-2.8336386	4.8809775	1.000	-20.669421	15.002144
	UBT (92) 25	2.9520522	4.4059165	1.000	-13.147790	19.051894
	VTTT 294/4-4	-1.8641978	4.6018297	1.000	-18.679935	14.951539
A334	A197	-.0795760	4.2008765	1.000	-15.430173	15.271021
	A286	2.0922187	4.4059165	1.000	-14.007623	18.192061
	CAL113	-5.6923315	4.2008765	.998	-21.042929	9.658266
	CAL 143	-5.5433690	4.2008765	.999	-20.893966	9.807228
	DRK 57	.3800502	4.2008765	1.000	-14.970547	15.730647
	NASAKA	1.8892854	4.4059165	1.000	-14.210557	17.989128
	NUA 45	.2613664	4.2008765	1.000	-15.089231	15.611964
	NUA 59	-18.8974498*	4.6967228	.016	-36.059939	-1.734960
	NUA 674	2.7284497	5.1450020	1.000	-16.072116	21.529015
	NUA 705	-1.9543485	4.2008765	1.000	-17.304946	13.396249
	NUA 706	2.8951443	4.4059165	1.000	-13.204698	18.994987
	NUA 720	-.0476029	4.2008765	1.000	-15.398200	15.302994
	NUA 730	3.5849549	4.4059165	1.000	-12.514887	19.684797
	NUA 740	6.2218672	4.2008765	.994	-9.128730	21.572464
	NUA 743	3.6941360	4.2008765	1.000	-11.656461	19.044733
	NUA 746	.8423777	4.2008765	1.000	-14.508219	16.192975
	SUGAR 113	-.7414199	4.6967228	1.000	-17.903909	16.421070
	UBT (92) 25	5.0442709	4.2008765	1.000	-10.306326	20.394868
	VTTT 294/4-4	.2280209	4.4059165	1.000	-15.871821	16.327863
CAL113	A197	5.6127554	4.2008765	.998	-9.737842	20.963353
	A286	7.7845502	4.4059165	.963	-8.315292	23.884392
	A334	5.6923315	4.2008765	.998	-9.658266	21.042929
	CAL 143	.1489625	4.2008765	1.000	-15.201635	15.499560
	DRK 57	6.0723816	4.2008765	.996	-9.278216	21.422979
	NASAKA	7.5816168	4.4059165	.972	-8.518225	23.681459
	NUA 45	5.9536978	4.2008765	.997	-9.396899	21.304295
	NUA 59	-13.2051183	4.6967228	.366	-30.367608	3.957371
	NUA 674	8.4207812	5.1450020	.983	-10.379784	27.221346
	NUA 705	3.7379830	4.2008765	1.000	-11.612614	19.088580
	NUA 706	8.5874758	4.4059165	.913	-7.512366	24.687318
	NUA 720	5.6447286	4.2008765	.998	-9.705869	20.995326
	NUA 730	9.2772863	4.4059165	.845	-6.822556	25.377128

		NUA 740	11.9141986	4.2008765	.351	-3.436399	27.264796
		NUA 743	9.3864675	4.2008765	.771	-5.964130	24.737065
		NUA 746	6.5347092	4.2008765	.990	-8.815888	21.885306
		SUGAR 113	4.9509116	4.6967228	1.000	-12.211578	22.113401
		UBT (92) 25	10.7366023	4.2008765	.546	-4.613995	26.087200
		VTTT 294/4-4	5.9203523	4.4059165	.998	-10.179490	22.020195
	CAL 143	A197	5.4637929	4.2008765	.999	-9.886804	20.814390
		A286	7.6355877	4.4059165	.970	-8.464255	23.735430
		A334	5.5433690	4.2008765	.999	-9.807228	20.893966
		CAL113	-.1489625	4.2008765	1.000	-15.499560	15.201635
		DRK 57	5.9234191	4.2008765	.997	-9.427178	21.274016
		NASAKA	7.4326544	4.4059165	.977	-8.667188	23.532497
		NUA 45	5.8047354	4.2008765	.998	-9.545862	21.155333
		NUA 59	-13.3540808	4.6967228	.346	-30.516570	3.808409
		NUA 674	8.2718187	5.1450020	.986	-10.528747	27.072384
		NUA 705	3.5890205	4.2008765	1.000	-11.761577	18.939618
		NUA 706	8.4385133	4.4059165	.925	-7.661329	24.538355
		NUA 720	5.4957661	4.2008765	.999	-9.854831	20.846363
		NUA 730	9.1283238	4.4059165	.862	-6.971518	25.228166
		NUA 740	11.7652361	4.2008765	.373	-3.585361	27.115833
		NUA 743	9.2375050	4.2008765	.793	-6.113092	24.588102
		NUA 746	6.3857467	4.2008765	.992	-8.964851	21.736344
		SUGAR 113	4.8019491	4.6967228	1.000	-12.360540	21.964439
		UBT (92) 25	10.5876399	4.2008765	.572	-4.762957	25.938237
		VTTT 294/4-4	5.7713898	4.4059165	.999	-10.328452	21.871232
	DRK 57	A197	-.4596262	4.2008765	1.000	-15.810223	14.890971
		A286	1.7121685	4.4059165	1.000	-14.387674	17.812011
		A334	-.3800502	4.2008765	1.000	-15.730647	14.970547
		CAL113	-6.0723816	4.2008765	.996	-21.422979	9.278216
		CAL 143	-5.9234191	4.2008765	.997	-21.274016	9.427178
		NASAKA	1.5092352	4.4059165	1.000	-14.590607	17.609077
		NUA 45	-.1186838	4.2008765	1.000	-15.469281	15.231913
		NUA 59	-19.2774999*	4.6967228	.012	-36.439989	-2.115010
		NUA 674	2.3483995	5.1450020	1.000	-16.452166	21.148965
		NUA 705	-2.3343986	4.2008765	1.000	-17.684996	13.016199
		NUA 706	2.5150942	4.4059165	1.000	-13.584748	18.614936
		NUA 720	-.4276531	4.2008765	1.000	-15.778250	14.922944
		NUA 730	3.2049047	4.4059165	1.000	-12.894937	19.304747
		NUA 740	5.8418170	4.2008765	.997	-9.508780	21.192414
		NUA 743	3.3140859	4.2008765	1.000	-12.036511	18.664683

		NUA 746	.4623276	4.2008765	1.000	-14.888270	15.812925
		SUGAR 113	-1.1214700	4.6967228	1.000	-18.283959	16.041019
		UBT (92) 25	4.6642207	4.2008765	1.000	-10.686376	20.014818
		VTTT 294/4-4	-.1520293	4.4059165	1.000	-16.251871	15.947813
	NASAKA	A197	-1.9688614	4.4059165	1.000	-18.068704	14.130981
		A286	.2029333	4.6018297	1.000	-16.612803	17.018670
		A334	-1.8892854	4.4059165	1.000	-17.989128	14.210557
		CAL113	-7.5816168	4.4059165	.972	-23.681459	8.518225
		CAL 143	-7.4326544	4.4059165	.977	-23.532497	8.667188
		DRK 57	-1.5092352	4.4059165	1.000	-17.609077	14.590607
		NUA 45	-1.6279190	4.4059165	1.000	-17.727761	14.471923
		NUA 59	-20.7867351*	4.8809775	.007	-38.622517	-2.950953
		NUA 674	.8391643	5.3137352	1.000	-18.577976	20.256305
		NUA 705	-3.8436339	4.4059165	1.000	-19.943476	12.256208
		NUA 706	1.0058590	4.6018297	1.000	-15.809878	17.821596
		NUA 720	-1.9368883	4.4059165	1.000	-18.036730	14.162954
		NUA 730	1.6956695	4.6018297	1.000	-15.120067	18.511406
		NUA 740	4.3325818	4.4059165	1.000	-11.767260	20.432424
		NUA 743	1.8048506	4.4059165	1.000	-14.294992	17.904693
		NUA 746	-1.0469077	4.4059165	1.000	-17.146750	15.052935
		SUGAR 113	-2.6307053	4.8809775	1.000	-20.466487	15.205077
		UBT (92) 25	3.1549855	4.4059165	1.000	-12.944857	19.254828
		VTTT 294/4-4	-1.6612645	4.6018297	1.000	-18.477001	15.154472
	NUA 45	A197	-.3409424	4.2008765	1.000	-15.691540	15.009655
		A286	1.8308523	4.4059165	1.000	-14.268990	17.930694
		A334	-.2613664	4.2008765	1.000	-15.611964	15.089231
		CAL113	-5.9536978	4.2008765	.997	-21.304295	9.396899
		CAL 143	-5.8047354	4.2008765	.998	-21.155333	9.545862
		DRK 57	.1186838	4.2008765	1.000	-15.231913	15.469281
		NASAKA	1.6279190	4.4059165	1.000	-14.471923	17.727761
		NUA 59	-19.1588162*	4.6967228	.013	-36.321306	-1.996327
		NUA 674	2.4670833	5.1450020	1.000	-16.333482	21.267649
		NUA 705	-2.2157149	4.2008765	1.000	-17.566312	13.134882
		NUA 706	2.6337779	4.4059165	1.000	-13.466064	18.733620
		NUA 720	-.3089693	4.2008765	1.000	-15.659567	15.041628
		NUA 730	3.3235885	4.4059165	1.000	-12.776254	19.423431
		NUA 740	5.9605008	4.2008765	.997	-9.390096	21.311098
		NUA 743	3.4327696	4.2008765	1.000	-11.917828	18.783367
		NUA 746	.5810113	4.2008765	1.000	-14.769586	15.931609
		SUGAR 113	-1.0027863	4.6967228	1.000	-18.165276	16.159703

	UBT (92) 25	4.7829045	4.2008765	1.000	-10.567693	20.133502
	VTTT 294/4-4	-.0333455	4.4059165	1.000	-16.133188	16.066497
NUA 59	A197	18.8178737*	4.6967228	.017	1.655384	35.980363
	A286	20.9896685*	4.8809775	.006	3.153886	38.825451
	A334	18.8974498*	4.6967228	.016	1.734960	36.059939
	CAL113	13.2051183	4.6967228	.366	-3.957371	30.367608
	CAL 143	13.3540808	4.6967228	.346	-3.808409	30.516570
	DRK 57	19.2774999*	4.6967228	.012	2.115010	36.439989
	NASAKA	20.7867351*	4.8809775	.007	2.950953	38.622517
	NUA 45	19.1588162*	4.6967228	.013	1.996327	36.321306
	NUA 674	21.6258995*	5.5572373	.025	1.318968	41.932831
	NUA 705	16.9431013	4.6967228	.057	-.219388	34.105591
	NUA 706	21.7925941*	4.8809775	.004	3.956812	39.628376
	NUA 720	18.8498469*	4.6967228	.017	1.687357	36.012336
	NUA 730	22.4824046*	4.8809775	.002	4.646622	40.318187
	NUA 740	25.1193169*	4.6967228	.000	7.956827	42.281806
	NUA 743	22.5915858*	4.6967228	.001	5.429096	39.754075
	NUA 746	19.7398275*	4.6967228	.009	2.577338	36.902317
	SUGAR 113	18.1560299	5.1450020	.071	-.644535	36.956595
	UBT (92) 25	23.9417206*	4.6967228	.000	6.779231	41.104210
	VTTT 294/4-4	19.1254706*	4.8809775	.023	1.289688	36.961253
NUA 674	A197	-2.8080257	5.1450020	1.000	-21.608591	15.992539
	A286	-.6362310	5.3137352	1.000	-20.053371	18.780909
	A334	-2.7284497	5.1450020	1.000	-21.529015	16.072116
	CAL113	-8.4207812	5.1450020	.983	-27.221346	10.379784
	CAL 143	-8.2718187	5.1450020	.986	-27.072384	10.528747
	DRK 57	-2.3483995	5.1450020	1.000	-21.148965	16.452166
	NASAKA	-.8391643	5.3137352	1.000	-20.256305	18.577976
	NUA 45	-2.4670833	5.1450020	1.000	-21.267649	16.333482
	NUA 59	-21.6258995*	5.5572373	.025	-41.932831	-1.318968
	NUA 705	-4.6827982	5.1450020	1.000	-23.483363	14.117767
	NUA 706	.1666946	5.3137352	1.000	-19.250446	19.583835
	NUA 720	-2.7760526	5.1450020	1.000	-21.576618	16.024513
	NUA 730	.8565052	5.3137352	1.000	-18.560635	20.273645
	NUA 740	3.4934175	5.1450020	1.000	-15.307148	22.293983
	NUA 743	.9656863	5.1450020	1.000	-17.834879	19.766252
	NUA 746	-1.8860720	5.1450020	1.000	-20.686637	16.914493
	SUGAR 113	-3.4698696	5.5572373	1.000	-23.776801	16.837062
	UBT (92) 25	2.3158212	5.1450020	1.000	-16.484744	21.116386
	VTTT 294/4-4	-2.5004288	5.3137352	1.000	-21.917569	16.916711

NUA 705	A197	1.8747725	4.2008765	1.000	-13.475825	17.225370
	A286	4.0465672	4.4059165	1.000	-12.053275	20.146409
	A334	1.9543485	4.2008765	1.000	-13.396249	17.304946
	CAL113	-3.7379830	4.2008765	1.000	-19.088580	11.612614
	CAL 143	-3.5890205	4.2008765	1.000	-18.939618	11.761577
	DRK 57	2.3343986	4.2008765	1.000	-13.016199	17.684996
	NASAKA	3.8436339	4.4059165	1.000	-12.256208	19.943476
	NUA 45	2.2157149	4.2008765	1.000	-13.134882	17.566312
	NUA 59	-16.9431013	4.6967228	.057	-34.105591	.219388
	NUA 674	4.6827982	5.1450020	1.000	-14.117767	23.483363
	NUA 706	4.8494928	4.4059165	1.000	-11.250349	20.949335
	NUA 720	1.9067456	4.2008765	1.000	-13.443852	17.257343
	NUA 730	5.5393033	4.4059165	.999	-10.560539	21.639146
	NUA 740	8.1762156	4.2008765	.914	-7.174382	23.526813
	NUA 743	5.6484845	4.2008765	.998	-9.702113	20.999082
	NUA 746	2.7967262	4.2008765	1.000	-12.553871	18.147323
	SUGAR 113	1.2129286	4.6967228	1.000	-15.949561	18.375418
	UBT (92) 25	6.9986194	4.2008765	.979	-8.351978	22.349217
	VTTT 294/4-4	2.1823694	4.4059165	1.000	-13.917473	18.282212
NUA 706	A197	-2.9747204	4.4059165	1.000	-19.074563	13.125122
	A286	-.8029256	4.6018297	1.000	-17.618662	16.012811
	A334	-2.8951443	4.4059165	1.000	-18.994987	13.204698
	CAL113	-8.5874758	4.4059165	.913	-24.687318	7.512366
	CAL 143	-8.4385133	4.4059165	.925	-24.538355	7.661329
	DRK 57	-2.5150942	4.4059165	1.000	-18.614936	13.584748
	NASAKA	-1.0058590	4.6018297	1.000	-17.821596	15.809878
	NUA 45	-2.6337779	4.4059165	1.000	-18.733620	13.466064
	NUA 59	-21.7925941*	4.8809775	.004	-39.628376	-3.956812
	NUA 674	-.1666946	5.3137352	1.000	-19.583835	19.250446
	NUA 705	-4.8494928	4.4059165	1.000	-20.949335	11.250349
	NUA 720	-2.9427472	4.4059165	1.000	-19.042589	13.157095
	NUA 730	.6898105	4.6018297	1.000	-16.125926	17.505547
	NUA 740	3.3267228	4.4059165	1.000	-12.773119	19.426565
	NUA 743	.7989917	4.4059165	1.000	-15.300850	16.898834
	NUA 746	-2.0527666	4.4059165	1.000	-18.152609	14.047076
	SUGAR 113	-3.6365642	4.8809775	1.000	-21.472346	14.199218
	UBT (92) 25	2.1491265	4.4059165	1.000	-13.950716	18.248969
	VTTT 294/4-4	-2.6671235	4.6018297	1.000	-19.482860	14.148613
NUA 720	A197	-.0319731	4.2008765	1.000	-15.382570	15.318624
	A286	2.1398216	4.4059165	1.000	-13.960021	18.239664

	A334	.0476029	4.2008765	1.000	-15.302994	15.398200
	CAL113	-5.6447286	4.2008765	.998	-20.995326	9.705869
	CAL 143	-5.4957661	4.2008765	.999	-20.846363	9.854831
	DRK 57	.4276531	4.2008765	1.000	-14.922944	15.778250
	NASAKA	1.9368883	4.4059165	1.000	-14.162954	18.036730
	NUA 45	.3089693	4.2008765	1.000	-15.041628	15.659567
	NUA 59	-18.8498469*	4.6967228	.017	-36.012336	-1.687357
	NUA 674	2.7760526	5.1450020	1.000	-16.024513	21.576618
	NUA 705	-1.9067456	4.2008765	1.000	-17.257343	13.443852
	NUA 706	2.9427472	4.4059165	1.000	-13.157095	19.042589
	NUA 730	3.6325578	4.4059165	1.000	-12.467284	19.732400
	NUA 740	6.2694701	4.2008765	.994	-9.081127	21.620067
	NUA 743	3.7417389	4.2008765	1.000	-11.608858	19.092336
	NUA 746	.8899806	4.2008765	1.000	-14.460617	16.240578
	SUGAR 113	-.6938170	4.6967228	1.000	-17.856306	16.468672
	UBT (92) 25	5.0918738	4.2008765	1.000	-10.258723	20.442471
	VTTT 294/4-4	.2756238	4.4059165	1.000	-15.824218	16.375466
NUA 730	A197	-3.6645309	4.4059165	1.000	-19.764373	12.435311
	A286	-1.4927362	4.6018297	1.000	-18.308473	15.323001
	A334	-3.5849549	4.4059165	1.000	-19.684797	12.514887
	CAL113	-9.2772863	4.4059165	.845	-25.377128	6.822556
	CAL 143	-9.1283238	4.4059165	.862	-25.228166	6.971518
	DRK 57	-3.2049047	4.4059165	1.000	-19.304747	12.894937
	NASAKA	-1.6956695	4.6018297	1.000	-18.511406	15.120067
	NUA 45	-3.3235885	4.4059165	1.000	-19.423431	12.776254
	NUA 59	-22.4824046*	4.8809775	.002	-40.318187	-4.646622
	NUA 674	-.8565052	5.3137352	1.000	-20.273645	18.560635
	NUA 705	-5.5393033	4.4059165	.999	-21.639146	10.560539
	NUA 706	-.6898105	4.6018297	1.000	-17.505547	16.125926
	NUA 720	-3.6325578	4.4059165	1.000	-19.732400	12.467284
	NUA 740	2.6369123	4.4059165	1.000	-13.462930	18.736754
	NUA 743	.1091812	4.4059165	1.000	-15.990661	16.209023
	NUA 746	-2.7425771	4.4059165	1.000	-18.842419	13.357265
	SUGAR 113	-4.3263747	4.8809775	1.000	-22.162157	13.509407
	UBT (92) 25	1.4593160	4.4059165	1.000	-14.640526	17.559158
	VTTT 294/4-4	-3.3569340	4.6018297	1.000	-20.172671	13.458803
NUA 740	A197	-6.3014432	4.2008765	.993	-21.652040	9.049154
	A286	-4.1296484	4.4059165	1.000	-20.229491	11.970194
	A334	-6.2218672	4.2008765	.994	-21.572464	9.128730
	CAL113	-11.9141986	4.2008765	.351	-27.264796	3.436399

		CAL 143	-11.7652361	4.2008765	.373	-27.115833	3.585361
		DRK 57	-5.8418170	4.2008765	.997	-21.192414	9.508780
		NASAKA	-4.3325818	4.4059165	1.000	-20.432424	11.767260
		NUA 45	-5.9605008	4.2008765	.997	-21.311098	9.390096
		NUA 59	-25.1193169*	4.6967228	.000	-42.281806	-7.956827
		NUA 674	-3.4934175	5.1450020	1.000	-22.293983	15.307148
		NUA 705	-8.1762156	4.2008765	.914	-23.526813	7.174382
		NUA 706	-3.3267228	4.4059165	1.000	-19.426565	12.773119
		NUA 720	-6.2694701	4.2008765	.994	-21.620067	9.081127
		NUA 730	-2.6369123	4.4059165	1.000	-18.736754	13.462930
		NUA 743	-2.5277311	4.2008765	1.000	-17.878328	12.822866
		NUA 746	-5.3794894	4.2008765	.999	-20.730087	9.971108
		SUGAR 113	-6.9632870	4.6967228	.994	-24.125776	10.199202
		UBT (92) 25	-1.1775963	4.2008765	1.000	-16.528193	14.173001
		VTTT 294/4-4	-5.9938463	4.4059165	.998	-22.093688	10.105996
	NUA 743	A197	-3.7737121	4.2008765	1.000	-19.124309	11.576885
		A286	-1.6019173	4.4059165	1.000	-17.701760	14.497925
		A334	-3.6941360	4.2008765	1.000	-19.044733	11.656461
		CAL113	-9.3864675	4.2008765	.771	-24.737065	5.964130
		CAL 143	-9.2375050	4.2008765	.793	-24.588102	6.113092
		DRK 57	-3.3140859	4.2008765	1.000	-18.664683	12.036511
		NASAKA	-1.8048506	4.4059165	1.000	-17.904693	14.294992
		NUA 45	-3.4327696	4.2008765	1.000	-18.783367	11.917828
		NUA 59	-22.5915858*	4.6967228	.001	-39.754075	-5.429096
		NUA 674	-.9656863	5.1450020	1.000	-19.766252	17.834879
		NUA 705	-5.6484845	4.2008765	.998	-20.999082	9.702113
		NUA 706	-.7989917	4.4059165	1.000	-16.898834	15.300850
		NUA 720	-3.7417389	4.2008765	1.000	-19.092336	11.608858
		NUA 730	-.1091812	4.4059165	1.000	-16.209023	15.990661
		NUA 740	2.5277311	4.2008765	1.000	-12.822866	17.878328
		NUA 746	-2.8517583	4.2008765	1.000	-18.202356	12.498839
		SUGAR 113	-4.4355559	4.6967228	1.000	-21.598045	12.726934
		UBT (92) 25	1.3501349	4.2008765	1.000	-14.000462	16.700732
		VTTT 294/4-4	-3.4661152	4.4059165	1.000	-19.565957	12.633727
	NUA 746	A197	-.9219538	4.2008765	1.000	-16.272551	14.428643
		A286	1.2498410	4.4059165	1.000	-14.850001	17.349683
		A334	-.8423777	4.2008765	1.000	-16.192975	14.508219
		CAL113	-6.5347092	4.2008765	.990	-21.885306	8.815888
		CAL 143	-6.3857467	4.2008765	.992	-21.736344	8.964851
		DRK 57	-.4623276	4.2008765	1.000	-15.812925	14.888270

		NASAKA	1.0469077	4.4059165	1.000	-15.052935	17.146750
		NUA 45	-.5810113	4.2008765	1.000	-15.931609	14.769586
		NUA 59	-19.7398275*	4.6967228	.009	-36.902317	-2.577338
		NUA 674	1.8860720	5.1450020	1.000	-16.914493	20.686637
		NUA 705	-2.7967262	4.2008765	1.000	-18.147323	12.553871
		NUA 706	2.0527666	4.4059165	1.000	-14.047076	18.152609
		NUA 720	-.8899806	4.2008765	1.000	-16.240578	14.460617
		NUA 730	2.7425771	4.4059165	1.000	-13.357265	18.842419
		NUA 740	5.3794894	4.2008765	.999	-9.971108	20.730087
		NUA 743	2.8517583	4.2008765	1.000	-12.498839	18.202356
		SUGAR 113	-1.5837976	4.6967228	1.000	-18.746287	15.578692
		UBT (92) 25	4.2018931	4.2008765	1.000	-11.148704	19.552490
		VTTT 294/4-4	-.6143569	4.4059165	1.000	-16.714199	15.485485
	SUGAR 113	A197	.6618439	4.6967228	1.000	-16.500646	17.824333
		A286	2.8336386	4.8809775	1.000	-15.002144	20.669421
		A334	.7414199	4.6967228	1.000	-16.421070	17.903909
		CAL113	-4.9509116	4.6967228	1.000	-22.113401	12.211578
		CAL 143	-4.8019491	4.6967228	1.000	-21.964439	12.360540
		DRK 57	1.1214700	4.6967228	1.000	-16.041019	18.283959
		NASAKA	2.6307053	4.8809775	1.000	-15.205077	20.466487
		NUA 45	1.0027863	4.6967228	1.000	-16.159703	18.165276
		NUA 59	-18.1560299	5.1450020	.071	-36.956595	.644535
		NUA 674	3.4698696	5.5572373	1.000	-16.837062	23.776801
		NUA 705	-1.2129286	4.6967228	1.000	-18.375418	15.949561
		NUA 706	3.6365642	4.8809775	1.000	-14.199218	21.472346
		NUA 720	.6938170	4.6967228	1.000	-16.468672	17.856306
		NUA 730	4.3263747	4.8809775	1.000	-13.509407	22.162157
		NUA 740	6.9632870	4.6967228	.994	-10.199202	24.125776
		NUA 743	4.4355559	4.6967228	1.000	-12.726934	21.598045
		NUA 746	1.5837976	4.6967228	1.000	-15.578692	18.746287
		UBT (92) 25	5.7856908	4.6967228	.999	-11.376799	22.948180
		VTTT 294/4-4	.9694408	4.8809775	1.000	-16.866341	18.805223
	UBT (92) 25	A197	-5.1238469	4.2008765	1.000	-20.474444	10.226750
		A286	-2.9520522	4.4059165	1.000	-19.051894	13.147790
		A334	-5.0442709	4.2008765	1.000	-20.394868	10.306326
		CAL113	-10.7366023	4.2008765	.546	-26.087200	4.613995
		CAL 143	-10.5876399	4.2008765	.572	-25.938237	4.762957
		DRK 57	-4.6642207	4.2008765	1.000	-20.014818	10.686376
		NASAKA	-3.1549855	4.4059165	1.000	-19.254828	12.944857
		NUA 45	-4.7829045	4.2008765	1.000	-20.133502	10.567693

		NUA 59	-23.9417206*	4.6967228	.000	-41.104210	-6.779231
		NUA 674	-2.3158212	5.1450020	1.000	-21.116386	16.484744
		NUA 705	-6.9986194	4.2008765	.979	-22.349217	8.351978
		NUA 706	-2.1491265	4.4059165	1.000	-18.248969	13.950716
		NUA 720	-5.0918738	4.2008765	1.000	-20.442471	10.258723
		NUA 730	-1.4593160	4.4059165	1.000	-17.559158	14.640526
		NUA 740	1.1775963	4.2008765	1.000	-14.173001	16.528193
		NUA 743	-1.3501349	4.2008765	1.000	-16.700732	14.000462
		NUA 746	-4.2018931	4.2008765	1.000	-19.552490	11.148704
		SUGAR 113	-5.7856908	4.6967228	.999	-22.948180	11.376799
		VTTT 294/4-4	-4.8162500	4.4059165	1.000	-20.916092	11.283592
	VTTT 294/4-4	A197	-.3075969	4.4059165	1.000	-16.407439	15.792245
		A286	1.8641978	4.6018297	1.000	-14.951539	18.679935
		A334	-.2280209	4.4059165	1.000	-16.327863	15.871821
		CAL113	-5.9203523	4.4059165	.998	-22.020195	10.179490
		CAL 143	-5.7713898	4.4059165	.999	-21.871232	10.328452
		DRK 57	.1520293	4.4059165	1.000	-15.947813	16.251871
		NASAKA	1.6612645	4.6018297	1.000	-15.154472	18.477001
		NUA 45	.0333455	4.4059165	1.000	-16.066497	16.133188
		NUA 59	-19.1254706*	4.8809775	.023	-36.961253	-1.289688
		NUA 674	2.5004288	5.3137352	1.000	-16.916711	21.917569
		NUA 705	-2.1823694	4.4059165	1.000	-18.282212	13.917473
		NUA 706	2.6671235	4.6018297	1.000	-14.148613	19.482860
		NUA 720	-.2756238	4.4059165	1.000	-16.375466	15.824218
		NUA 730	3.3569340	4.6018297	1.000	-13.458803	20.172671
		NUA 740	5.9938463	4.4059165	.998	-10.105996	22.093688
		NUA 743	3.4661152	4.4059165	1.000	-12.633727	19.565957
		NUA 746	.6143569	4.4059165	1.000	-15.485485	16.714199
		SUGAR 113	-.9694408	4.8809775	1.000	-18.805223	16.866341
		UBT (92) 25	4.8162500	4.4059165	1.000	-11.283592	20.916092
Bonferroni	A197	A286	2.1717947	4.4059165	1.000	-14.584244	18.927834
		A334	.0795760	4.2008765	1.000	-15.896680	16.055832
		CAL113	-5.6127554	4.2008765	1.000	-21.589012	10.363501
		CAL 143	-5.4637929	4.2008765	1.000	-21.440049	10.512463
		DRK 57	.4596262	4.2008765	1.000	-15.516630	16.435883
		NASAKA	1.9688614	4.4059165	1.000	-14.787178	18.724900
		NUA 45	.3409424	4.2008765	1.000	-15.635314	16.317199
		NUA 59	-18.8178737*	4.6967228	.024	-36.679871	-.955876
		NUA 674	2.8080257	5.1450020	1.000	-16.758812	22.374864
		NUA 705	-1.8747725	4.2008765	1.000	-17.851029	14.101484

	NUA 706	2.9747204	4.4059165	1.000	-13.781319	19.730759
	NUA 720	.0319731	4.2008765	1.000	-15.944283	16.008229
	NUA 730	3.6645309	4.4059165	1.000	-13.091508	20.420570
	NUA 740	6.3014432	4.2008765	1.000	-9.674813	22.277700
	NUA 743	3.7737121	4.2008765	1.000	-12.202544	19.749968
	NUA 746	.9219538	4.2008765	1.000	-15.054303	16.898210
	SUGAR 113	-.6618439	4.6967228	1.000	-18.523841	17.200154
	UBT (92) 25	5.1238469	4.2008765	1.000	-10.852409	21.100103
	VTTT 294/4-4	.3075969	4.4059165	1.000	-16.448442	17.063636
A286	A197	-2.1717947	4.4059165	1.000	-18.927834	14.584244
	A334	-2.0922187	4.4059165	1.000	-18.848258	14.663820
	CAL113	-7.7845502	4.4059165	1.000	-24.540589	8.971489
	CAL 143	-7.6355877	4.4059165	1.000	-24.391627	9.120451
	DRK 57	-1.7121685	4.4059165	1.000	-18.468208	15.043871
	NASAKA	-.2029333	4.6018297	1.000	-17.704045	17.298179
	NUA 45	-1.8308523	4.4059165	1.000	-18.586891	14.925187
	NUA 59	-20.9896685*	4.8809775	.008	-39.552401	-2.426936
	NUA 674	.6362310	5.3137352	1.000	-19.572312	20.844774
	NUA 705	-4.0465672	4.4059165	1.000	-20.802606	12.709472
	NUA 706	.8029256	4.6018297	1.000	-16.698186	18.304038
	NUA 720	-2.1398216	4.4059165	1.000	-18.895861	14.616217
	NUA 730	1.4927362	4.6018297	1.000	-16.008376	18.993848
	NUA 740	4.1296484	4.4059165	1.000	-12.626391	20.885687
	NUA 743	1.6019173	4.4059165	1.000	-15.154122	18.357956
	NUA 746	-1.2498410	4.4059165	1.000	-18.005880	15.506198
	SUGAR 113	-2.8336386	4.8809775	1.000	-21.396371	15.729094
	UBT (92) 25	2.9520522	4.4059165	1.000	-13.803987	19.708091
	VTTT 294/4-4	-1.8641978	4.6018297	1.000	-19.365310	15.636914
A334	A197	-.0795760	4.2008765	1.000	-16.055832	15.896680
	A286	2.0922187	4.4059165	1.000	-14.663820	18.848258
	CAL113	-5.6923315	4.2008765	1.000	-21.668588	10.283925
	CAL 143	-5.5433690	4.2008765	1.000	-21.519625	10.432887
	DRK 57	.3800502	4.2008765	1.000	-15.596206	16.356307
	NASAKA	1.8892854	4.4059165	1.000	-14.866754	18.645324
	NUA 45	.2613664	4.2008765	1.000	-15.714890	16.237623
	NUA 59	-18.8974498*	4.6967228	.023	-36.759447	-1.035452
	NUA 674	2.7284497	5.1450020	1.000	-16.838388	22.295288
	NUA 705	-1.9543485	4.2008765	1.000	-17.930605	14.021908
	NUA 706	2.8951443	4.4059165	1.000	-13.860895	19.651183
	NUA 720	-.0476029	4.2008765	1.000	-16.023859	15.928653

		NUA 730	3.5849549	4.4059165	1.000	-13.171084	20.340994
		NUA 740	6.2218672	4.2008765	1.000	-9.754389	22.198124
		NUA 743	3.6941360	4.2008765	1.000	-12.282120	19.670392
		NUA 746	.8423777	4.2008765	1.000	-15.133879	16.818634
		SUGAR 113	-.7414199	4.6967228	1.000	-18.603418	17.120578
		UBT (92) 25	5.0442709	4.2008765	1.000	-10.931985	21.020527
		VTTT 294/4-4	.2280209	4.4059165	1.000	-16.528018	16.984060
	CAL113	A197	5.6127554	4.2008765	1.000	-10.363501	21.589012
		A286	7.7845502	4.4059165	1.000	-8.971489	24.540589
		A334	5.6923315	4.2008765	1.000	-10.283925	21.668588
		CAL 143	.1489625	4.2008765	1.000	-15.827294	16.125219
		DRK 57	6.0723816	4.2008765	1.000	-9.903875	22.048638
		NASAKA	7.5816168	4.4059165	1.000	-9.174422	24.337656
		NUA 45	5.9536978	4.2008765	1.000	-10.022559	21.929954
		NUA 59	-13.2051183	4.6967228	1.000	-31.067116	4.656879
		NUA 674	8.4207812	5.1450020	1.000	-11.146057	27.987619
		NUA 705	3.7379830	4.2008765	1.000	-12.238273	19.714239
		NUA 706	8.5874758	4.4059165	1.000	-8.168563	25.343515
		NUA 720	5.6447286	4.2008765	1.000	-10.331528	21.620985
		NUA 730	9.2772863	4.4059165	1.000	-7.478753	26.033325
		NUA 740	11.9141986	4.2008765	1.000	-4.062058	27.890455
		NUA 743	9.3864675	4.2008765	1.000	-6.589789	25.362724
		NUA 746	6.5347092	4.2008765	1.000	-9.441547	22.510966
		SUGAR 113	4.9509116	4.6967228	1.000	-12.911086	22.812909
		UBT (92) 25	10.7366023	4.2008765	1.000	-5.239654	26.712859
		VTTT 294/4-4	5.9203523	4.4059165	1.000	-10.835687	22.676391
	CAL 143	A197	5.4637929	4.2008765	1.000	-10.512463	21.440049
		A286	7.6355877	4.4059165	1.000	-9.120451	24.391627
		A334	5.5433690	4.2008765	1.000	-10.432887	21.519625
		CAL113	-.1489625	4.2008765	1.000	-16.125219	15.827294
		DRK 57	5.9234191	4.2008765	1.000	-10.052837	21.899676
		NASAKA	7.4326544	4.4059165	1.000	-9.323385	24.188693
		NUA 45	5.8047354	4.2008765	1.000	-10.171521	21.780992
		NUA 59	-13.3540808	4.6967228	1.000	-31.216078	4.507917
		NUA 674	8.2718187	5.1450020	1.000	-11.295019	27.838657
		NUA 705	3.5890205	4.2008765	1.000	-12.387236	19.565277
		NUA 706	8.4385133	4.4059165	1.000	-8.317526	25.194552
		NUA 720	5.4957661	4.2008765	1.000	-10.480490	21.472022
		NUA 730	9.1283238	4.4059165	1.000	-7.627715	25.884363
		NUA 740	11.7652361	4.2008765	1.000	-4.211020	27.741492

	NUA 743	9.2375050	4.2008765	1.000	-6.738751	25.213761
	NUA 746	6.3857467	4.2008765	1.000	-9.590510	22.362003
	SUGAR 113	4.8019491	4.6967228	1.000	-13.060049	22.663947
	UBT (92) 25	10.5876399	4.2008765	1.000	-5.388617	26.563896
	VTTT 294/4-4	5.7713898	4.4059165	1.000	-10.984649	22.527429
DRK 57	A197	-.4596262	4.2008765	1.000	-16.435883	15.516630
	A286	1.7121685	4.4059165	1.000	-15.043871	18.468208
	A334	-.3800502	4.2008765	1.000	-16.356307	15.596206
	CAL113	-6.0723816	4.2008765	1.000	-22.048638	9.903875
	CAL 143	-5.9234191	4.2008765	1.000	-21.899676	10.052837
	NASAKA	1.5092352	4.4059165	1.000	-15.246804	18.265274
	NUA 45	-.1186838	4.2008765	1.000	-16.094940	15.857573
	NUA 59	-19.2774999*	4.6967228	.017	-37.139498	-1.415502
	NUA 674	2.3483995	5.1450020	1.000	-17.218439	21.915238
	NUA 705	-2.3343986	4.2008765	1.000	-18.310655	13.641858
	NUA 706	2.5150942	4.4059165	1.000	-14.240945	19.271133
	NUA 720	-.4276531	4.2008765	1.000	-16.403909	15.548603
	NUA 730	3.2049047	4.4059165	1.000	-13.551134	19.960944
	NUA 740	5.8418170	4.2008765	1.000	-10.134439	21.818073
	NUA 743	3.3140859	4.2008765	1.000	-12.662171	19.290342
	NUA 746	.4623276	4.2008765	1.000	-15.513929	16.438584
	SUGAR 113	-1.1214700	4.6967228	1.000	-18.983468	16.740528
	UBT (92) 25	4.6642207	4.2008765	1.000	-11.312036	20.640477
	VTTT 294/4-4	-.1520293	4.4059165	1.000	-16.908068	16.604010
NASAKA	A197	-1.9688614	4.4059165	1.000	-18.724900	14.787178
	A286	.2029333	4.6018297	1.000	-17.298179	17.704045
	A334	-1.8892854	4.4059165	1.000	-18.645324	14.866754
	CAL113	-7.5816168	4.4059165	1.000	-24.337656	9.174422
	CAL 143	-7.4326544	4.4059165	1.000	-24.188693	9.323385
	DRK 57	-1.5092352	4.4059165	1.000	-18.265274	15.246804
	NUA 45	-1.6279190	4.4059165	1.000	-18.383958	15.128120
	NUA 59	-20.7867351*	4.8809775	.010	-39.349468	-2.224003
	NUA 674	.8391643	5.3137352	1.000	-19.369379	21.047708
	NUA 705	-3.8436339	4.4059165	1.000	-20.599673	12.912405
	NUA 706	1.0058590	4.6018297	1.000	-16.495253	18.506971
	NUA 720	-1.9368883	4.4059165	1.000	-18.692927	14.819151
	NUA 730	1.6956695	4.6018297	1.000	-15.805443	19.196781
	NUA 740	4.3325818	4.4059165	1.000	-12.423457	21.088621
	NUA 743	1.8048506	4.4059165	1.000	-14.951188	18.560890
	NUA 746	-1.0469077	4.4059165	1.000	-17.802947	15.709131

		SUGAR 113	-2.6307053	4.8809775	1.000	-21.193438	15.932027
		UBT (92) 25	3.1549855	4.4059165	1.000	-13.601054	19.911025
		VTTT 294/4-4	-1.6612645	4.6018297	1.000	-19.162377	15.839847
	NUA 45	A197	-.3409424	4.2008765	1.000	-16.317199	15.635314
		A286	1.8308523	4.4059165	1.000	-14.925187	18.586891
		A334	-.2613664	4.2008765	1.000	-16.237623	15.714890
		CAL113	-5.9536978	4.2008765	1.000	-21.929954	10.022559
		CAL 143	-5.8047354	4.2008765	1.000	-21.780992	10.171521
		DRK 57	.1186838	4.2008765	1.000	-15.857573	16.094940
		NASAKA	1.6279190	4.4059165	1.000	-15.128120	18.383958
		NUA 59	-19.1588162*	4.6967228	.019	-37.020814	-1.296819
		NUA 674	2.4670833	5.1450020	1.000	-17.099755	22.033921
		NUA 705	-2.2157149	4.2008765	1.000	-18.191971	13.760541
		NUA 706	2.6337779	4.4059165	1.000	-14.122261	19.389817
		NUA 720	-.3089693	4.2008765	1.000	-16.285226	15.667287
		NUA 730	3.3235885	4.4059165	1.000	-13.432451	20.079628
		NUA 740	5.9605008	4.2008765	1.000	-10.015756	21.936757
		NUA 743	3.4327696	4.2008765	1.000	-12.543487	19.409026
		NUA 746	.5810113	4.2008765	1.000	-15.395245	16.557268
		SUGAR 113	-1.0027863	4.6967228	1.000	-18.864784	16.859211
		UBT (92) 25	4.7829045	4.2008765	1.000	-11.193352	20.759161
		VTTT 294/4-4	-.0333455	4.4059165	1.000	-16.789385	16.722694
	NUA 59	A197	18.8178737*	4.6967228	.024	.955876	36.679871
		A286	20.9896685*	4.8809775	.008	2.426936	39.552401
		A334	18.8974498*	4.6967228	.023	1.035452	36.759447
		CAL113	13.2051183	4.6967228	1.000	-4.656879	31.067116
		CAL 143	13.3540808	4.6967228	1.000	-4.507917	31.216078
		DRK 57	19.2774999*	4.6967228	.017	1.415502	37.139498
		NASAKA	20.7867351*	4.8809775	.010	2.224003	39.349468
		NUA 45	19.1588162*	4.6967228	.019	1.296819	37.020814
		NUA 674	21.6258995*	5.5572373	.037	.491299	42.760500
		NUA 705	16.9431013	4.6967228	.097	-.918896	34.805099
		NUA 706	21.7925941*	4.8809775	.004	3.229862	40.355327
		NUA 720	18.8498469*	4.6967228	.024	.987849	36.711844
		NUA 730	22.4824046*	4.8809775	.003	3.919672	41.045137
		NUA 740	25.1193169*	4.6967228	.000	7.257319	42.981315
		NUA 743	22.5915858*	4.6967228	.001	4.729588	40.453583
		NUA 746	19.7398275*	4.6967228	.012	1.877830	37.601825
		SUGAR 113	18.1560299	5.1450020	.127	-1.410808	37.722868
		UBT (92) 25	23.9417206*	4.6967228	.000	6.079723	41.803718

	VTTT 294/4-4	19.1254706*	4.8809775	.033	.562738	37.688203
NUA 674	A197	-2.8080257	5.1450020	1.000	-22.374864	16.758812
	A286	-.6362310	5.3137352	1.000	-20.844774	19.572312
	A334	-2.7284497	5.1450020	1.000	-22.295288	16.838388
	CAL113	-8.4207812	5.1450020	1.000	-27.987619	11.146057
	CAL 143	-8.2718187	5.1450020	1.000	-27.838657	11.295019
	DRK 57	-2.3483995	5.1450020	1.000	-21.915238	17.218439
	NASAKA	-.8391643	5.3137352	1.000	-21.047708	19.369379
	NUA 45	-2.4670833	5.1450020	1.000	-22.033921	17.099755
	NUA 59	-21.6258995*	5.5572373	.037	-42.760500	-.491299
	NUA 705	-4.6827982	5.1450020	1.000	-24.249636	14.884040
	NUA 706	.1666946	5.3137352	1.000	-20.041849	20.375238
	NUA 720	-2.7760526	5.1450020	1.000	-22.342891	16.790785
	NUA 730	.8565052	5.3137352	1.000	-19.352038	21.065049
	NUA 740	3.4934175	5.1450020	1.000	-16.073421	23.060256
	NUA 743	.9656863	5.1450020	1.000	-18.601152	20.532524
	NUA 746	-1.8860720	5.1450020	1.000	-21.452910	17.680766
	SUGAR 113	-3.4698696	5.5572373	1.000	-24.604470	17.664731
	UBT (92) 25	2.3158212	5.1450020	1.000	-17.251017	21.882659
	VTTT 294/4-4	-2.5004288	5.3137352	1.000	-22.708972	17.708115
NUA 705	A197	1.8747725	4.2008765	1.000	-14.101484	17.851029
	A286	4.0465672	4.4059165	1.000	-12.709472	20.802606
	A334	1.9543485	4.2008765	1.000	-14.021908	17.930605
	CAL113	-3.7379830	4.2008765	1.000	-19.714239	12.238273
	CAL 143	-3.5890205	4.2008765	1.000	-19.565277	12.387236
	DRK 57	2.3343986	4.2008765	1.000	-13.641858	18.310655
	NASAKA	3.8436339	4.4059165	1.000	-12.912405	20.599673
	NUA 45	2.2157149	4.2008765	1.000	-13.760541	18.191971
	NUA 59	-16.9431013	4.6967228	.097	-34.805099	.918896
	NUA 674	4.6827982	5.1450020	1.000	-14.884040	24.249636
	NUA 706	4.8494928	4.4059165	1.000	-11.906546	21.605532
	NUA 720	1.9067456	4.2008765	1.000	-14.069511	17.883002
	NUA 730	5.5393033	4.4059165	1.000	-11.216736	22.295342
	NUA 740	8.1762156	4.2008765	1.000	-7.800041	24.152472
	NUA 743	5.6484845	4.2008765	1.000	-10.327772	21.624741
	NUA 746	2.7967262	4.2008765	1.000	-13.179530	18.772983
	SUGAR 113	1.2129286	4.6967228	1.000	-16.649069	19.074926
	UBT (92) 25	6.9986194	4.2008765	1.000	-8.977637	22.974876
	VTTT 294/4-4	2.1823694	4.4059165	1.000	-14.573670	18.938408
NUA 706	A197	-2.9747204	4.4059165	1.000	-19.730759	13.781319

		A286	-.8029256	4.6018297	1.000	-18.304038	16.698186
		A334	-2.8951443	4.4059165	1.000	-19.651183	13.860895
		CAL113	-8.5874758	4.4059165	1.000	-25.343515	8.168563
		CAL 143	-8.4385133	4.4059165	1.000	-25.194552	8.317526
		DRK 57	-2.5150942	4.4059165	1.000	-19.271133	14.240945
		NASAKA	-1.0058590	4.6018297	1.000	-18.506971	16.495253
		NUA 45	-2.6337779	4.4059165	1.000	-19.389817	14.122261
		NUA 59	-21.7925941*	4.8809775	.004	-40.355327	-3.229862
		NUA 674	-.1666946	5.3137352	1.000	-20.375238	20.041849
		NUA 705	-4.8494928	4.4059165	1.000	-21.605532	11.906546
		NUA 720	-2.9427472	4.4059165	1.000	-19.698786	13.813292
		NUA 730	.6898105	4.6018297	1.000	-16.811301	18.190923
		NUA 740	3.3267228	4.4059165	1.000	-13.429316	20.082762
		NUA 743	.7989917	4.4059165	1.000	-15.957047	17.555031
		NUA 746	-2.0527666	4.4059165	1.000	-18.808806	14.703272
		SUGAR 113	-3.6365642	4.8809775	1.000	-22.199297	14.926168
		UBT (92) 25	2.1491265	4.4059165	1.000	-14.606913	18.905166
		VTTT 294/4-4	-2.6671235	4.6018297	1.000	-20.168235	14.833989
	NUA 720	A197	-.0319731	4.2008765	1.000	-16.008229	15.944283
		A286	2.1398216	4.4059165	1.000	-14.616217	18.895861
		A334	.0476029	4.2008765	1.000	-15.928653	16.023859
		CAL113	-5.6447286	4.2008765	1.000	-21.620985	10.331528
		CAL 143	-5.4957661	4.2008765	1.000	-21.472022	10.480490
		DRK 57	.4276531	4.2008765	1.000	-15.548603	16.403909
		NASAKA	1.9368883	4.4059165	1.000	-14.819151	18.692927
		NUA 45	.3089693	4.2008765	1.000	-15.667287	16.285226
		NUA 59	-18.8498469*	4.6967228	.024	-36.711844	-.987849
		NUA 674	2.7760526	5.1450020	1.000	-16.790785	22.342891
		NUA 705	-1.9067456	4.2008765	1.000	-17.883002	14.069511
		NUA 706	2.9427472	4.4059165	1.000	-13.813292	19.698786
		NUA 730	3.6325578	4.4059165	1.000	-13.123481	20.388597
		NUA 740	6.2694701	4.2008765	1.000	-9.706786	22.245726
		NUA 743	3.7417389	4.2008765	1.000	-12.234517	19.717995
		NUA 746	.8899806	4.2008765	1.000	-15.086276	16.866237
		SUGAR 113	-.6938170	4.6967228	1.000	-18.555815	17.168181
		UBT (92) 25	5.0918738	4.2008765	1.000	-10.884383	21.068130
		VTTT 294/4-4	.2756238	4.4059165	1.000	-16.480415	17.031663
	NUA 730	A197	-3.6645309	4.4059165	1.000	-20.420570	13.091508
		A286	-1.4927362	4.6018297	1.000	-18.993848	16.008376
		A334	-3.5849549	4.4059165	1.000	-20.340994	13.171084

		CAL113	-9.2772863	4.4059165	1.000	-26.033325	7.478753
		CAL 143	-9.1283238	4.4059165	1.000	-25.884363	7.627715
		DRK 57	-3.2049047	4.4059165	1.000	-19.960944	13.551134
		NASAKA	-1.6956695	4.6018297	1.000	-19.196781	15.805443
		NUA 45	-3.3235885	4.4059165	1.000	-20.079628	13.432451
		NUA 59	-22.4824046*	4.8809775	.003	-41.045137	-3.919672
		NUA 674	-.8565052	5.3137352	1.000	-21.065049	19.352038
		NUA 705	-5.5393033	4.4059165	1.000	-22.295342	11.216736
		NUA 706	-.6898105	4.6018297	1.000	-18.190923	16.811301
		NUA 720	-3.6325578	4.4059165	1.000	-20.388597	13.123481
		NUA 740	2.6369123	4.4059165	1.000	-14.119127	19.392951
		NUA 743	.1091812	4.4059165	1.000	-16.646858	16.865220
		NUA 746	-2.7425771	4.4059165	1.000	-19.498616	14.013462
		SUGAR 113	-4.3263747	4.8809775	1.000	-22.889107	14.236358
		UBT (92) 25	1.4593160	4.4059165	1.000	-15.296723	18.215355
		VTTT 294/4-4	-3.3569340	4.6018297	1.000	-20.858046	14.144178
	NUA 740	A197	-6.3014432	4.2008765	1.000	-22.277700	9.674813
		A286	-4.1296484	4.4059165	1.000	-20.885687	12.626391
		A334	-6.2218672	4.2008765	1.000	-22.198124	9.754389
		CAL113	-11.9141986	4.2008765	1.000	-27.890455	4.062058
		CAL 143	-11.7652361	4.2008765	1.000	-27.741492	4.211020
		DRK 57	-5.8418170	4.2008765	1.000	-21.818073	10.134439
		NASAKA	-4.3325818	4.4059165	1.000	-21.088621	12.423457
		NUA 45	-5.9605008	4.2008765	1.000	-21.936757	10.015756
		NUA 59	-25.1193169*	4.6967228	.000	-42.981315	-7.257319
		NUA 674	-3.4934175	5.1450020	1.000	-23.060256	16.073421
		NUA 705	-8.1762156	4.2008765	1.000	-24.152472	7.800041
		NUA 706	-3.3267228	4.4059165	1.000	-20.082762	13.429316
		NUA 720	-6.2694701	4.2008765	1.000	-22.245726	9.706786
		NUA 730	-2.6369123	4.4059165	1.000	-19.392951	14.119127
		NUA 743	-2.5277311	4.2008765	1.000	-18.503987	13.448525
		NUA 746	-5.3794894	4.2008765	1.000	-21.355746	10.596767
		SUGAR 113	-6.9632870	4.6967228	1.000	-24.825285	10.898711
		UBT (92) 25	-1.1775963	4.2008765	1.000	-17.153853	14.798660
		VTTT 294/4-4	-5.9938463	4.4059165	1.000	-22.749885	10.762193
	NUA 743	A197	-3.7737121	4.2008765	1.000	-19.749968	12.202544
		A286	-1.6019173	4.4059165	1.000	-18.357956	15.154122
		A334	-3.6941360	4.2008765	1.000	-19.670392	12.282120
		CAL113	-9.3864675	4.2008765	1.000	-25.362724	6.589789
		CAL 143	-9.2375050	4.2008765	1.000	-25.213761	6.738751

		DRK 57	-3.3140859	4.2008765	1.000	-19.290342	12.662171
		NASAKA	-1.8048506	4.4059165	1.000	-18.560890	14.951188
		NUA 45	-3.4327696	4.2008765	1.000	-19.409026	12.543487
		NUA 59	-22.5915858*	4.6967228	.001	-40.453583	-4.729588
		NUA 674	-.9656863	5.1450020	1.000	-20.532524	18.601152
		NUA 705	-5.6484845	4.2008765	1.000	-21.624741	10.327772
		NUA 706	-.7989917	4.4059165	1.000	-17.555031	15.957047
		NUA 720	-3.7417389	4.2008765	1.000	-19.717995	12.234517
		NUA 730	-.1091812	4.4059165	1.000	-16.865220	16.646858
		NUA 740	2.5277311	4.2008765	1.000	-13.448525	18.503987
		NUA 746	-2.8517583	4.2008765	1.000	-18.828015	13.124498
		SUGAR 113	-4.4355559	4.6967228	1.000	-22.297554	13.426442
		UBT (92) 25	1.3501349	4.2008765	1.000	-14.626122	17.326391
		VTTT 294/4-4	-3.4661152	4.4059165	1.000	-20.222154	13.289924
	NUA 746	A197	-.9219538	4.2008765	1.000	-16.898210	15.054303
		A286	1.2498410	4.4059165	1.000	-15.506198	18.005880
		A334	-.8423777	4.2008765	1.000	-16.818634	15.133879
		CAL113	-6.5347092	4.2008765	1.000	-22.510966	9.441547
		CAL 143	-6.3857467	4.2008765	1.000	-22.362003	9.590510
		DRK 57	-.4623276	4.2008765	1.000	-16.438584	15.513929
		NASAKA	1.0469077	4.4059165	1.000	-15.709131	17.802947
		NUA 45	-.5810113	4.2008765	1.000	-16.557268	15.395245
		NUA 59	-19.7398275*	4.6967228	.012	-37.601825	-1.877830
		NUA 674	1.8860720	5.1450020	1.000	-17.680766	21.452910
		NUA 705	-2.7967262	4.2008765	1.000	-18.772983	13.179530
		NUA 706	2.0527666	4.4059165	1.000	-14.703272	18.808806
		NUA 720	-.8899806	4.2008765	1.000	-16.866237	15.086276
		NUA 730	2.7425771	4.4059165	1.000	-14.013462	19.498616
		NUA 740	5.3794894	4.2008765	1.000	-10.596767	21.355746
		NUA 743	2.8517583	4.2008765	1.000	-13.124498	18.828015
		SUGAR 113	-1.5837976	4.6967228	1.000	-19.445795	16.278200
		UBT (92) 25	4.2018931	4.2008765	1.000	-11.774363	20.178150
		VTTT 294/4-4	-.6143569	4.4059165	1.000	-17.370396	16.141682
	SUGAR 113	A197	.6618439	4.6967228	1.000	-17.200154	18.523841
		A286	2.8336386	4.8809775	1.000	-15.729094	21.396371
		A334	.7414199	4.6967228	1.000	-17.120578	18.603418
		CAL113	-4.9509116	4.6967228	1.000	-22.812909	12.911086
		CAL 143	-4.8019491	4.6967228	1.000	-22.663947	13.060049
		DRK 57	1.1214700	4.6967228	1.000	-16.740528	18.983468
		NASAKA	2.6307053	4.8809775	1.000	-15.932027	21.193438

		NUA 45	1.0027863	4.6967228	1.000	-16.859211	18.864784
		NUA 59	-18.1560299	5.1450020	.127	-37.722868	1.410808
		NUA 674	3.4698696	5.5572373	1.000	-17.664731	24.604470
		NUA 705	-1.2129286	4.6967228	1.000	-19.074926	16.649069
		NUA 706	3.6365642	4.8809775	1.000	-14.926168	22.199297
		NUA 720	.6938170	4.6967228	1.000	-17.168181	18.555815
		NUA 730	4.3263747	4.8809775	1.000	-14.236358	22.889107
		NUA 740	6.9632870	4.6967228	1.000	-10.898711	24.825285
		NUA 743	4.4355559	4.6967228	1.000	-13.426442	22.297554
		NUA 746	1.5837976	4.6967228	1.000	-16.278200	19.445795
		UBT (92) 25	5.7856908	4.6967228	1.000	-12.076307	23.647688
		VTTT 294/4-4	.9694408	4.8809775	1.000	-17.593292	19.532173
	UBT (92) 25	A197	-5.1238469	4.2008765	1.000	-21.100103	10.852409
		A286	-2.9520522	4.4059165	1.000	-19.708091	13.803987
		A334	-5.0442709	4.2008765	1.000	-21.020527	10.931985
		CAL113	-10.7366023	4.2008765	1.000	-26.712859	5.239654
		CAL 143	-10.5876399	4.2008765	1.000	-26.563896	5.388617
		DRK 57	-4.6642207	4.2008765	1.000	-20.640477	11.312036
		NASAKA	-3.1549855	4.4059165	1.000	-19.911025	13.601054
		NUA 45	-4.7829045	4.2008765	1.000	-20.759161	11.193352
		NUA 59	-23.9417206*	4.6967228	.000	-41.803718	-6.079723
		NUA 674	-2.3158212	5.1450020	1.000	-21.882659	17.251017
		NUA 705	-6.9986194	4.2008765	1.000	-22.974876	8.977637
		NUA 706	-2.1491265	4.4059165	1.000	-18.905166	14.606913
		NUA 720	-5.0918738	4.2008765	1.000	-21.068130	10.884383
		NUA 730	-1.4593160	4.4059165	1.000	-18.215355	15.296723
		NUA 740	1.1775963	4.2008765	1.000	-14.798660	17.153853
		NUA 743	-1.3501349	4.2008765	1.000	-17.326391	14.626122
		NUA 746	-4.2018931	4.2008765	1.000	-20.178150	11.774363
		SUGAR 113	-5.7856908	4.6967228	1.000	-23.647688	12.076307
		VTTT 294/4-4	-4.8162500	4.4059165	1.000	-21.572289	11.939789
	VTTT 294/4-4	A197	-.3075969	4.4059165	1.000	-17.063636	16.448442
		A286	1.8641978	4.6018297	1.000	-15.636914	19.365310
		A334	-.2280209	4.4059165	1.000	-16.984060	16.528018
		CAL113	-5.9203523	4.4059165	1.000	-22.676391	10.835687
		CAL 143	-5.7713898	4.4059165	1.000	-22.527429	10.984649
		DRK 57	.1520293	4.4059165	1.000	-16.604010	16.908068
		NASAKA	1.6612645	4.6018297	1.000	-15.839847	19.162377
		NUA 45	.0333455	4.4059165	1.000	-16.722694	16.789385
		NUA 59	-19.1254706*	4.8809775	.033	-37.688203	-.562738

	NUA 674	2.5004288	5.3137352	1.000	-17.708115	22.708972
	NUA 705	-2.1823694	4.4059165	1.000	-18.938408	14.573670
	NUA 706	2.6671235	4.6018297	1.000	-14.833989	20.168235
	NUA 720	-.2756238	4.4059165	1.000	-17.031663	16.480415
	NUA 730	3.3569340	4.6018297	1.000	-14.144178	20.858046
	NUA 740	5.9938463	4.4059165	1.000	-10.762193	22.749885
	NUA 743	3.4661152	4.4059165	1.000	-13.289924	20.222154
	NUA 746	.6143569	4.4059165	1.000	-16.141682	17.370396
	SUGAR 113	-.9694408	4.8809775	1.000	-19.532173	17.593292
	UBT (92) 25	4.8162500	4.4059165	1.000	-11.939789	21.572289

*. The mean difference is significant at the 0.05 level.

Homogeneous Subsets

Zn66_ppm

			Subset for alpha = 0.05	
	Variety	N	1	2
Tukey HSD[a,b]	NUA 740	6	32.455731	
	UBT (92) 25	6	33.633328	
	NUA 743	6	34.983463	
	NUA 730	5	35.092644	
	NUA 706	5	35.782454	
	NUA 674	3	35.949149	
	A286	5	36.585380	
	NASAKA	5	36.788313	
	NUA 746	6	37.835221	
	DRK 57	6	38.297548	
	NUA 45	6	38.416232	
	VTTT 294/4-4	5	38.449578	
	A334	6	38.677599	
	NUA 720	6	38.725201	
	A197	6	38.757175	
	SUGAR 113	4	39.419018	
	NUA 705	6	40.631947	
	CAL 143	6	44.220968	44.220968
	CAL113	6	44.369930	44.369930
	NUA 59	4		57.575048
	Sig.		.481	.275

Means for groups in homogeneous subsets are displayed.

a. Uses Harmonic Mean Sample Size = 5.217.

b. The group sizes are unequal. The harmonic mean of the group sizes is used. Type I error levels are not guaranteed.

```
USE ALL.
COMPUTE filter_$=(Condition = 1).
VARIABLE LABELS filter_$ 'Condition = 1 (FILTER)'.
VALUE LABELS filter_$ 0 'Not Selected' 1 'Selected'.
FORMATS filter_$ (f1.0).
FILTER BY filter_$.
EXECUTE.
ONEWAY LG10_Pb208 BY Variety
  /STATISTICS HOMOGENEITY
  /MISSING ANALYSIS
  /POSTHOC=TUKEY BONFERRONI ALPHA(0.05).
```

Oneway

Notes

Output Created		23-NOV-2017 18:36:09
Comments		
Input	Data	C:\Users\Brendan\Documents\Micronutrients\Yield and micronutirents variety+condition+season.sav
	Active Dataset	DataSet1
	Filter	Condition = 1 (FILTER)
	Weight	<none>
	Split File	<none>
	N of Rows in Working Data File	120
Missing Value Handling	Definition of Missing	User-defined missing values are treated as missing.
	Cases Used	Statistics for each analysis are based on cases with no missing data for any variable in the analysis.
Syntax		ONEWAY LG10_Pb208 BY Variety /STATISTICS HOMOGENEITY /MISSING ANALYSIS /POSTHOC=TUKEY BONFERRONI ALPHA(0.05).
Resources	Processor Time	00:00:00.09
	Elapsed Time	00:00:00.10

Test of Homogeneity of Variances

LG10_Pb208

Levene Statistic	df1	df2	Sig.
1.464	19	100	.116

ANOVA

LG10_Pb208

	Sum of Squares	df	Mean Square	F	Sig.
Between Groups	1.273	19	.067	.818	.682
Within Groups	8.195	100	.082		
Total	9.468	119			

```
USE ALL.
COMPUTE filter_$=(Condition = 2).
VARIABLE LABELS filter_$ 'Condition = 2 (FILTER)'.
VALUE LABELS filter_$ 0 'Not Selected' 1 'Selected'.
FORMATS filter_$ (f1.0).
FILTER BY filter_$.
EXECUTE.
ONEWAY LG10_Pb208 BY Variety
  /STATISTICS HOMOGENEITY
  /MISSING ANALYSIS
  /POSTHOC=TUKEY BONFERRONI ALPHA(0.05).
```

Oneway

Notes

Output Created		23-NOV-2017 18:37:21
Comments		
Input	Data	C:\Users\Brendan\Documents\Micronutrients\Yield and micronutirents variety+condition+season.sav
	Active Dataset	DataSet1
	Filter	Condition = 2 (FILTER)
	Weight	<none>
	Split File	<none>
	N of Rows in Working Data File	120
Missing Value Handling	Definition of Missing	User-defined missing values are treated as missing.

Cases Used		Statistics for each analysis are based on cases with no missing data for any variable in the analysis.
Syntax		ONEWAY LG10_Pb208 BY Variety<br>/STATISTICS HOMOGENEITY<br>/MISSING ANALYSIS<br>/POSTHOC=TUKEY BONFERRONI ALPHA(0.05).
Resources	Processor Time	00:00:00.14
	Elapsed Time	00:00:00.10

Test of Homogeneity of Variances

LG10_Pb208

Levene Statistic	df1	df2	Sig.
.469	19	90	.969

ANOVA

LG10_Pb208

	Sum of Squares	df	Mean Square	F	Sig.
Between Groups	1.402	19	.074	.195	1.000
Within Groups	34.068	90	.379		
Total	35.470	109			

```
USE ALL.
COMPUTE filter_$=(Condition = 1).
VARIABLE LABELS filter_$ 'Condition = 1 (FILTER)'.
VALUE LABELS filter_$ 0 'Not Selected' 1 'Selected'.
FORMATS filter_$ (f1.0).
FILTER BY filter_$.
EXECUTE.
ONEWAY Phytic_acid_percent BY Variety
  /STATISTICS HOMOGENEITY
  /MISSING ANALYSIS
  /POSTHOC=TUKEY BONFERRONI ALPHA(0.05).
```

Oneway

Test of Homogeneity of Variances

Phytic_acid_percent

Levene Statistic	df1	df2	Sig.
1.398	19	99	.146

ANOVA

Phytic_acid_percent

	Sum of Squares	df	Mean Square	F	Sig.
Between Groups	1.690	19	.089	.879	.609
Within Groups	10.020	99	.101		
Total	11.710	118			

```
USE ALL.
COMPUTE filter_$=(Condition = 2).
VARIABLE LABELS filter_$ 'Condition = 2 (FILTER)'.
VALUE LABELS filter_$ 0 'Not Selected' 1 'Selected'.
FORMATS filter_$ (f1.0).
FILTER BY filter_$.
EXECUTE.
ONEWAY Phytic_acid_percent BY Variety
  /STATISTICS HOMOGENEITY
  /MISSING ANALYSIS
  /POSTHOC=TUKEY BONFERRONI ALPHA(0.05).
```

Oneway

Notes

Output Created		23-NOV-2017 18:41:09
Comments		
Input	Data	C:\Users\Brendan\Documents\Micronutrients\Yield and micronutirents variety+condition+season.sav
	Active Dataset	DataSet1
	Filter	Condition = 2 (FILTER)
	Weight	<none>
	Split File	<none>
	N of Rows in Working Data File	120
Missing Value Handling	Definition of Missing	User-defined missing values are treated as missing.
	Cases Used	Statistics for each analysis are based on cases with no missing data for any variable in the analysis.

Syntax		ONEWAY Phytic_acid_percent BY Variety<br>/STATISTICS HOMOGENEITY<br>/MISSING ANALYSIS<br>/POSTHOC=TUKEY BONFERRONI ALPHA(0.05).
Resources	Processor Time	00:00:00.08
	Elapsed Time	00:00:00.10

Test of Homogeneity of Variances

Phytic_acid_percent

Levene Statistic	df1	df2	Sig.
1.515	19	90	.099

ANOVA

Phytic_acid_percent

	Sum of Squares	df	Mean Square	F	Sig.
Between Groups	2.041	19	.107	1.425	.135
Within Groups	6.784	90	.075		
Total	8.826	109			

```
USE ALL.
COMPUTE filter_$=(Condition = 1).
VARIABLE LABELS filter_$ 'Condition = 1 (FILTER)'.
VALUE LABELS filter_$ 0 'Not Selected' 1 'Selected'.
FORMATS filter_$ (f1.0).
FILTER BY filter_$.
EXECUTE.
ONEWAY Total_protein_mg_per_g_dw BY Variety
  /STATISTICS HOMOGENEITY
  /MISSING ANALYSIS
  /POSTHOC=TUKEY ALPHA(0.05).
```

Oneway

Notes

Output Created	05-JUN-2018 12:14:27
Comments	

Input	Data	C:\Users\Brendan\Documents\Micronutrients\Yield and micronutirents variety+condition+season.sav
	Active Dataset	DataSet1
	Filter	Condition = 1 (FILTER)
	Weight	<none>
	Split File	<none>
	N of Rows in Working Data File	120
Missing Value Handling	Definition of Missing	User-defined missing values are treated as missing.
	Cases Used	Statistics for each analysis are based on cases with no missing data for any variable in the analysis.
Syntax		ONEWAY Total_protein_mg_per_g_dw BY Variety /STATISTICS HOMOGENEITY /MISSING ANALYSIS /POSTHOC=TUKEY ALPHA(0.05).
Resources	Processor Time	00:00:00.13
	Elapsed Time	00:00:00.27

Test of Homogeneity of Variances

Total_protein_mg_per_g_dw

Levene Statistic	df1	df2	Sig.
.776	19	100	.729

ANOVA

Total_protein_mg_per_g_dw

	Sum of Squares	df	Mean Square	F	Sig.
Between Groups	87453.353	19	4602.808	.890	.596
Within Groups	517161.686	100	5171.617		
Total	604615.039	119			

```
USE ALL.
COMPUTE filter_$=(Condition = 2).
VARIABLE LABELS filter_$ 'Condition = 2 (FILTER)'.
VALUE LABELS filter_$ 0 'Not Selected' 1 'Selected'.
FORMATS filter_$ (f1.0).
FILTER BY filter_$.
EXECUTE.
ONEWAY Total_protein_mg_per_g_dw BY Variety
  /STATISTICS HOMOGENEITY
  /MISSING ANALYSIS
  /POSTHOC=TUKEY ALPHA(0.05).
```

Oneway

Notes

Output Created		05-JUN-2018 12:14:58
Comments		
Input	Data	C:\Users\Brendan\Documents\Micronutrients\Yield and micronutirents variety+condition+season.sav
	Active Dataset	DataSet1
	Filter	Condition = 2 (FILTER)
	Weight	<none>
	Split File	<none>
	N of Rows in Working Data File	120
Missing Value Handling	Definition of Missing	User-defined missing values are treated as missing.
	Cases Used	Statistics for each analysis are based on cases with no missing data for any variable in the analysis.
Syntax		ONEWAY Total_protein_mg_per_g_dw BY Variety /STATISTICS HOMOGENEITY /MISSING ANALYSIS /POSTHOC=TUKEY ALPHA(0.05).
Resources	Processor Time	00:00:00.06
	Elapsed Time	00:00:00.08

Test of Homogeneity of Variances

Total_protein_mg_per_g_dw

Levene Statistic	df1	df2	Sig.
1.962	19	90	.018

ANOVA

Total_protein_mg_per_g_dw

	Sum of Squares	df	Mean Square	F	Sig.
Between Groups	86000.983	19	4526.368	.770	.736
Within Groups	529360.208	90	5881.780		
Total	615361.191	109			

4) Two-tailed independent samples t-test statistical analysis

```
GET
  FILE='C:\Users\Brendan\Documents\Micronutrients\Yield and micronutirents
variety+condition+season.sav'.
DATASET NAME DataSet1 WINDOW=FRONT.
T-TEST GROUPS=Condition(1 2)
  /MISSING=ANALYSIS
  /VARIABLES=SQRT_Yield
  /CRITERIA=CI(.95).
```

T-Test

```
[DataSet1] C:\Users\Brendan\Documents\Micronutrients\Yield and
micronutirents variety+condition+season.sav
```

Group Statistics

	Condition	N	Mean	Std. Deviation	Std. Error Mean
SQRT_Yield	Rainfed	119	30.499820	6.9553653	.6375973
	Drought	117	17.005767	9.5655866	.8843388

Independent Samples Test

		Levene's Test for Equality of Variances		t-test for Equality of Means						
									95% Confidence Interval of the Difference	
		F	Sig.	t	df	Sig. (2-tailed)	Mean Difference	Std. Error Difference	Lower	Upper
SQRT_Yield	Equal variances assumed	15.801	.000	12.410	234	.000	13.4940526	1.0873658	11.3517749	15.6363303
	Equal variances not assumed			12.377	211.707	.000	13.4940526	1.0902226	11.3449701	15.6431351

```
T-TEST GROUPS=Condition(1 2)
  /MISSING=ANALYSIS
  /VARIABLES=Fe56_ppm
  /CRITERIA=CI(.95).
```

T-Test

Group Statistics

	Condition	N	Mean	Std. Deviation	Std. Error Mean
Fe56_ppm	Rainfed	120	58.192493	6.1808271	.5642297
	Drought	110	54.705894	9.0187895	.8599078

Independent Samples Test

		Levene's Test for Equality of Variances		t-test for Equality of Means						
									95% Confidence Interval of the Difference	
		F	Sig.	t	df	Sig. (2-tailed)	Mean Difference	Std. Error Difference	Lower	Upper
Fe56_ppm	Equal variances assumed	25.032	.000	3.444	228	.001	3.4865990	1.0124103	1.4917223	5.4814757
	Equal variances not assumed			3.390	190.686	.001	3.4865990	1.0284924	1.4579154	5.5152826

```
T-TEST GROUPS=Condition(1 2)
  /MISSING=ANALYSIS
  /VARIABLES=Zn66_ppm
  /CRITERIA=CI(.95).
```

T-Test

Group Statistics

	Condition	N	Mean	Std. Deviation	Std. Error Mean
Zn66_ppm	Rainfed	119	35.357169	7.8696258	.7214074
	Drought	108	38.660570	8.1642780	.7856080

Independent Samples Test

		Levene's Test for Equality of Variances		t-test for Equality of Means						
									95% Confidence Interval of the Difference	
		F	Sig.	t	df	Sig. (2-tailed)	Mean Difference	Std. Error Difference	Lower	Upper
Zn66_ppm	Equal variances assumed	.332	.565	-3.103	225	.002	-3.3034019	1.0646817	-5.4014246	-1.2053793
	Equal variances not assumed			-3.097	221.026	.002	-3.3034019	1.0665874	-5.4053844	-1.2014195

```
T-TEST GROUPS=Condition(1 2)
  /MISSING=ANALYSIS
  /VARIABLES=LG10_Pb208
  /CRITERIA=CI(.95).
```

T-Test

Group Statistics

	Condition	N	Mean	Std. Deviation	Std. Error Mean
LG10_Pb208	Rainfed	120	-1.5150	.28207	.02575
	Drought	110	-.9692	.57045	.05439

Independent Samples Test

		Levene's Test for Equality of Variances		t-test for Equality of Means						
									95% Confidence Interval of the Difference	
		F	Sig.	t	df	Sig. (2-tailed)	Mean Difference	Std. Error Difference	Lower	Upper
LG10_Pb208	Equal variances assumed	74.321	.000	-9.315	228	.000	-.54589	.05860	-.66136	-.43042
	Equal variances not assumed			-9.071	156.151	.000	-.54589	.06018	-.66475	-.42702

```
T-TEST GROUPS=Condition(1 2)
  /MISSING=ANALYSIS
  /VARIABLES=Phytic_acid_percent
  /CRITERIA=CI(.95).
```

T-Test

Group Statistics

	Condition	N	Mean	Std. Deviation	Std. Error Mean
Phytic_acid_percent	Rainfed	119	.9607	.31502	.02888
	Drought	110	1.1559	.28455	.02713

Independent Samples Test

		Levene's Test for Equality of Variances		t-test for Equality of Means						
									95% Confidence Interval of the Difference	
		F	Sig.	t	df	Sig. (2-tailed)	Mean Difference	Std. Error Difference	Lower	Upper
Phytic_acid_percent	Equal variances assumed	.675	.412	-4.907	227	.000	-.19519	.03978	-.27358	-.11680
	Equal variances not assumed			-4.926	226.883	.000	-.19519	.03962	-.27327	-.11711

```
T-TEST GROUPS=Condition(1 2)
  /MISSING=ANALYSIS
  /VARIABLES=Total_protein_mg_per_g_dw
  /CRITERIA=CI(.95).
```

T-Test

Notes

Output Created	05-JUN-2018 12:07:11
Comments	

Input	Data	C:\Users\Brendan\Documents\Micronutrients\Yield and micronutirents variety+condition+season.sav
	Active Dataset	DataSet1
	Filter	<none>
	Weight	<none>
	Split File	<none>
	N of Rows in Working Data File	240
Missing Value Handling	Definition of Missing	User defined missing values are treated as missing.
	Cases Used	Statistics for each analysis are based on the cases with no missing or out-of-range data for any variable in the analysis.
Syntax		T-TEST GROUPS=Condition(1 2) /MISSING=ANALYSIS /VARIABLES=Total_protein_mg_per_g_dw /CRITERIA=CI(.95).
Resources	Processor Time	00:00:00.02
	Elapsed Time	00:00:00.22

Group Statistics

	Condition	N	Mean	Std. Deviation	Std. Error Mean
Total_protein_mg_per_g_dw	Rainfed	120	325.5642	71.27972	6.50692
	Drought	110	370.9681	75.13665	7.16400

Independent Samples Test

		Levene's Test for Equality of Variances		t-test for Equality of Means							
										95% Confidence Interval of the Difference	
		F	Sig.	t	df	Sig. (2-tailed)	Mean Difference		Std. Error Difference	Lower	Upper
Total_protein_mg_per_g_dw	Equal variances assumed	.074	.786	-4.702	228	.000	-45.40393	9.65573		-64.42981	-26.37805
	Equal variances not assumed			-4.691	223.623	.000	-45.40393	9.67796		-64.47560	-26.33227

5) Multiple linear regression statistical analysis

```
REGRESSION
  /DESCRIPTIVES MEAN STDDEV CORR SIG N
  /MISSING LISTWISE
  /STATISTICS COEFF OUTS CI(95) R ANOVA COLLIN TOL
  /CRITERIA=PIN(.05) POUT(.10)
  /NOORIGIN
  /DEPENDENT SQRT_Yield
  /METHOD=STEPWISE Avg_Rain_Flowering_to_harvest Min_Temp_Flowering_to_harvest
    Max_Temp_Flowering_to_harvest Avg_Temp_Flowering_to_harvest
  /SCATTERPLOT=(*ZRESID ,*ZPRED)
  /RESIDUALS HISTOGRAM(ZRESID) NORMPROB(ZRESID)
  /CASEWISE PLOT(ZRESID) OUTLIERS(3)
  /SAVE PRED COOK ZRESID.
```

Regression

Notes

Output Created		22-NOV-2017 17:41:59
Comments		
Input	Data	C:\Users\Brendan\Documents\Micronutrients\Yield and micronutirents variety+condition+season.sav
	Active Dataset	DataSet1
	Filter	<none>
	Weight	<none>
	Split File	<none>
	N of Rows in Working Data File	240
Missing Value Handling	Definition of Missing	User-defined missing values are treated as missing.
	Cases Used	Statistics are based on cases with no missing values for any variable used.

Syntax		REGRESSION<br>/DESCRIPTIVES MEAN STDDEV CORR SIG N<br>/MISSING LISTWISE<br>/STATISTICS COEFF OUTS CI(95) R ANOVA COLLIN TOL<br>/CRITERIA=PIN(.05) POUT(.10)<br>/NOORIGIN<br>/DEPENDENT SQRT_Yield<br>/METHOD=STEPWISE Avg_Rain_Flowering_to_harvest Min_Temp_Flowering_to_harvest<br><br>Max_Temp_Flowering_to_harvest Avg_Temp_Flowering_to_harvest<br>/SCATTERPLOT=(*ZRESID ,*ZPRED)<br>/RESIDUALS HISTOGRAM(ZRESID) NORMPROB(ZRESID)<br>/CASEWISE PLOT(ZRESID) OUTLIERS(3)<br>/SAVE PRED COOK ZRESID.
Resources	Processor Time	00:00:00.48
	Elapsed Time	00:00:00.47
	Memory Required	6384 bytes
	Additional Memory Required for Residual Plots	632 bytes
Variables Created or Modified	PRE_2	Unstandardized Predicted Value
	ZRE_2	Standardized Residual
	COO_2	Cook's Distance

Descriptive Statistics

	Mean	Std. Deviation	N
SQRT_Yield	23.809972	10.7317498	236
Avg_Rain_Flowering_to_harvest	53.8276	34.36238	236
Min_Temp_Flowering_to_harvest	10.6373	1.26826	236
Max_Temp_Flowering_to_harvest	32.5750	2.55866	236
Avg_Temp_Flowering_to_harvest	22.6624	1.46309	236

Correlations

		SQRT_Yield	Avg_Rain_Flowering_to_harvest	Min_Temp_Flowering_to_harvest	Max_Temp_Flowering_to_harvest	Avg_Temp_Flowering_to_harvest
Pearson Correlation	SQRT_Yield	1.000	.118	-.619	-.661	-.743
	Avg_Rain_Flowering_to_harvest	.118	1.000	-.311	-.666	-.223
	Min_Temp_Flowering_to_harvest	-.619	-.311	1.000	.733	.847

	Max_Temp_Flowering_to_harvest	-.661	-.666	.733	1.000	.779
	Avg_Temp_Flowering_to_harvest	-.743	-.223	.847	.779	1.000
Sig. (1-tailed)	SQRT_Yield	.	.035	.000	.000	.000
	Avg_Rain_Flowering_to_harvest	.035	.	.000	.000	.000
	Min_Temp_Flowering_to_harvest	.000	.000	.	.000	.000
	Max_Temp_Flowering_to_harvest	.000	.000	.000	.	.000
	Avg_Temp_Flowering_to_harvest	.000	.000	.000	.000	.
N	SQRT_Yield	236	236	236	236	236
	Avg_Rain_Flowering_to_harvest	236	236	236	236	236
	Min_Temp_Flowering_to_harvest	236	236	236	236	236
	Max_Temp_Flowering_to_harvest	236	236	236	236	236
	Avg_Temp_Flowering_to_harvest	236	236	236	236	236

Variables Entered/Removed[a]

Model	Variables Entered	Variables Removed	Method
1	Avg_Temp_Flowering_to_harvest	.	Stepwise (Criteria: Probability-of-F-to-enter <= .050, Probability-of-F-to-remove >= .100).
2	Max_Temp_Flowering_to_harvest	.	Stepwise (Criteria: Probability-of-F-to-enter <= .050, Probability-of-F-to-remove >= .100).
3	Avg_Rain_Flowering_to_harvest	.	Stepwise (Criteria: Probability-of-F-to-enter <= .050, Probability-of-F-to-remove >= .100).

a. Dependent Variable: SQRT_Yield

Model Summary[d]

Model	R	R Square	Adjusted R Square	Std. Error of the Estimate
1	.743[a]	.553	.551	7.1926234
2	.755[b]	.570	.566	7.0686335
3	.798[c]	.637	.633	6.5040637

a. Predictors: (Constant), Avg_Temp_Flowering_to_harvest

b. Predictors: (Constant), Avg_Temp_Flowering_to_harvest, Max_Temp_Flowering_to_harvest

c. Predictors: (Constant), Avg_Temp_Flowering_to_harvest, Max_Temp_Flowering_to_harvest, Avg_Rain_Flowering_to_harvest

d. Dependent Variable: SQRT_Yield

ANOVA[a]

Model		Sum of Squares	df	Mean Square	F	Sig.
1	Regression	14959.340	1	14959.340	289.160	.000[b]
	Residual	12105.717	234	51.734		
	Total	27065.057	235			
2	Regression	15423.077	2	7711.538	154.337	.000[c]
	Residual	11641.980	233	49.966		
	Total	27065.057	235			
3	Regression	17250.797	3	5750.266	135.931	.000[d]
	Residual	9814.260	232	42.303		
	Total	27065.057	235			

a. Dependent Variable: SQRT_Yield

b. Predictors: (Constant), Avg_Temp_Flowering_to_harvest

c. Predictors: (Constant), Avg_Temp_Flowering_to_harvest, Max_Temp_Flowering_to_harvest

d. Predictors: (Constant), Avg_Temp_Flowering_to_harvest, Max_Temp_Flowering_to_harvest, Avg_Rain_Flowering_to_harvest

Coefficients[a]

Model		Unstandardized Coefficients		Standardized Coefficients	t	Sig.	95.0% Confidence Interval for B		Collinearity Statistics	
		B	Std. Error	Beta			Lower Bound	Upper Bound	Tolerance	VIF
1	(Constant)	147.393	7.283		20.239	.000	133.045	161.741		
	Avg_Temp_Flowering_to_harvest	-5.453	.321	-.743	-17.005	.000	-6.085	-4.821	1.000	1.000
2	(Constant)	148.868	7.173		20.753	.000	134.735	163.001		
	Avg_Temp_Flowering_to_harvest	-4.259	.503	-.581	-8.465	.000	-5.250	-3.267	.392	2.548
	Max_Temp_Flowering_to_harvest	-.876	.288	-.209	-3.046	.003	-1.443	-.310	.392	2.548
3	(Constant)	177.065	7.872		22.493	.000	161.555	192.574		
	Avg_Temp_Flowering_to_harvest	-1.777	.597	-.242	-2.975	.003	-2.954	-.600	.236	4.243
	Max_Temp_Flowering_to_harvest	-3.236	.446	-.772	-7.255	.000	-4.115	-2.358	.138	7.237
	Avg_Rain_Flowering_to_harvest	-.140	.021	-.449	-6.573	.000	-.182	-.098	.335	2.989

a. Dependent Variable: SQRT_Yield

Excluded Variables[a]

Model		Beta In	t	Sig.	Partial Correlation	Collinearity Statistics		
						Tolerance	VIF	Minimum Tolerance
1	Avg_Rain_Flowering_to_harvest	-.050[b]	-1.118	.265	-.073	.950	1.052	.950
	Min_Temp_Flowering_to_harvest	.040[b]	.483	.629	.032	.282	3.541	.282
	Max_Temp_Flowering_to_harvest	-.209[b]	-3.046	.003	-.196	.392	2.548	.392
2	Avg_Rain_Flowering_to_harvest	-.449[c]	-6.573	.000	-.396	.335	2.989	.138
	Min_Temp_Flowering_to_harvest	.098[c]	1.188	.236	.078	.269	3.719	.228
3	Min_Temp_Flowering_to_harvest	.047[d]	.615	.539	.040	.266	3.758	.138

a. Dependent Variable: SQRT_Yield

b. Predictors in the Model: (Constant), Avg_Temp_Flowering_to_harvest

c. Predictors in the Model: (Constant), Avg_Temp_Flowering_to_harvest, Max_Temp_Flowering_to_harvest

d. Predictors in the Model: (Constant), Avg_Temp_Flowering_to_harvest, Max_Temp_Flowering_to_harvest, Avg_Rain_Flowering_to_harvest

Collinearity Diagnostics[a]

Model	Dimension	Eigenvalue	Condition Index	Variance Proportions			
				(Constant)	Avg_Temp_Flowering_to_harvest	Max_Temp_Flowering_to_harvest	Avg_Rain_Flowering_to_harvest
1	1	1.998	1.000	.00	.00		
	2	.002	31.077	1.00	1.00		
2	1	2.996	1.000	.00	.00	.00	
	2	.003	30.688	.80	.02	.26	
	3	.001	53.689	.20	.98	.74	
3	1	3.748	1.000	.00	.00	.00	.01
	2	.249	3.877	.00	.00	.00	.30
	3	.002	41.781	.91	.11	.03	.10
	4	.000	88.671	.09	.89	.97	.59

a. Dependent Variable: SQRT_Yield

Residuals Statistics[a]

	Minimum	Maximum	Mean	Std. Deviation	N
Predicted Value	9.422302	34.096153	23.809972	8.5678262	236
Std. Predicted Value	-1.679	1.201	.000	1.000	236
Standard Error of Predicted Value	.737	2.110	.841	.099	236
Adjusted Predicted Value	9.356224	34.362850	23.810308	8.5684730	236
Residual	-17.7492199	16.9452763	.0000000	6.4624150	236
Std. Residual	-2.729	2.605	.000	.994	236
Stud. Residual	-2.749	2.625	.000	1.002	236

Deleted Residual	-18.0159168	17.2054539	-.0003362	6.5681072	236
Stud. Deleted Residual	-2.789	2.659	.000	1.007	236
Mahal. Distance	2.022	23.730	2.987	1.450	236
Cook's Distance	.000	.028	.004	.006	236
Centered Leverage Value	.009	.101	.013	.006	236

a. Dependent Variable: SQRT_Yield

Charts

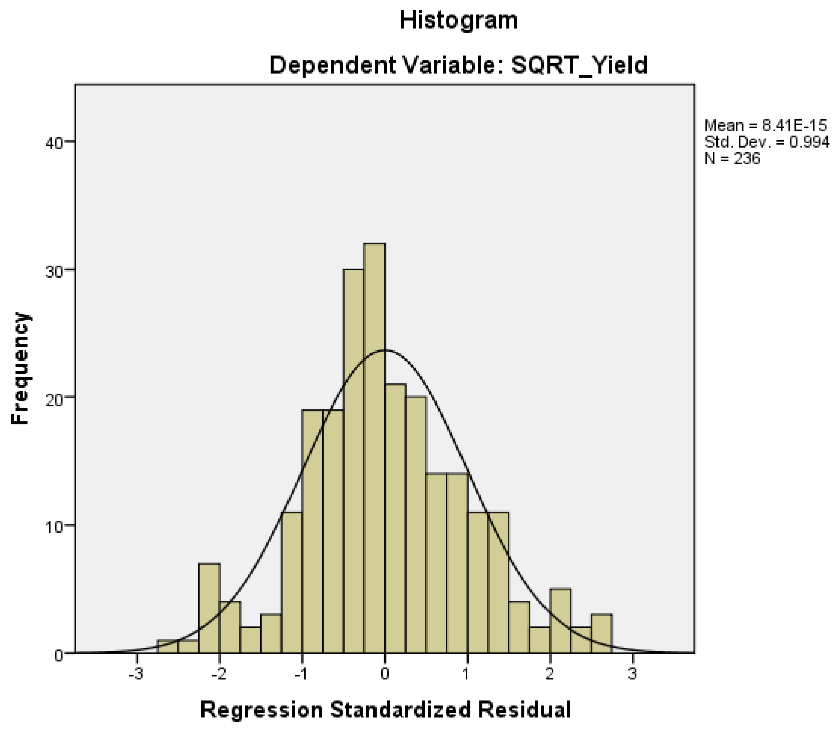

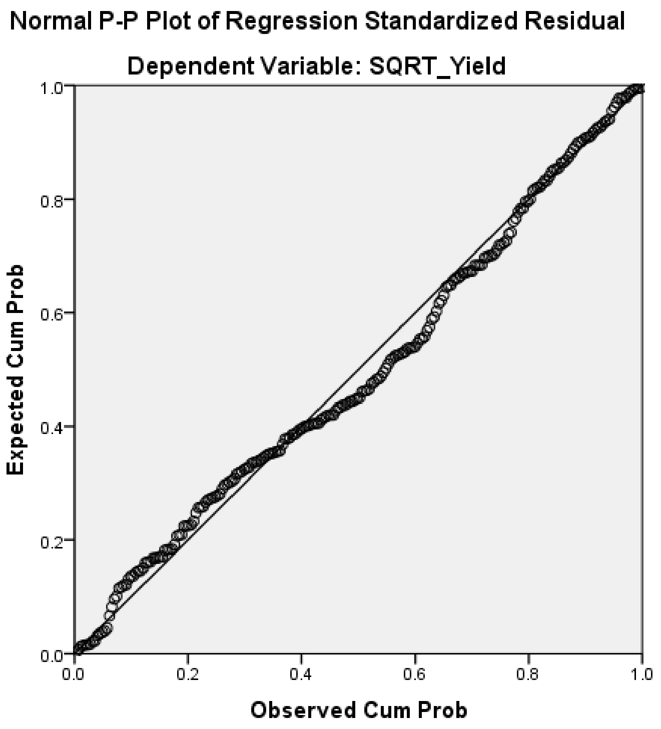
Normal P-P Plot of Regression Standardized Residual
Dependent Variable: SQRT_Yield
Expected Cum Prob
Observed Cum Prob
0.0
0.2
0.4
0.6
0.8
1.0

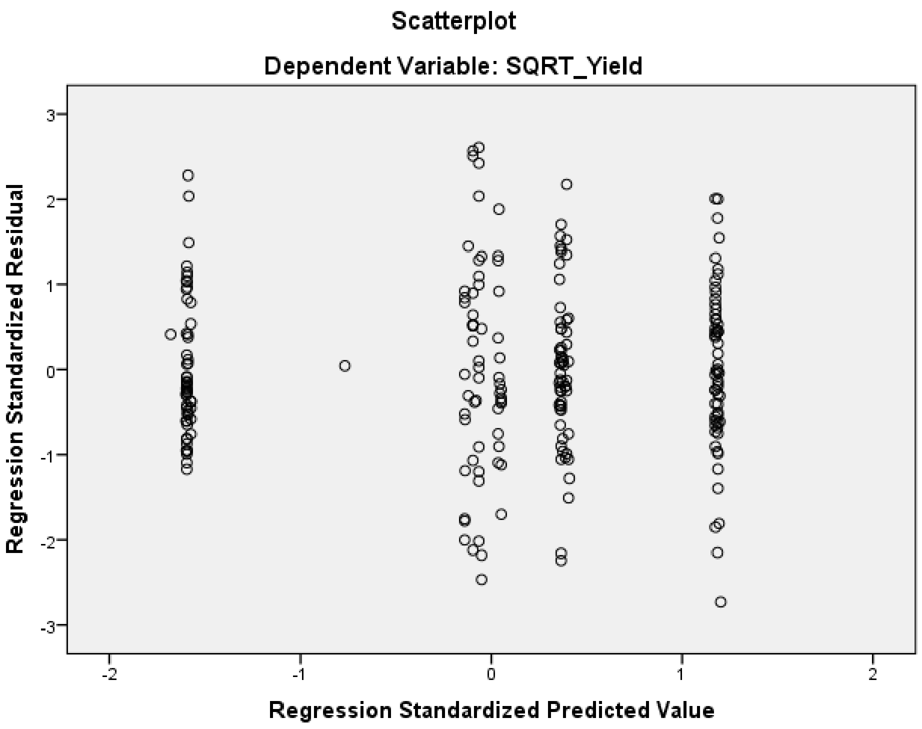
Scatterplot
Dependent Variable: SQRT_Yield
Regression Standardized Residual
Regression Standardized Predicted Value
3
2
1
0
-1
-2
-3
-2
-1
0
1
2

```
REGRESSION
  /DESCRIPTIVES MEAN STDDEV CORR SIG N
  /MISSING LISTWISE
  /STATISTICS COEFF OUTS CI(95) R ANOVA COLLIN TOL
  /CRITERIA=PIN(.05) POUT(.10)
  /NOORIGIN
  /DEPENDENT Fe56_ppm
  /METHOD=STEPWISE Avg_Rain_Flowering_to_harvest Min_Temp_Flowering_to_harvest
    Max_Temp_Flowering_to_harvest Avg_Temp_Flowering_to_harvest
  /SCATTERPLOT=(*ZRESID ,*ZPRED)
  /RESIDUALS HISTOGRAM(ZRESID) NORMPROB(ZRESID)
  /CASEWISE PLOT(ZRESID) OUTLIERS(3)
  /SAVE PRED COOK ZRESID.
```

Regression

Notes

Output Created		23-NOV-2017 16:00:30
Comments		
Input	Data	C:\Users\Brendan\Documents\Micronutrients\Yield and micronutirents variety+condition+season.sav
	Active Dataset	DataSet1
	Filter	<none>
	Weight	<none>
	Split File	<none>
	N of Rows in Working Data File	240
Missing Value Handling	Definition of Missing	User-defined missing values are treated as missing.

Cases Used	Statistics are based on cases with no missing values for any variable used.

Syntax		REGRESSION<br>/DESCRIPTIVES MEAN STDDEV CORR SIG N<br>/MISSING LISTWISE<br>/STATISTICS COEFF OUTS CI(95) R ANOVA COLLIN TOL<br>/CRITERIA=PIN(.05) POUT(.10)<br>/NOORIGIN<br>/DEPENDENT Fe56_ppm<br>/METHOD=STEPWISE Avg_Rain_Flowering_to_harvest Min_Temp_Flowering_to_harvest<br><br>Max_Temp_Flowering_to_harvest Avg_Temp_Flowering_to_harvest<br>/SCATTERPLOT=(*ZRESID ,*ZPRED)<br>/RESIDUALS HISTOGRAM(ZRESID) NORMPROB(ZRESID)<br>/CASEWISE PLOT(ZRESID) OUTLIERS(3)<br>/SAVE PRED COOK ZRESID.
Resources	Processor Time	00:00:00.95

	Elapsed Time	00:00:07.40
	Memory Required	6512 bytes
	Additional Memory Required for Residual Plots	632 bytes
Variables Created or Modified	PRE_3	Unstandardized Predicted Value
	ZRE_3	Standardized Residual
	COO_3	Cook's Distance

Descriptive Statistics

	Mean	Std. Deviation	N
Fe56_ppm	56.524989	7.8494808	230
Avg_Rain_Flowering_to_harvest	54.0339	34.95716	230
Min_Temp_Flowering_to_harvest	10.5826	1.25178	230
Max_Temp_Flowering_to_harvest	32.4783	2.54787	230
Avg_Temp_Flowering_to_harvest	22.5811	1.41461	230

Correlations

		Fe56_ppm	Avg_Rain_Flowering_to_harvest	Min_Temp_Flowering_to_harvest	Max_Temp_Flowering_to_harvest	Avg_Temp_Flowering_to_harvest
Pearson Correlation	Fe56_ppm	1.000	-.283	-.414	-.268	-.562
	Avg_Rain_Flowering_to_harvest	-.283	1.000	-.322	-.680	-.237
	Min_Temp_Flowering_to_harvest	-.414	-.322	1.000	.722	.839
	Max_Temp_Flowering_to_harvest	-.268	-.680	.722	1.000	.771
	Avg_Temp_Flowering_to_harvest	-.562	-.237	.839	.771	1.000
Sig. (1-tailed)	Fe56_ppm	.	.000	.000	.000	.000
	Avg_Rain_Flowering_to_harvest	.000	.	.000	.000	.000
	Min_Temp_Flowering_to_harvest	.000	.000	.	.000	.000
	Max_Temp_Flowering_to_harvest	.000	.000	.000	.	.000
	Avg_Temp_Flowering_to_harvest	.000	.000	.000	.000	.
N	Fe56_ppm	230	230	230	230	230
	Avg_Rain_Flowering_to_harvest	230	230	230	230	230
	Min_Temp_Flowering_to_harvest	230	230	230	230	230
	Max_Temp_Flowering_to_harvest	230	230	230	230	230
	Avg_Temp_Flowering_to_harvest	230	230	230	230	230

Variables Entered/Removed[a]

Model	Variables Entered	Variables Removed	Method
1	Avg_Temp_Flowering_to_harvest	.	Stepwise (Criteria: Probability-of-F-to-enter <= .050, Probability-of-F-to-remove >= .100).
2	Avg_Rain_Flowering_to_harvest	.	Stepwise (Criteria: Probability-of-F-to-enter <= .050, Probability-of-F-to-remove >= .100).
3	Max_Temp_Flowering_to_harvest	.	Stepwise (Criteria: Probability-of-F-to-enter <= .050, Probability-of-F-to-remove >= .100).

a. Dependent Variable: Fe56_ppm

Model Summary[d]

Model	R	R Square	Adjusted R Square	Std. Error of the Estimate
1	.562[a]	.316	.313	6.5063473
2	.707[b]	.500	.495	5.5756086
3	.721[c]	.520	.514	5.4730791

a. Predictors: (Constant), Avg_Temp_Flowering_to_harvest

b. Predictors: (Constant), Avg_Temp_Flowering_to_harvest, Avg_Rain_Flowering_to_harvest

c. Predictors: (Constant), Avg_Temp_Flowering_to_harvest, Avg_Rain_Flowering_to_harvest, Max_Temp_Flowering_to_harvest

d. Dependent Variable: Fe56_ppm

ANOVA[a]

Model		Sum of Squares	df	Mean Square	F	Sig.
1	Regression	4457.863	1	4457.863	105.306	.000[b]
	Residual	9651.823	228	42.333		
	Total	14109.686	229			
2	Regression	7052.843	2	3526.422	113.436	.000[c]
	Residual	7056.842	227	31.087		
	Total	14109.686	229			
3	Regression	7339.947	3	2446.649	81.679	.000[d]
	Residual	6769.738	226	29.955		
	Total	14109.686	229			

a. Dependent Variable: Fe56_ppm

b. Predictors: (Constant), Avg_Temp_Flowering_to_harvest

c. Predictors: (Constant), Avg_Temp_Flowering_to_harvest, Avg_Rain_Flowering_to_harvest

d. Predictors: (Constant), Avg_Temp_Flowering_to_harvest, Avg_Rain_Flowering_to_harvest, Max_Temp_Flowering_to_harvest

Coefficients[a]

Model		Unstandardized Coefficients B	Unstandardized Coefficients Std. Error	Standardized Coefficients Beta	t	Sig.	95.0% Confidence Interval for B Lower Bound	95.0% Confidence Interval for B Upper Bound	Collinearity Statistics Tolerance	Collinearity Statistics VIF
1	(Constant)	126.954	6.877		18.462	.000	113.404	140.504		
	Avg_Temp_Flowering_to_harvest	-3.119	.304	-.562	-10.262	.000	-3.718	-2.520	1.000	1.000
2	(Constant)	145.446	6.231		23.343	.000	133.168	157.723		
	Avg_Temp_Flowering_to_harvest	-3.701	.268	-.667	-13.802	.000	-4.229	-3.172	.944	1.060
	Avg_Rain_Flowering_to_harvest	-.099	.011	-.441	-9.136	.000	-.121	-.078	.944	1.060
3	(Constant)	155.037	6.856		22.613	.000	141.527	168.547		
	Avg_Temp_Flowering_to_harvest	-2.349	.510	-.423	-4.606	.000	-3.353	-1.344	.251	3.977
	Avg_Rain_Flowering_to_harvest	-.144	.018	-.640	-8.025	.000	-.179	-.108	.334	2.995
	Max_Temp_Flowering_to_harvest	-1.161	.375	-.377	-3.096	.002	-1.900	-.422	.143	6.981

a. Dependent Variable: Fe56_ppm

Excluded Variables[a]

Model		Beta In	t	Sig.	Partial Correlation	Collinearity Statistics		
						Tolerance	VIF	Minimum Tolerance
1	Avg_Rain_Flowering_to_harvest	-.441[b]	-9.136	.000	-.519	.944	1.060	.944
	Min_Temp_Flowering_to_harvest	.194[b]	1.939	.054	.128	.297	3.370	.297
	Max_Temp_Flowering_to_harvest	.408[b]	4.987	.000	.314	.405	2.470	.405
2	Min_Temp_Flowering_to_harvest	.011[c]	.123	.902	.008	.281	3.563	.281
	Max_Temp_Flowering_to_harvest	-.377[c]	-3.096	.002	-.202	.143	6.981	.143
3	Min_Temp_Flowering_to_harvest	.024[d]	.279	.780	.019	.280	3.572	.143

a. Dependent Variable: Fe56_ppm

b. Predictors in the Model: (Constant), Avg_Temp_Flowering_to_harvest

c. Predictors in the Model: (Constant), Avg_Temp_Flowering_to_harvest, Avg_Rain_Flowering_to_harvest

d. Predictors in the Model: (Constant), Avg_Temp_Flowering_to_harvest, Avg_Rain_Flowering_to_harvest, Max_Temp_Flowering_to_harvest

Collinearity Diagnostics[a]

Model	Dimension	Eigenvalue	Condition Index	Variance Proportions (Constant)	Avg_Temp_Flowering_to_harvest	Avg_Rain_Flowering_to_harvest	Max_Temp_Flowering_to_harvest
1	1	1.998	1.000	.00	.00		
	2	.002	32.026	1.00	1.00		
2	1	2.781	1.000	.00	.00	.03	
	2	.217	3.582	.00	.00	.89	
	3	.002	39.401	1.00	1.00	.08	
3	1	3.743	1.000	.00	.00	.01	.00
	2	.255	3.835	.00	.00	.30	.00
	3	.002	43.074	.93	.11	.11	.04
	4	.000	87.370	.07	.89	.58	.96

a. Dependent Variable: Fe56_ppm

Casewise Diagnostics[a]

Case Number	Std. Residual	Fe56_ppm	Predicted Value	Residual
32	-5.711	28.6682	59.927411	-31.2592485
174	-4.898	34.7061	61.512835	-26.8067682

a. Dependent Variable: Fe56_ppm

Residuals Statistics[a]

	Minimum	Maximum	Mean	Std. Deviation	N
Predicted Value	46.046883	62.088154	56.524989	5.6614637	230
Std. Predicted Value	-1.851	.983	.000	1.000	230
Standard Error of Predicted Value	.622	1.774	.716	.089	230
Adjusted Predicted Value	46.060757	62.224964	56.529872	5.6626074	230
Residual	-31.2592487	13.3050604	.0000000	5.4371110	230
Std. Residual	-5.711	2.431	.000	.993	230
Stud. Residual	-5.749	2.450	.000	1.002	230
Deleted Residual	-31.6687260	13.5093918	-.0048830	5.5358880	230
Stud. Deleted Residual	-6.208	2.477	-.004	1.023	230
Mahal. Distance	1.958	23.065	2.987	1.465	230
Cook's Distance	.000	.126	.005	.013	230
Centered Leverage Value	.009	.101	.013	.006	230

a. Dependent Variable: Fe56_ppm

Charts

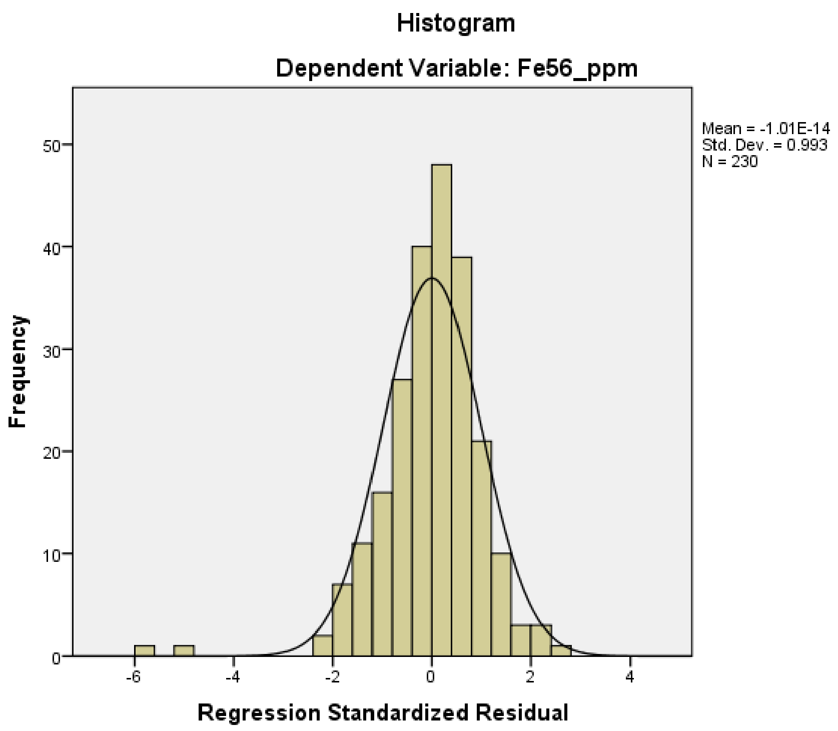

Histogram
Dependent Variable: Fe56_ppm
Mean = -1.01E-14
Std. Dev. = 0.993
N = 230
Frequency
50
40
30
20
10
0
-6
-4
-2
0
2
4
Regression Standardized Residual

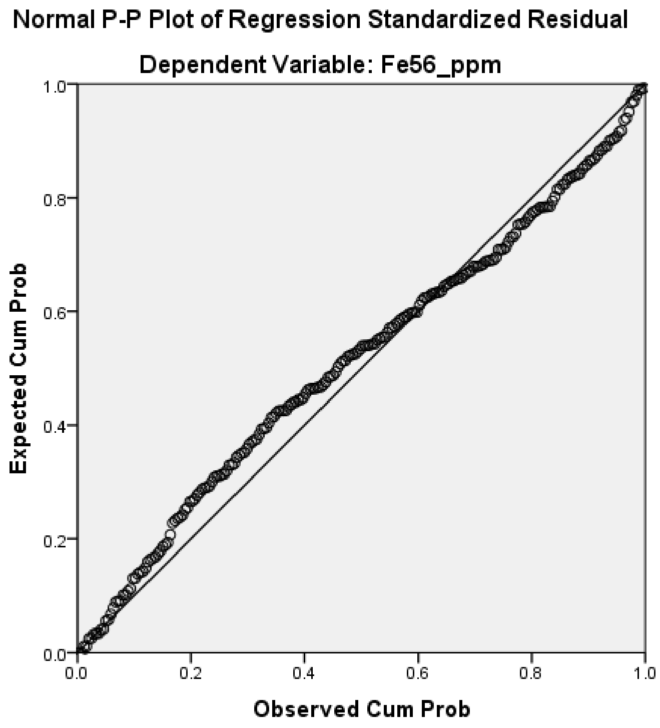

Normal P-P Plot of Regression Standardized Residual
Dependent Variable: Fe56_ppm
Expected Cum Prob
1.0
0.8
0.6
0.4
0.2
0.0
0.0
0.2
0.4
0.6
0.8
1.0
Observed Cum Prob

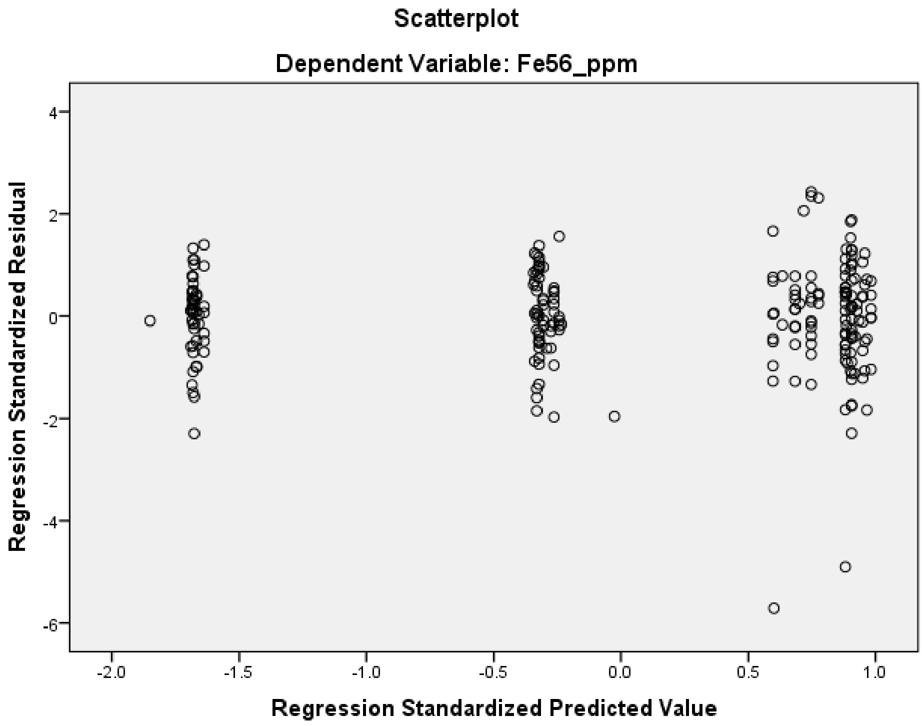

Scatterplot
Dependent Variable: Fe56_ppm
Regression Standardized Residual
Regression Standardized Predicted Value
4
2
0
-2
-4
-6
-2.0
-1.5
-1.0
-0.5
0.0
0.5
1.0

```
REGRESSION
  /DESCRIPTIVES MEAN STDDEV CORR SIG N
  /MISSING LISTWISE
  /STATISTICS COEFF OUTS CI(95) R ANOVA COLLIN TOL
  /CRITERIA=PIN(.05) POUT(.10)
  /NOORIGIN
  /DEPENDENT Zn66_ppm
  /METHOD=STEPWISE Avg_Rain_Flowering_to_harvest Min_Temp_Flowering_to_harvest
    Max_Temp_Flowering_to_harvest Avg_Temp_Flowering_to_harvest
  /SCATTERPLOT=(*ZRESID ,*ZPRED)
  /RESIDUALS HISTOGRAM(ZRESID) NORMPROB(ZRESID)
  /CASEWISE PLOT(ZRESID) OUTLIERS(3)
  /SAVE PRED COOK ZRESID.
```

Regression

Notes

Output Created		23-NOV-2017 17:20:10
Comments		
Input	Data	C:\Users\Brendan\Documents\Micronutrients\Yield and micronutirents variety+condition+season.sav
	Active Dataset	DataSet1
	Filter	<none>
	Weight	<none>
	Split File	<none>
	N of Rows in Working Data File	240
Missing Value Handling	Definition of Missing	User-defined missing values are treated as missing.
	Cases Used	Statistics are based on cases with no missing values for any variable used.

Syntax		REGRESSION<br>/DESCRIPTIVES MEAN STDDEV CORR SIG N<br>/MISSING LISTWISE<br>/STATISTICS COEFF OUTS CI(95) R ANOVA COLLIN TOL<br>/CRITERIA=PIN(.05) POUT(.10)<br>/NOORIGIN<br>/DEPENDENT Zn66_ppm<br>/METHOD=STEPWISE Avg_Rain_Flowering_to_harvest Min_Temp_Flowering_to_harvest<br><br>Max_Temp_Flowering_to_harves t Avg_Temp_Flowering_to_harves t<br>/SCATTERPLOT=(*ZRESID ,*ZPRED)<br>/RESIDUALS HISTOGRAM(ZRESID) NORMPROB(ZRESID)<br>/CASEWISE PLOT(ZRESID) OUTLIERS(3)<br>/SAVE PRED COOK ZRESID.
Resources	Processor Time	00:00:00.52
	Elapsed Time	00:00:00.85
	Memory Required	6624 bytes
	Additional Memory Required for Residual Plots	632 bytes
Variables Created or Modified	PRE_4	Unstandardized Predicted Value
	ZRE_4	Standardized Residual
	COO_4	Cook's Distance

Descriptive Statistics

	Mean	Std. Deviation	N
Zn66_ppm	36.928831	8.1625681	227
Avg_Rain_Flowering_to_harvest	54.2446	34.74610	227
Min_Temp_Flowering_to_harvest	10.5758	1.25345	227
Max_Temp_Flowering_to_harvest	32.4674	2.54966	227
Avg_Temp_Flowering_to_harvest	22.5844	1.42237	227

Correlations

		Zn66_ppm	Avg_Rain_Flowering_to_harvest	Min_Temp_Flowering_to_harvest	Max_Temp_Flowering_to_harvest	Avg_Temp_Flowering_to_harvest
Pearson Correlation	Zn66_ppm	1.000	-.130	.161	.201	.112
	Avg_Rain_Flowering_to_harvest	-.130	1.000	-.312	-.675	-.235
	Min_Temp_Flowering_to_harvest	.161	-.312	1.000	.719	.841
	Max_Temp_Flowering_to_harvest	.201	-.675	.719	1.000	.773
	Avg_Temp_Flowering_to_harvest	.112	-.235	.841	.773	1.000
Sig. (1-tailed)	Zn66_ppm	.	.025	.008	.001	.046
	Avg_Rain_Flowering_to_harvest	.025	.	.000	.000	.000
	Min_Temp_Flowering_to_harvest	.008	.000	.	.000	.000
	Max_Temp_Flowering_to_harvest	.001	.000	.000	.	.000
	Avg_Temp_Flowering_to_harvest	.046	.000	.000	.000	.
N	Zn66_ppm	227	227	227	227	227
	Avg_Rain_Flowering_to_harvest	227	227	227	227	227
	Min_Temp_Flowering_to_harvest	227	227	227	227	227
	Max_Temp_Flowering_to_harvest	227	227	227	227	227
	Avg_Temp_Flowering_to_harvest	227	227	227	227	227

Variables Entered/Removed[a]

Model	Variables Entered	Variables Removed	Method
1	Max_Temp_Flowering_to_harvest	.	Stepwise (Criteria: Probability-of-F-to-enter <= .050, Probability-of-F-to-remove >= .100).

a. Dependent Variable: Zn66_ppm

Model Summary[b]

Model	R	R Square	Adjusted R Square	Std. Error of the Estimate
1	.201[a]	.040	.036	8.0138276

a. Predictors: (Constant), Max_Temp_Flowering_to_harvest

b. Dependent Variable: Zn66_ppm

ANOVA[a]

Model		Sum of Squares	df	Mean Square	F	Sig.
1	Regression	607.996	1	607.996	9.467	.002[b]
	Residual	14449.822	225	64.221		
	Total	15057.819	226			

a. Dependent Variable: Zn66_ppm

b. Predictors: (Constant), Max_Temp_Flowering_to_harvest

Coefficients[a]

Model		Unstandardized Coefficients B	Unstandardized Coefficients Std. Error	Standardized Coefficients Beta	t	Sig.	95.0% Confidence Interval for B Lower Bound	95.0% Confidence Interval for B Upper Bound	Collinearity Statistics Tolerance	Collinearity Statistics VIF
1	(Constant)	16.043	6.809		2.356	.019	2.625	29.460		
	Max_Temp_Flowering_to_harvest	.643	.209	.201	3.077	.002	.231	1.055	1.000	1.000

a. Dependent Variable: Zn66_ppm

Excluded Variables[a]

Model		Beta In	t	Sig.	Partial Correlation	Collinearity Statistics Tolerance	Collinearity Statistics VIF	Collinearity Statistics Minimum Tolerance
1	Avg_Rain_Flowering_to_harvest	.010[b]	.114	.909	.008	.544	1.838	.544
	Min_Temp_Flowering_to_harvest	.034[b]	.358	.720	.024	.483	2.069	.483
	Avg_Temp_Flowering_to_harvest	-.107[b]	-1.042	.299	-.069	.402	2.488	.402

a. Dependent Variable: Zn66_ppm

b. Predictors in the Model: (Constant), Max_Temp_Flowering_to_harvest

Collinearity Diagnostics[a]

Model	Dimension	Eigenvalue	Condition Index	Variance Proportions (Constant)	Variance Proportions Max_Temp_Flowering_to_harvest
1	1	1.997	1.000	.00	.00
	2	.003	25.563	1.00	1.00

a. Dependent Variable: Zn66_ppm

Casewise Diagnostics[a]

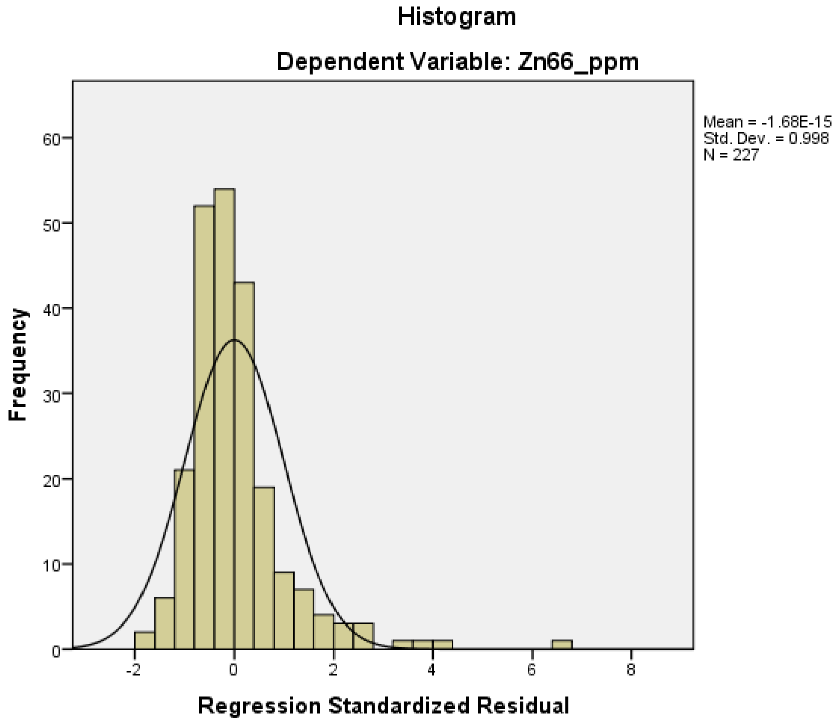

Case Number	Std. Residual	Zn66_ppm	Predicted Value	Residual
55	3.654	67.7776	38.493721	29.2838425
107	6.703	92.5357	38.815371	53.7203380
135	3.478	63.2790	35.405883	27.8731201
159	4.149	68.6578	35.405883	33.2518950

a. Dependent Variable: Zn66_ppm

Residuals Statistics[a]

	Minimum	Maximum	Mean	Std. Deviation	N
Predicted Value	35.341553	38.815372	36.928831	1.6401981	227
Std. Predicted Value	-.968	1.150	.000	1.000	227
Standard Error of Predicted Value	.727	.812	.752	.032	227
Adjusted Predicted Value	35.130287	38.954529	36.929096	1.6419774	227
Residual	-13.4254541	53.7203369	.0000000	7.9960783	227
Std. Residual	-1.675	6.703	.000	.998	227
Stud. Residual	-1.684	6.738	.000	1.002	227
Deleted Residual	-13.5646133	54.2771683	-.0002648	8.0678433	227
Stud. Deleted Residual	-1.691	7.525	.006	1.035	227
Mahal. Distance	.862	1.323	.996	.176	227
Cook's Distance	.000	.235	.004	.017	227
Centered Leverage Value	.004	.006	.004	.001	227

a. Dependent Variable: Zn66_ppm

Charts

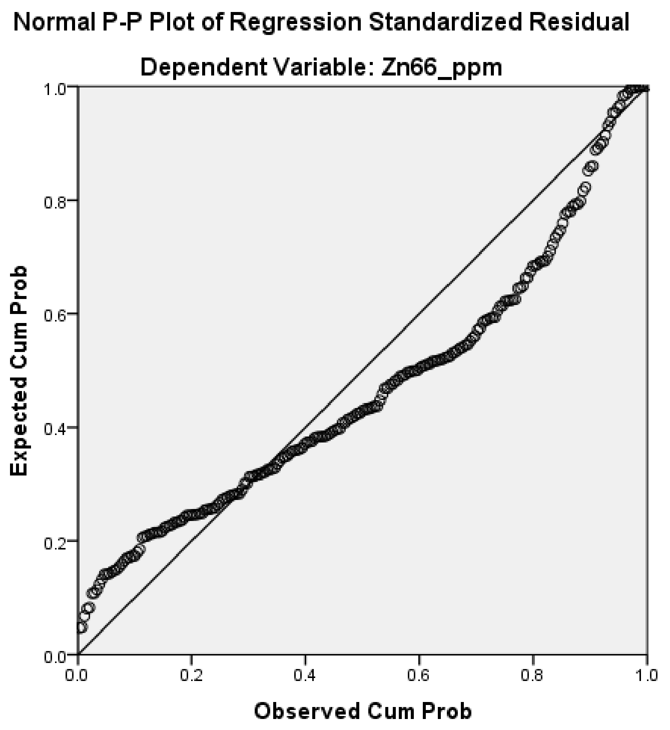
Normal P-P Plot of Regression Standardized Residual
Dependent Variable: Zn66_ppm
Expected Cum Prob
Observed Cum Prob
0.0
0.2
0.4
0.6
0.8
1.0

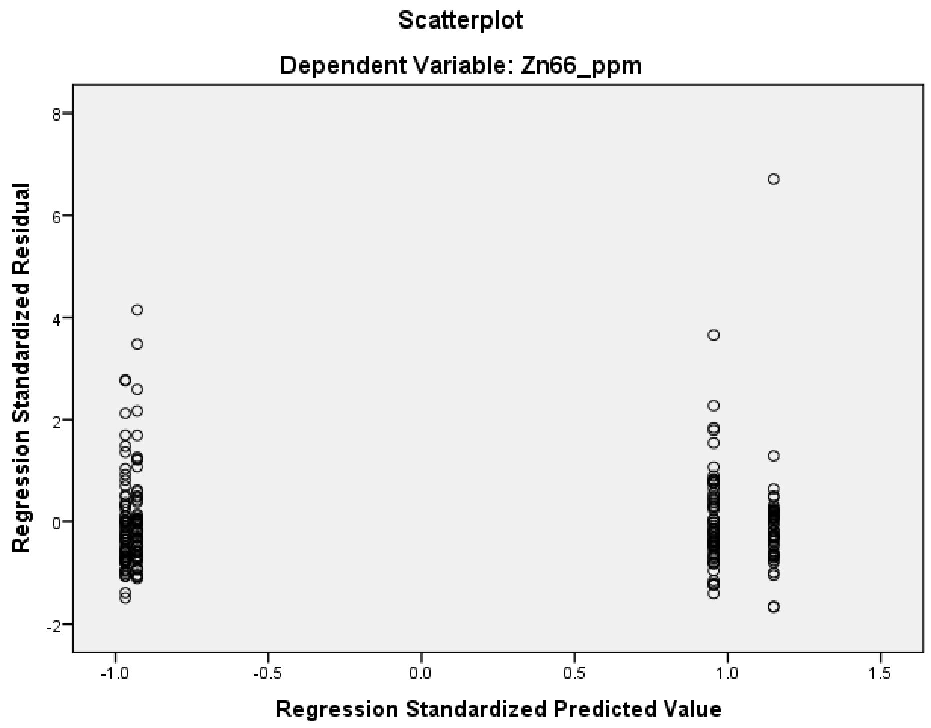
Scatterplot
Dependent Variable: Zn66_ppm
Regression Standardized Residual
Regression Standardized Predicted Value
8
6
4
2
0
-2
-1.0
-0.5
0.0
0.5
1.0
1.5

```
REGRESSION
  /DESCRIPTIVES MEAN STDDEV CORR SIG N
  /MISSING LISTWISE
  /STATISTICS COEFF OUTS CI(95) R ANOVA COLLIN TOL
  /CRITERIA=PIN(.05) POUT(.10)
  /NOORIGIN
  /DEPENDENT LG10_Pb208
  /METHOD=STEPWISE Avg_Rain_Flowering_to_harvest Min_Temp_Flowering_to_harvest
    Max_Temp_Flowering_to_harvest Avg_Temp_Flowering_to_harvest
  /SCATTERPLOT=(*ZRESID ,*ZPRED)
  /RESIDUALS HISTOGRAM(ZRESID) NORMPROB(ZRESID)
  /CASEWISE PLOT(ZRESID) OUTLIERS(3)
  /SAVE PRED COOK ZRESID.
```

Regression

Notes

Output Created		23-NOV-2017 17:28:22
Comments		
Input	Data	C:\Users\Brendan\Documents\Micronutrients\Yield and micronutirents variety+condition+season.sav
	Active Dataset	DataSet1
	Filter	<none>
	Weight	<none>
	Split File	<none>
	N of Rows in Working Data File	240
Missing Value Handling	Definition of Missing	User-defined missing values are treated as missing.
	Cases Used	Statistics are based on cases with no missing values for any variable used.

Syntax		REGRESSION<br>/DESCRIPTIVES MEAN STDDEV CORR SIG N<br>/MISSING LISTWISE<br>/STATISTICS COEFF OUTS CI(95) R ANOVA COLLIN TOL<br>/CRITERIA=PIN(.05) POUT(.10)<br>/NOORIGIN<br>/DEPENDENT LG10_Pb208<br>/METHOD=STEPWISE Avg_Rain_Flowering_to_harvest Min_Temp_Flowering_to_harvest<br><br>Max_Temp_Flowering_to_harvest Avg_Temp_Flowering_to_harvest<br>/SCATTERPLOT=(*ZRESID ,*ZPRED)<br>/RESIDUALS HISTOGRAM(ZRESID) NORMPROB(ZRESID)<br>/CASEWISE PLOT(ZRESID) OUTLIERS(3)<br>/SAVE PRED COOK ZRESID.
Resources	Processor Time	00:00:00.36
	Elapsed Time	00:00:00.46
	Memory Required	6752 bytes
	Additional Memory Required for Residual Plots	632 bytes
Variables Created or Modified	PRE_5	Unstandardized Predicted Value
	ZRE_5	Standardized Residual
	COO_5	Cook's Distance

Descriptive Statistics

	Mean	Std. Deviation	N
LG10_Pb208	-1.2540	.52050	230
Avg_Rain_Flowering_to_harvest	54.0339	34.95716	230
Min_Temp_Flowering_to_harvest	10.5826	1.25178	230
Max_Temp_Flowering_to_harvest	32.4783	2.54787	230
Avg_Temp_Flowering_to_harvest	22.5811	1.41461	230

Correlations

		LG10_Pb208	Avg_Rain_Flowering_to_harvest	Min_Temp_Flowering_to_harvest	Max_Temp_Flowering_to_harvest	Avg_Temp_Flowering_to_harvest
Pearson Correlation	LG10_Pb208	1.000	-.227	.483	.548	.612
	Avg_Rain_Flowering_to_harvest	-.227	1.000	-.322	-.680	-.237
	Min_Temp_Flowering_to_harvest	.483	-.322	1.000	.722	.839
	Max_Temp_Flowering_to_harvest	.548	-.680	.722	1.000	.771
	Avg_Temp_Flowering_to_harvest	.612	-.237	.839	.771	1.000
Sig. (1-tailed)	LG10_Pb208	.	.000	.000	.000	.000
	Avg_Rain_Flowering_to_harvest	.000	.	.000	.000	.000
	Min_Temp_Flowering_to_harvest	.000	.000	.	.000	.000
	Max_Temp_Flowering_to_harvest	.000	.000	.000	.	.000
	Avg_Temp_Flowering_to_harvest	.000	.000	.000	.000	.
N	LG10_Pb208	230	230	230	230	230
	Avg_Rain_Flowering_to_harvest	230	230	230	230	230
	Min_Temp_Flowering_to_harvest	230	230	230	230	230
	Max_Temp_Flowering_to_harvest	230	230	230	230	230
	Avg_Temp_Flowering_to_harvest	230	230	230	230	230

Variables Entered/Removed[a]

Model	Variables Entered	Variables Removed	Method
1	Avg_Temp_Flowering_to_harvest	.	Stepwise (Criteria: Probability-of-F-to-enter <= .050, Probability-of-F-to-remove >= .100).
2	Max_Temp_Flowering_to_harvest	.	Stepwise (Criteria: Probability-of-F-to-enter <= .050, Probability-of-F-to-remove >= .100).

a. Dependent Variable: LG10_Pb208

Model Summary[c]

Model	R	R Square	Adjusted R Square	Std. Error of the Estimate
1	.612[a]	.375	.372	.41249
2	.624[b]	.389	.384	.40868

a. Predictors: (Constant), Avg_Temp_Flowering_to_harvest

b. Predictors: (Constant), Avg_Temp_Flowering_to_harvest, Max_Temp_Flowering_to_harvest

c. Dependent Variable: LG10_Pb208

ANOVA[a]

Model		Sum of Squares	df	Mean Square	F	Sig.
1	Regression	23.246	1	23.246	136.619	.000[b]
	Residual	38.794	228	.170		
	Total	62.040	229			
2	Regression	24.127	2	12.064	72.229	.000[c]
	Residual	37.913	227	.167		
	Total	62.040	229			

a. Dependent Variable: LG10_Pb208

b. Predictors: (Constant), Avg_Temp_Flowering_to_harvest

c. Predictors: (Constant), Avg_Temp_Flowering_to_harvest, Max_Temp_Flowering_to_harvest

Coefficients[a]

Model		Unstandardized Coefficients B	Unstandardized Coefficients Std. Error	Standardized Coefficients Beta	t	Sig.	95.0% Confidence Interval for B Lower Bound	95.0% Confidence Interval for B Upper Bound	Collinearity Statistics Tolerance	Collinearity Statistics VIF
1	(Constant)	-6.340	.436		-14.542	.000	-7.199	-5.481		
	Avg_Temp_Flowering_to_harvest	.225	.019	.612	11.688	.000	.187	.263	1.000	1.000
2	(Constant)	-6.382	.432		-14.762	.000	-7.234	-5.530		
	Avg_Temp_Flowering_to_harvest	.172	.030	.468	5.734	.000	.113	.231	.405	2.470
	Max_Temp_Flowering_to_harvest	.038	.017	.187	2.297	.023	.005	.071	.405	2.470

a. Dependent Variable: LG10_Pb208

Excluded Variables[a]

Model		Beta In	t	Sig.	Partial Correlation	Collinearity Statistics Tolerance	Collinearity Statistics VIF	Collinearity Statistics Minimum Tolerance
1	Avg_Rain_Flowering_to_harvest	-.087[b]	-1.613	.108	-.106	.944	1.060	.944
	Min_Temp_Flowering_to_harvest	-.104[b]	-1.080	.282	-.071	.297	3.370	.297
	Max_Temp_Flowering_to_harvest	.187[b]	2.297	.023	.151	.405	2.470	.405
2	Avg_Rain_Flowering_to_harvest	.034[c]	.376	.708	.025	.334	2.995	.143
	Min_Temp_Flowering_to_harvest	-.158[c]	-1.630	.105	-.108	.283	3.535	.239

a. Dependent Variable: LG10_Pb208

b. Predictors in the Model: (Constant), Avg_Temp_Flowering_to_harvest

c. Predictors in the Model: (Constant), Avg_Temp_Flowering_to_harvest, Max_Temp_Flowering_to_harvest

Collinearity Diagnostics[a]

Model	Dimension	Eigenvalue	Condition Index	Variance Proportions (Constant)	Avg_Temp_Flowering_to_harvest	Max_Temp_Flowering_to_harvest
1	1	1.998	1.000	.00	.00	
	2	.002	32.026	1.00	1.00	
2	1	2.996	1.000	.00	.00	.00
	2	.003	30.922	.74	.01	.29
	3	.001	54.132	.26	.99	.71

a. Dependent Variable: LG10_Pb208

Casewise Diagnostics[a]

Case Number	Std. Residual	LG10_Pb208	Predicted Value	Residual
15	3.823	.02	-1.5430	1.56256

a. Dependent Variable: LG10_Pb208

Residuals Statistics[a]

	Minimum	Maximum	Mean	Std. Deviation	N
Predicted Value	-1.5551	-.6228	-1.2540	.32459	230
Std. Predicted Value	-.928	1.945	.000	1.000	230
Standard Error of Predicted Value	.037	.089	.046	.010	230
Adjusted Predicted Value	-1.5564	-.6258	-1.2543	.32459	230
Residual	-.90901	1.56256	.00000	.40689	230
Std. Residual	-2.224	3.823	.000	.996	230
Stud. Residual	-2.237	3.839	.000	1.003	230
Deleted Residual	-.91926	1.57555	.00028	.41322	230
Stud. Deleted Residual	-2.257	3.962	.002	1.009	230
Mahal. Distance	.871	9.870	1.991	1.344	230
Cook's Distance	.000	.045	.005	.008	230
Centered Leverage Value	.004	.043	.009	.006	230

a. Dependent Variable: LG10_Pb208

Charts

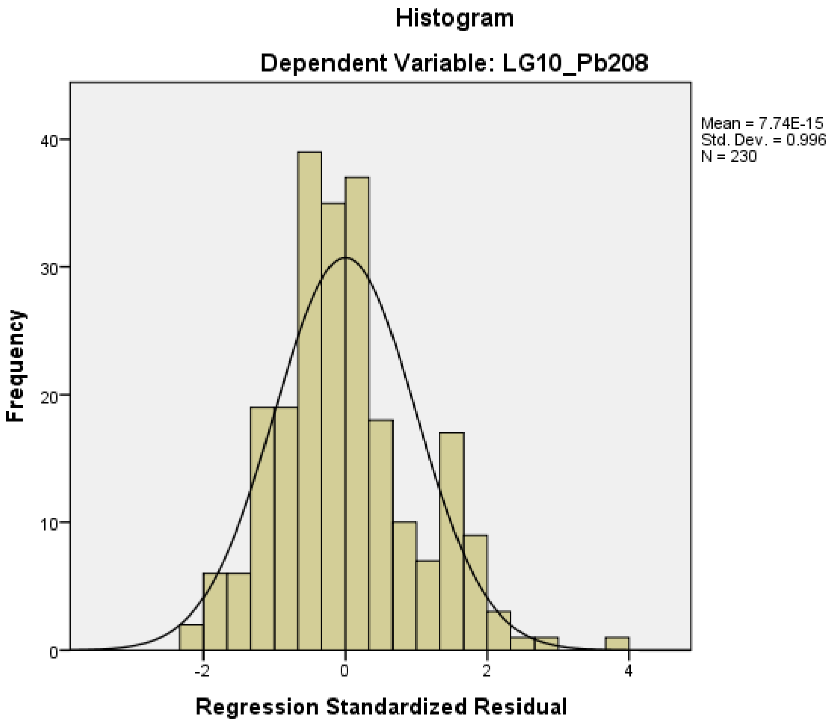

Histogram
Dependent Variable: LG10_Pb208
Mean = 7.74E-15
Std. Dev. = 0.996
N = 230
Frequency
0
10
20
30
40
-2
0
2
4
Regression Standardized Residual

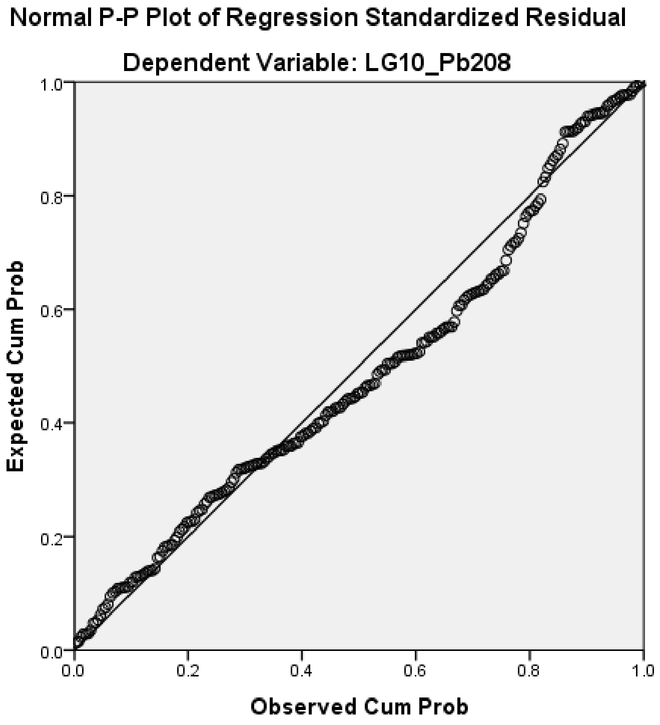

Normal P-P Plot of Regression Standardized Residual
Dependent Variable: LG10_Pb208
Expected Cum Prob
0.0
0.2
0.4
0.6
0.8
1.0
Observed Cum Prob

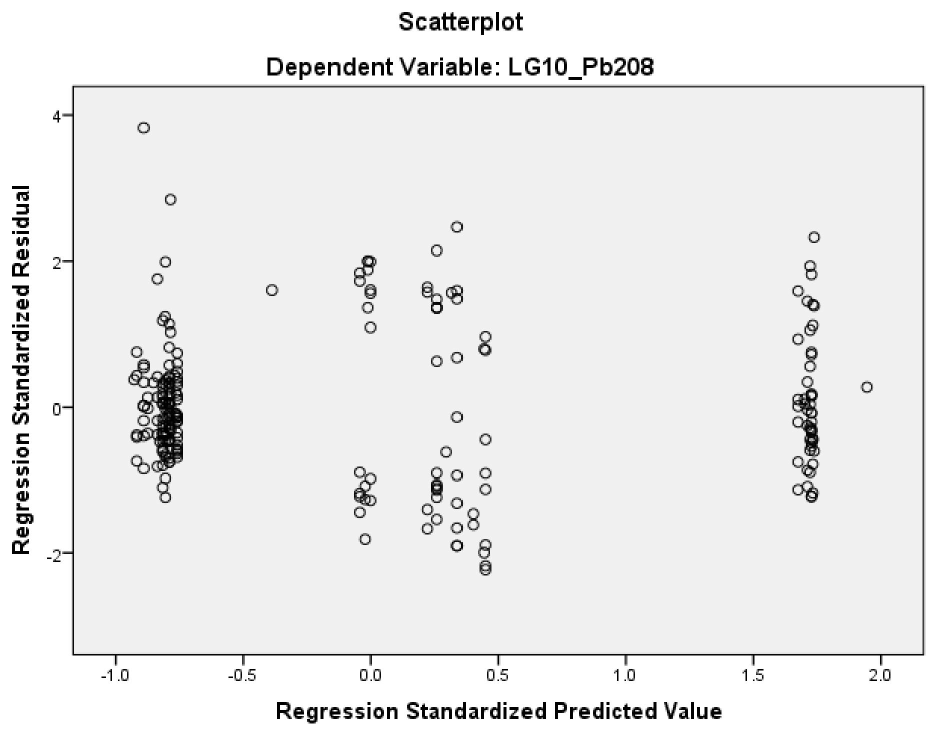

Scatterplot
Dependent Variable: LG10_Pb208
Regression Standardized Residual
Regression Standardized Predicted Value
4
2
0
-2
-1.0
-0.5
0.0
0.5
1.0
1.5
2.0

```
REGRESSION
  /DESCRIPTIVES MEAN STDDEV CORR SIG N
  /MISSING LISTWISE
  /STATISTICS COEFF OUTS CI(95) R ANOVA COLLIN TOL
  /CRITERIA=PIN(.05) POUT(.10)
  /NOORIGIN
  /DEPENDENT Phytic_acid_percent
  /METHOD=STEPWISE Avg_Rain_Flowering_to_harvest Min_Temp_Flowering_to_harvest
    Max_Temp_Flowering_to_harvest Avg_Temp_Flowering_to_harvest
  /SCATTERPLOT=(*ZRESID ,*ZPRED)
  /RESIDUALS HISTOGRAM(ZRESID) NORMPROB(ZRESID)
  /CASEWISE PLOT(ZRESID) OUTLIERS(3)
  /SAVE PRED COOK ZRESID.
```

Regression

Notes

Output Created		23-NOV-2017 17:45:15
Comments		
Input	Data	C:\Users\Brendan\Documents\Micronutrients\Yield and micronutirents variety+condition+season.sav
	Active Dataset	DataSet1
	Filter	<none>
	Weight	<none>
	Split File	<none>
	N of Rows in Working Data File	240
Missing Value Handling	Definition of Missing	User-defined missing values are treated as missing.
	Cases Used	Statistics are based on cases with no missing values for any variable used.

Syntax		REGRESSION<br>/DESCRIPTIVES MEAN STDDEV CORR SIG N<br>/MISSING LISTWISE<br>/STATISTICS COEFF OUTS CI(95) R ANOVA COLLIN TOL<br>/CRITERIA=PIN(.05) POUT(.10)<br>/NOORIGIN<br>/DEPENDENT Phytic_acid_percent<br>/METHOD=STEPWISE Avg_Rain_Flowering_to_harvest Min_Temp_Flowering_to_harvest<br><br>Max_Temp_Flowering_to_harvest Avg_Temp_Flowering_to_harvest<br>/SCATTERPLOT=(*ZRESID ,*ZPRED)<br>/RESIDUALS HISTOGRAM(ZRESID) NORMPROB(ZRESID)<br>/CASEWISE PLOT(ZRESID) OUTLIERS(3)<br>/SAVE PRED COOK ZRESID.
Resources	Processor Time	00:00:00.61
	Elapsed Time	00:00:00.59
	Memory Required	6864 bytes
	Additional Memory Required for Residual Plots	632 bytes
Variables Created or Modified	PRE_6	Unstandardized Predicted Value
	ZRE_6	Standardized Residual
	COO_6	Cook's Distance

Descriptive Statistics

	Mean	Std. Deviation	N
Phytic_acid_percent	1.0544	.31563	229
Avg_Rain_Flowering_to_harvest	54.0352	35.03373	229
Min_Temp_Flowering_to_harvest	10.5860	1.25344	229
Max_Temp_Flowering_to_harvest	32.4891	2.54815	229
Avg_Temp_Flowering_to_harvest	22.5849	1.41650	229

Correlations

		Phytic_acid_percent	Avg_Rain_Flowering_to_harvest	Min_Temp_Flowering_to_harvest	Max_Temp_Flowering_to_harvest	Avg_Temp_Flowering_to_harvest
Pearson Correlation	Phytic_acid_percent	1.000	-.171	.300	.328	.407
	Avg_Rain_Flowering_to_harvest	-.171	1.000	-.323	-.681	-.238
	Min_Temp_Flowering_to_harvest	.300	-.323	1.000	.721	.838
	Max_Temp_Flowering_to_harvest	.328	-.681	.721	1.000	.771
	Avg_Temp_Flowering_to_harvest	.407	-.238	.838	.771	1.000
Sig. (1-tailed)	Phytic_acid_percent	.	.005	.000	.000	.000
	Avg_Rain_Flowering_to_harvest	.005	.	.000	.000	.000
	Min_Temp_Flowering_to_harvest	.000	.000	.	.000	.000
	Max_Temp_Flowering_to_harvest	.000	.000	.000	.	.000
	Avg_Temp_Flowering_to_harvest	.000	.000	.000	.000	.
N	Phytic_acid_percent	229	229	229	229	229
	Avg_Rain_Flowering_to_harvest	229	229	229	229	229
	Min_Temp_Flowering_to_harvest	229	229	229	229	229
	Max_Temp_Flowering_to_harvest	229	229	229	229	229
	Avg_Temp_Flowering_to_harvest	229	229	229	229	229

Variables Entered/Removed[a]

Model	Variables Entered	Variables Removed	Method
1	Avg_Temp_Flowering_to_harvest	.	Stepwise (Criteria: Probability-of-F-to-enter <= .050, Probability-of-F-to-remove >= .100).

a. Dependent Variable: Phytic_acid_percent

Model Summary[b]

Model	R	R Square	Adjusted R Square	Std. Error of the Estimate
1	.407[a]	.166	.162	.28894

a. Predictors: (Constant), Avg_Temp_Flowering_to_harvest

b. Dependent Variable: Phytic_acid_percent

ANOVA[a]

Model		Sum of Squares	df	Mean Square	F	Sig.
1	Regression	3.762	1	3.762	45.057	.000[b]
	Residual	18.952	227	.083		
	Total	22.713	228			

a. Dependent Variable: Phytic_acid_percent

b. Predictors: (Constant), Avg_Temp_Flowering_to_harvest

Coefficients[a]

Model		Unstandardized Coefficients		Standardized Coefficients	t	Sig.	95.0% Confidence Interval for B		Collinearity Statistics	
		B	Std. Error	Beta			Lower Bound	Upper Bound	Tolerance	VIF
1	(Constant)	-.994	.306		-3.250	.001	-1.596	-.391		
	Avg_Temp_Flowering_to_harvest	.091	.014	.407	6.712	.000	.064	.117	1.000	1.000

a. Dependent Variable: Phytic_acid_percent

Excluded Variables[a]

Model		Beta In	t	Sig.	Partial Correlation	Collinearity Statistics		
						Tolerance	VIF	Minimum Tolerance
1	Avg_Rain_Flowering_to_harvest	-.079[b]	-1.266	.207	-.084	.944	1.060	.944
	Min_Temp_Flowering_to_harvest	-.139[b]	-1.251	.212	-.083	.297	3.364	.297
	Max_Temp_Flowering_to_harvest	.034[b]	.361	.718	.024	.405	2.466	.405

a. Dependent Variable: Phytic_acid_percent

b. Predictors in the Model: (Constant), Avg_Temp_Flowering_to_harvest

Collinearity Diagnostics[a]

Model	Dimension	Eigenvalue	Condition Index	Variance Proportions	
				(Constant)	Avg_Temp_Flowering_to_harvest
1	1	1.998	1.000	.00	.00
	2	.002	31.989	1.00	1.00

a. Dependent Variable: Phytic_acid_percent

Casewise Diagnostics[a]

Case Number	Std. Residual	Phytic_acid_percent	Predicted Value	Residual
48	3.220	1.86	.9288	.93041
145	-3.026	.09	.9642	-.87421

a. Dependent Variable: Phytic_acid_percent

Residuals Statistics[a]

	Minimum	Maximum	Mean	Std. Deviation	N
Predicted Value	.9288	1.3278	1.0544	.12845	229
Std. Predicted Value	-.978	2.129	.000	1.000	229
Standard Error of Predicted Value	.019	.045	.026	.008	229
Adjusted Predicted Value	.9208	1.3285	1.0544	.12840	229
Residual	-.87421	.93041	.00000	.28831	229
Std. Residual	-3.026	3.220	.000	.998	229
Stud. Residual	-3.035	3.234	.000	1.002	229
Deleted Residual	-.87996	.93844	.00005	.29071	229
Stud. Deleted Residual	-3.092	3.304	-.001	1.009	229
Mahal. Distance	.000	4.531	.996	1.254	229
Cook's Distance	.000	.053	.004	.008	229
Centered Leverage Value	.000	.020	.004	.005	229

a. Dependent Variable: Phytic_acid_percent

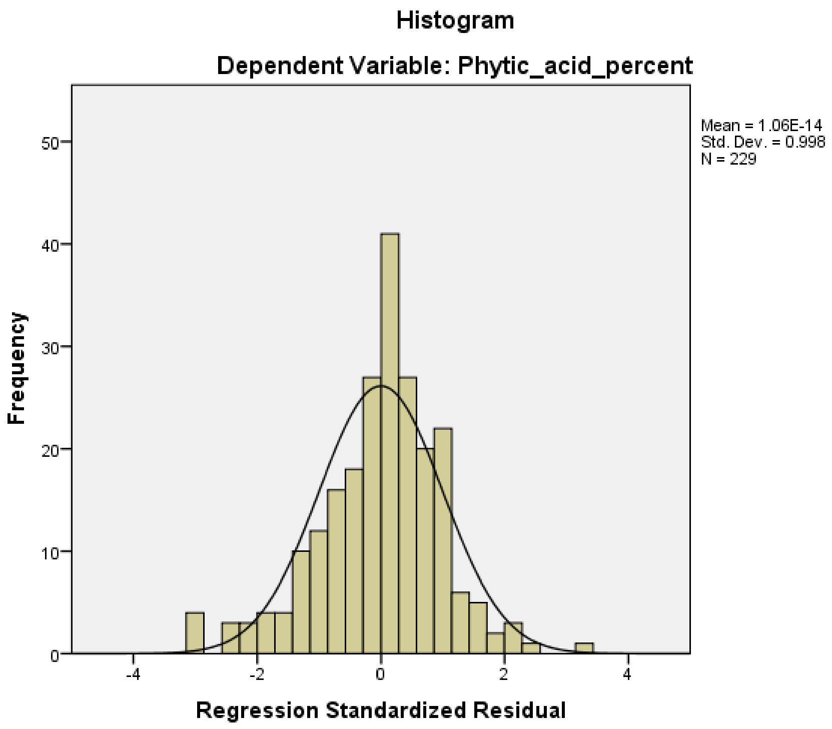
Histogram
Dependent Variable: Phytic_acid_percent
Mean = 1.06E-14
Std. Dev. = 0.998
N = 229
Frequency
50
40
30
20
10
0
-4
-2
0
2
4
Regression Standardized Residual

Charts

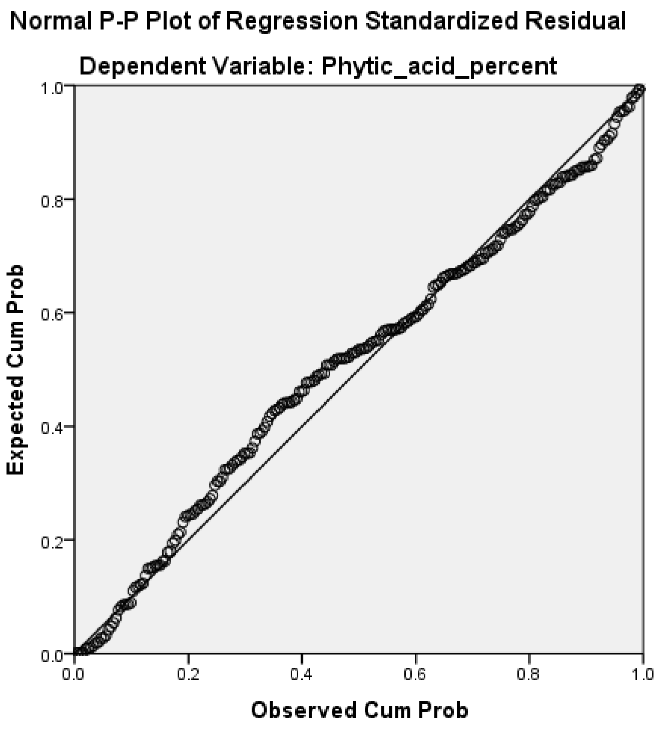
Normal P-P Plot of Regression Standardized Residual
Dependent Variable: Phytic_acid_percent
Expected Cum Prob
Observed Cum Prob
0.0
0.2
0.4
0.6
0.8
1.0

Scatterplot

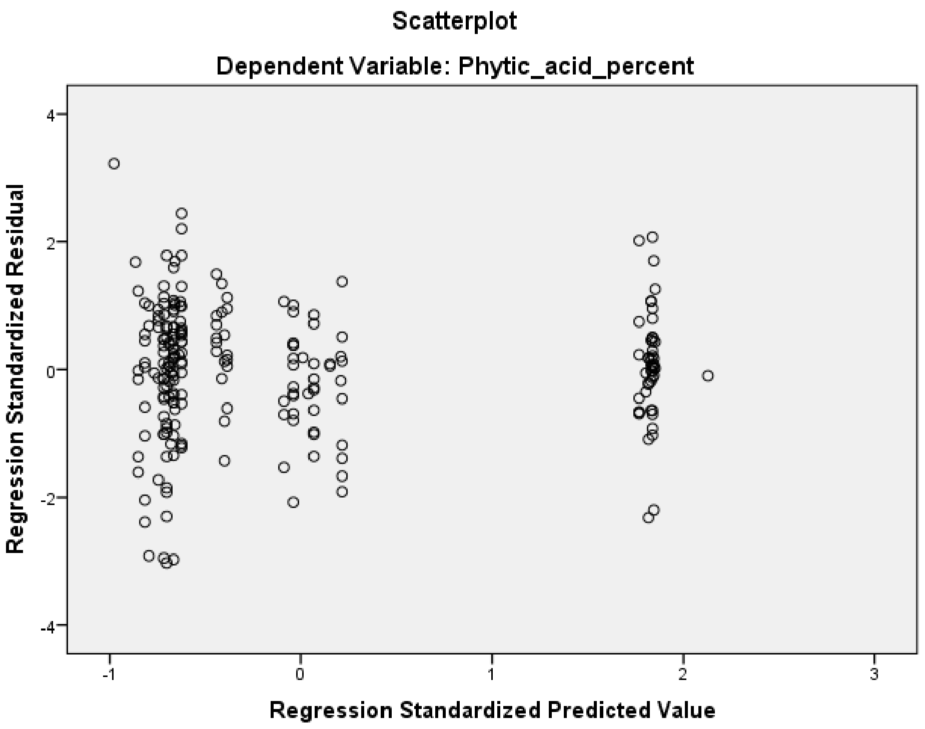


```
REGRESSION
  /DESCRIPTIVES MEAN STDDEV CORR SIG N
  /MISSING LISTWISE
  /STATISTICS COEFF OUTS CI(95) R ANOVA
  /CRITERIA=PIN(.05) POUT(.10)
  /NOORIGIN
```

```
  /DEPENDENT Total_protein_mg_per_g_dw
  /METHOD=STEPWISE Avg_Rain_Flowering_to_harvest Min_Temp_Flowering_to_harvest
    Max_Temp_Flowering_to_harvest Avg_Temp_Flowering_to_harvest
  /SCATTERPLOT=(*ZRESID ,*ZPRED)
  /RESIDUALS HISTOGRAM(ZRESID) NORMPROB(ZRESID).
```

Regression

Notes

Output Created		05-JUN-2018 12:08:33
Comments		
Input	Data	C:\Users\Brendan\Documents\Micronutrients\Yield and micronutirents variety+condition+season.sav
	Active Dataset	DataSet1
	Filter	<none>
	Weight	<none>
	Split File	<none>
	N of Rows in Working Data File	240
Missing Value Handling	Definition of Missing	User-defined missing values are treated as missing.

Cases Used	Statistics are based on cases with no missing values for any variable used.
Syntax	REGRESSION<br>/DESCRIPTIVES MEAN STDDEV CORR SIG N<br>/MISSING LISTWISE<br>/STATISTICS COEFF OUTS CI(95) R ANOVA<br>/CRITERIA=PIN(.05) POUT(.10)<br>/NOORIGIN<br>/DEPENDENT Total_protein_mg_per_g_dw<br>/METHOD=STEPWISE Avg_Rain_Flowering_to_harvest Min_Temp_Flowering_to_harvest<br><br>Max_Temp_Flowering_to_harvest Avg_Temp_Flowering_to_harvest<br>/SCATTERPLOT=(*ZRESID ,*ZPRED)<br>/RESIDUALS HISTOGRAM(ZRESID) NORMPROB(ZRESID).

Resources	Processor Time	00:00:00.69
	Elapsed Time	00:00:00.44
	Memory Required	7792 bytes
	Additional Memory Required for Residual Plots	632 bytes

Descriptive Statistics

	Mean	Std. Deviation	N
Total_protein_mg_per_g_dw	347.2791	76.44644	230
Avg_Rain_Flowering_to_harvest	54.0339	34.95716	230
Min_Temp_Flowering_to_harvest	10.5826	1.25178	230
Max_Temp_Flowering_to_harvest	32.4783	2.54787	230
Avg_Temp_Flowering_to_harvest	22.5812	1.41477	230

Correlations

		Total_protein_mg_per_g_dw	Avg_Rain_Flowering_to_harvest	Min_Temp_Flowering_to_harvest	Max_Temp_Flowering_to_harvest	Avg_Temp_Flowering_to_harvest
Pearson Correlation	Total_protein_mg_per_g_dw	1.000	-.173	.036	.280	-.018
	Avg_Rain_Flowering_to_harvest	-.173	1.000	-.322	-.680	-.237
	Min_Temp_Flowering_to_harvest	.036	-.322	1.000	.722	.839

	Max_Temp_Flowering_to_harvest	.280	-.680	.722	1.000	.771
	Avg_Temp_Flowering_to_harvest	-.018	-.237	.839	.771	1.000
Sig. (1-tailed)	Total_protein_mg_per_g_dw	.	.004	.294	.000	.395
	Avg_Rain_Flowering_to_harvest	.004	.	.000	.000	.000
	Min_Temp_Flowering_to_harvest	.294	.000	.	.000	.000
	Max_Temp_Flowering_to_harvest	.000	.000	.000	.	.000
	Avg_Temp_Flowering_to_harvest	.395	.000	.000	.000	.
N	Total_protein_mg_per_g_dw	230	230	230	230	230
	Avg_Rain_Flowering_to_harvest	230	230	230	230	230
	Min_Temp_Flowering_to_harvest	230	230	230	230	230
	Max_Temp_Flowering_to_harvest	230	230	230	230	230
	Avg_Temp_Flowering_to_harvest	230	230	230	230	230

Variables Entered/Removed[a]

Model	Variables Entered	Variables Removed	Method

1	Max_Temp_Flowering_to_harvest	.	Stepwise (Criteria: Probability-of-F-to-enter <= .050, Probability-of-F-to-remove >= .100).
2	Avg_Temp_Flowering_to_harvest	.	Stepwise (Criteria: Probability-of-F-to-enter <= .050, Probability-of-F-to-remove >= .100).
3	Avg_Rain_Flowering_to_harvest	.	Stepwise (Criteria: Probability-of-F-to-enter <= .050, Probability-of-F-to-remove >= .100).

a. Dependent Variable: Total_protein_mg_per_g_dw

Model Summary[d]

Model	R	R Square	Adjusted R Square	Std. Error of the Estimate
1	.280[a]	.078	.074	73.55268
2	.462[b]	.213	.206	68.11086
3	.560[c]	.314	.305	63.74760

a. Predictors: (Constant), Max_Temp_Flowering_to_harvest

b. Predictors: (Constant), Max_Temp_Flowering_to_harvest, Avg_Temp_Flowering_to_harvest

c. Predictors: (Constant), Max_Temp_Flowering_to_harvest, Avg_Temp_Flowering_to_harvest, Avg_Rain_Flowering_to_harvest

d. Dependent Variable: Total_protein_mg_per_g_dw

ANOVA[a]

Model		Sum of Squares	df	Mean Square	F	Sig.
1	Regression	104810.160	1	104810.160	19.373	.000[b]
	Residual	1233479.227	228	5409.997		
	Total	1338289.387	229			
2	Regression	285216.049	2	142608.024	30.741	.000[c]
	Residual	1053073.338	227	4639.090		
	Total	1338289.387	229			
3	Regression	419880.457	3	139960.152	34.441	.000[d]
	Residual	918408.931	226	4063.756		
	Total	1338289.387	229			

a. Dependent Variable: Total_protein_mg_per_g_dw

b. Predictors: (Constant), Max_Temp_Flowering_to_harvest

c. Predictors: (Constant), Max_Temp_Flowering_to_harvest, Avg_Temp_Flowering_to_harvest

d. Predictors: (Constant), Max_Temp_Flowering_to_harvest, Avg_Temp_Flowering_to_harvest, Avg_Rain_Flowering_to_harvest

Coefficients[a]

Model	Unstandardized Coefficients	Standardized Coefficients	t	Sig.	95.0% Confidence Interval for B

		B	Std. Error	Beta			Lower Bound	Upper Bound
1	(Constant)	74.571	62.147		1.200	.231	-47.886	197.027
	Max_Temp_Flowering_to_harvest	8.397	1.908	.280	4.402	.000	4.638	12.156
2	(Constant)	344.850	72.044		4.787	.000	202.889	486.811
	Max_Temp_Flowering_to_harvest	21.753	2.776	.725	7.835	.000	16.282	27.224
	Avg_Temp_Flowering_to_harvest	-31.180	5.000	-.577	-6.236	.000	-41.032	-21.328
3	(Constant)	98.584	79.855		1.235	.218	-58.772	255.940
	Max_Temp_Flowering_to_harvest	41.969	4.369	1.399	9.607	.000	33.360	50.577
	Avg_Temp_Flowering_to_harvest	-52.222	5.938	-.966	-8.794	.000	-63.924	-40.521
	Avg_Rain_Flowering_to_harvest	1.201	.209	.549	5.757	.000	.790	1.612

a. Dependent Variable: Total_protein_mg_per_g_dw

Excluded Variables[a]

Model		Beta In	t	Sig.	Partial Correlation	Collinearity Statistics Tolerance
1	Avg_Rain_Flowering_to_harvest	.033[b]	.376	.707	.025	.538
	Min_Temp_Flowering_to_harvest	-.347[b]	-3.891	.000	-.250	.479

	Avg_Temp_Flowering_to_harvest	-.577[b]	-6.236	.000	-.382	.405
2	Avg_Rain_Flowering_to_harvest	.549[c]	5.757	.000	.358	.334
	Min_Temp_Flowering_to_harvest	-.013[c]	-.114	.909	-.008	.283
3	Min_Temp_Flowering_to_harvest	.049[d]	.467	.641	.031	.280

a. Dependent Variable: Total_protein_mg_per_g_dw

b. Predictors in the Model: (Constant), Max_Temp_Flowering_to_harvest

c. Predictors in the Model: (Constant), Max_Temp_Flowering_to_harvest, Avg_Temp_Flowering_to_harvest

d. Predictors in the Model: (Constant), Max_Temp_Flowering_to_harvest, Avg_Temp_Flowering_to_harvest, Avg_Rain_Flowering_to_harvest

Residuals Statistics[a]

	Minimum	Maximum	Mean	Std. Deviation	N
Predicted Value	288.9461	541.9881	347.2791	42.81984	230
Residual	-241.26813	219.45865	.00000	63.32866	230
Std. Predicted Value	-1.362	4.547	.000	1.000	230
Std. Residual	-3.785	3.443	.000	.993	230

a. Dependent Variable: Total_protein_mg_per_g_dw

Charts

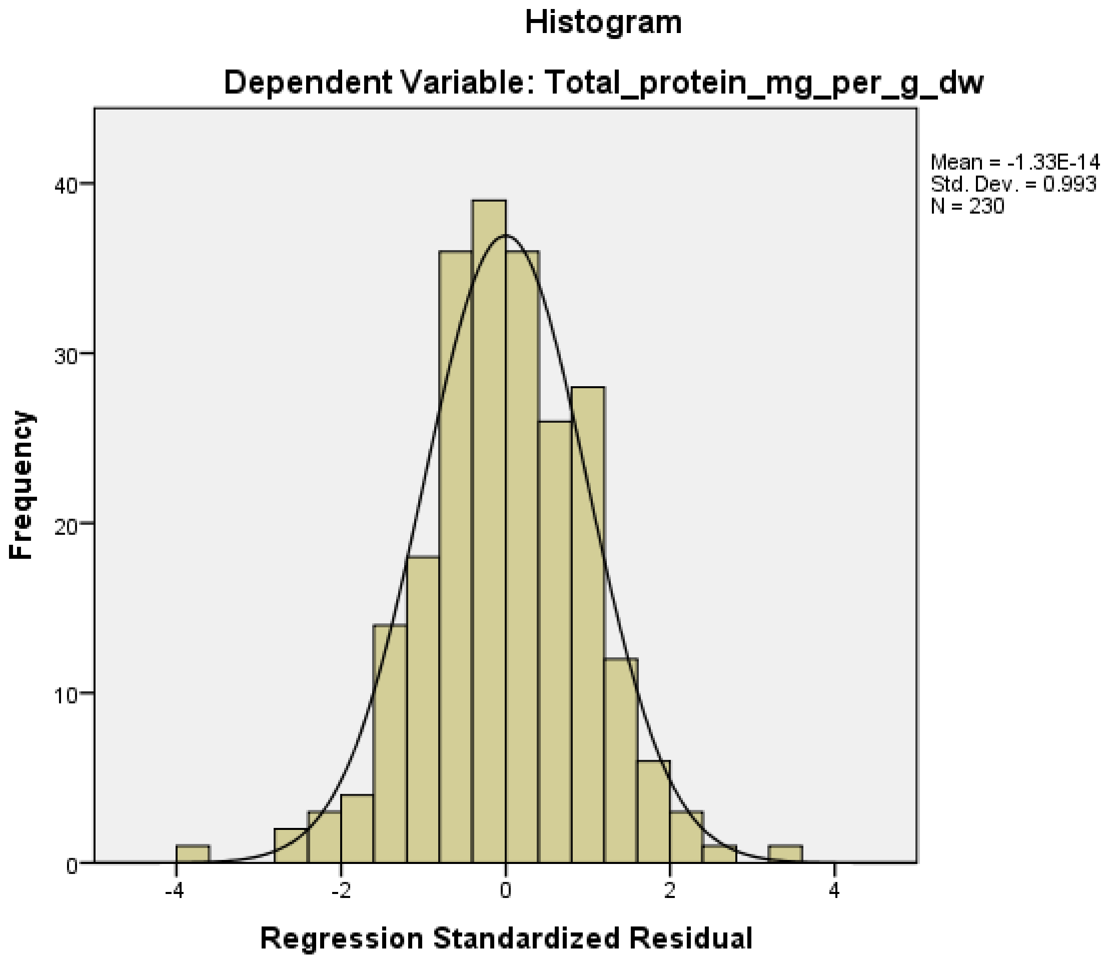
Histogram
Dependent Variable: Total_protein_mg_per_g_dw
Mean = -1.33E-14
Std. Dev. = 0.993
N = 230
Frequency
40
30
20
10
0
-4
-2
0
2
4
Regression Standardized Residual

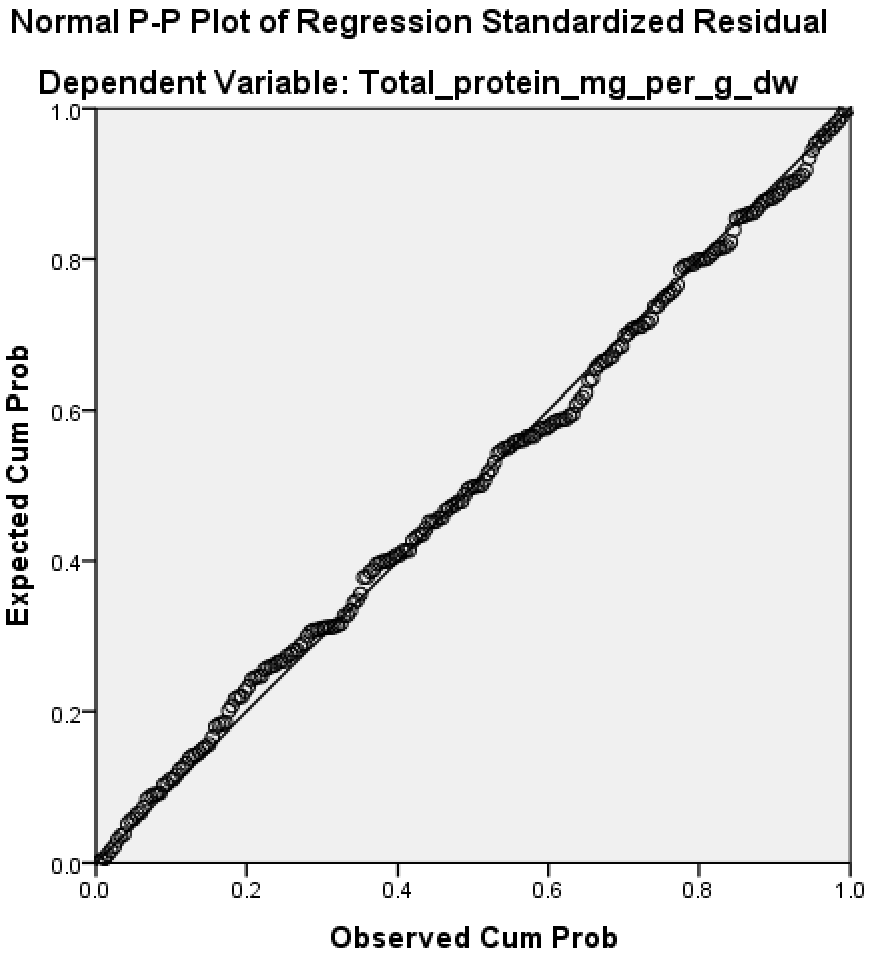
Normal P-P Plot of Regression Standardized Residual
Dependent Variable: Total_protein_mg_per_g_dw
Expected Cum Prob
Observed Cum Prob
0.0
0.2
0.4
0.6
0.8
1.0

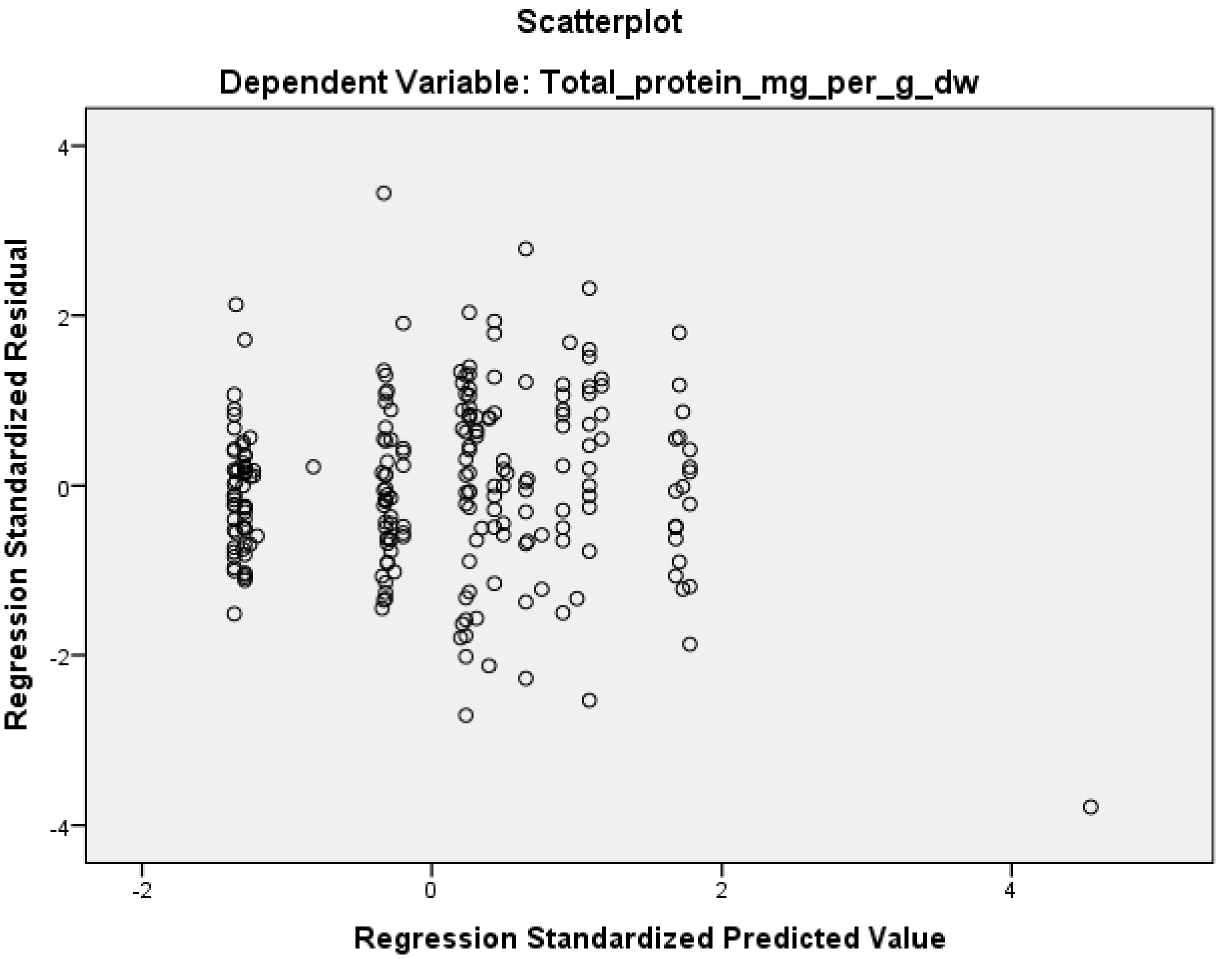

Scatterplot
Dependent Variable: Total_protein_mg_per_g_dw
Regression Standardized Residual
4
2
0
-2
-4
-2
0
2
4
Regression Standardized Predicted Value
